# Excess Google Searches for Child Abuse and Intimate Partner Violence During the COVID-19 Pandemic: Infoveillance Approach

**DOI:** 10.2196/36445

**Published:** 2022-06-13

**Authors:** Corinne A Riddell, Krista Neumann, N Jeanie Santaularia, Kriszta Farkas, Jennifer Ahern, Susan M Mason

**Affiliations:** 1 Division of Biostatistics School of Public Health University of California Berkeley, CA United States; 2 Division of Epidemiology School of Public Health University of California Berkeley, CA United States; 3 Division of Epidemiology and Community Health School of Public Health University of Minnesota Minneapolis, MN United States

**Keywords:** child abuse, household violence, infoveillance, violence, domestic violence, abuse, Google, COVID-19

## Abstract

**Background:**

The COVID-19 pandemic has created environments with increased risk factors for household violence, such as unemployment and financial uncertainty. At the same time, it led to the introduction of policies to mitigate financial uncertainty. Further, it hindered traditional measurements of household violence.

**Objective:**

Using an infoveillance approach, our goal was to determine if there were excess Google searches related to exposure to child abuse, intimate partner violence (IPV), and child-witnessed IPV during the COVID-19 pandemic and if any excesses are temporally related to shelter-in-place and economic policies.

**Methods:**

Data on relative search volume for each violence measure was extracted using the Google Health Trends application programming interface for each week from 2017 to 2020 for the United States. Using linear regression with restricted cubic splines, we analyzed data from 2017 to 2019 to characterize the seasonal variation shared across prepandemic years. Parameters from prepandemic years were used to predict the expected number of Google searches and 95% prediction intervals (PI) for each week in 2020. Weeks with searches above the upper bound of the PI are in excess of the model’s prediction.

**Results:**

Relative search volume for exposure to child abuse was greater than expected in 2020, with 19% (10/52) of the weeks falling above the upper bound of the PI. These excesses in searches began a month after the Pandemic Unemployment Compensation program ended. Relative search volume was also heightened in 2020 for child-witnessed IPV, with 33% (17/52) of the weeks falling above the upper bound of the PI. This increase occurred after the introduction of shelter-in-place policies.

**Conclusions:**

Social and financial disruptions, which are common consequences of major disasters such as the COVID-19 pandemic, may increase risks for child abuse and child-witnessed IPV.

## Introduction

Child abuse and intimate partner violence (IPV) are common. In the United States, 37% of children will be involved in an official investigation by Child Protective Services, whereas 25% of women and 11% of men experience IPV [1,2]. The downstream effects of abuse are profound; compared to adults not reporting histories of abuse, adults with histories of abuse are 60% more likely to abuse drugs, 60% more likely to develop cardiovascular disease, and 3 times more likely to attempt suicide, demonstrating the wide-ranging effects on health across the life course [3,4]. These increased risks of adverse adult health outcomes are hypothesized to be mediated through several pathways such as increased high-risk behaviors (eg, substance abuse, smoking, and exercise avoidance), dysregulated immune functioning, and psychiatric disorders [5].

The risk factors for perpetrating child abuse and IPV include (but are not limited to) undergoing economic stress, feelings of isolation and disconnection, and parental stress (for child abuse) [6,7]. These risk factors were magnified during the first year of the COVID-19 pandemic through increased unemployment, shelter-in-place (SIP) policies, and remote schooling. Previous studies found that calls to the US hotline *Childhelp* increased during the first year of the pandemic, as did arrests, calls, and reports to police departments related to domestic violence [8,9]. At the same time, policy responses to mitigate financial uncertainty in the United States were substantial. For example, the Pandemic Unemployment Compensation (PUC) program increased unemployment payments by US $600 per week for 4 months, which offered an opportunity to explore the potential protective impacts of policies mitigating financial uncertainty. A challenge, however, is that the pandemic hindered the measurement of violence through traditional measures, for example, by reducing interactions with mandated reporters of child maltreatment [10]. A previous study found that during the Great Recession, places with decreases in Child Protective Services referrals had increases in both child mortality and Google searches for child abuse [11]. The divergence of reports from other measures of abuse suggests that abuse surveillance based on referrals may be hindered during periods of economic upheaval and that Google searches may help overcome this limitation.

We considered a broad set of Google searches based on the terms that individuals experiencing or witnessing child abuse or IPV would use as a measure of the incidence of household violence. This approach to monitoring epidemiologic trends falls under the field of “infoveillance,” where user-generated data collected from the internet and social media sites are used for surveillance [12,13]. Peaks in Google searches related to domestic violence were found to occur in the same months as peaks in police calls for domestic violence, suggesting that Google searches may offer a promising way to measure household violence outcomes [14]. The use of Google searches to measure epidemiologic outcomes has varied; searches related to influenza did not track well with the incidence of influenza-like illnesses [15-17], but searches related to the loss of smell correlated strongly with COVID-19 cases and deaths in the first months of the first wave of the pandemic [18]. Given that the pandemic hindered the measurement of violence in conjunction with similar trends of domestic violence with Google searches, the infoveillance approach is well-suited to study violence during a time of uncertainty.

The objective of this study was to establish whether Google searches for child abuse and IPV, which are nontraditional violence measures, increased during the pandemic and consider the timing of the increases in relation to SIP and economic policies that may affect violence risk factors. The findings will have implications for future policy responses to major crises.

## Methods

### Data Collection

To measure exposure to child abuse, child-witnessed IPV, and exposure to IPV using search data, we created 3 lists of queries that individuals who experience or witness abuse may search for on the internet (see Multimedia Appendix 1). Our methodology has been used in a previous study (Neumann et al, unpublished data, 2022). We conducted a review of the literature to determine how children and adults discuss these experiences (eg, Foster and Hagedorn [19]). We also considered the language used in validated scales measuring violence. We then tested the sensitivity and specificity of these search phrases by searching for them using a Google Incognito browser to ensure that the results were consistent with those experiencing or witnessing abuse and discarded the search phrases that did not appear relevant. We settled on 3 final search terms, each of which combined phrases specific to an abuse subtype (ie, exposure to child abuse, child-witnessed IPV, and exposure to IPV).

The Google Health Trends application programming interface (API) was used to obtain the Google search volume for 3 separate violence measures: exposure to child abuse, child-witnessed IPV, and exposure to IPV. To obtain the search data from the API, the researcher must first apply for an API key. Search terms, geographic region, and the time period of interest must be entered by the researcher, and the API will then return the probability of the search terms for the specified geo-time period. The returned results are based on a random sample of all Google searches and then, for readability, scaled by 10 million (2020 *Google Health Trends API Getting Started Guide*, unpublished document provided with API key). The API output must be interpreted as a relative search volume with an unknown denominator as the total number of searches used to calculate the returned probability is unknown to researchers. For this study, we obtained national-level weekly search volumes for each of our 3 search terms from 2017 to 2020 in the United States. We chose this geo-temporal resolution so that we could assess trends relative to important federal policy changes. All data were retrieved from the API between July 6 and 24, 2021. Since the returned values are probabilities based on a random sample of all Google searches, it is also important to account for sampling variability. To obtain more stable search volume estimates, 10 samples of each search were extracted. We computed both the mean and median of the estimates; their difference was very small, so we used the mean in the model.

### Statistical Model

We first built a prediction model using data from 2017 to 2019. Using a linear regression fit using ordinary least squares, we modeled weekly Google search volume based on date, entered with a main effect term (to control for linear increases [or decreases] in Google search volume over the prediction period) and a restricted cubic spline for the week of year (with interior knots at the 10th, 50th, and 90th percentiles) to capture seasonal patterning. We report the adjusted r-squared value to quantify the amount of outcome variation that is captured by the model.

We then used the model to predict the expected Google search volume for each week in 2020 alongside its 95% prediction intervals (PI). PIs place bounds on where observed individual values are expected to fall [20]. Thus, observations from 5% (2-3 weeks) of the 52 weeks in 2020 are expected to fall outside of the bounds of the 95% PI, and any more than that is considered a notable finding that is not predicted given the previous trends in the search volume.

We plotted weekly search volume, overlayed with the predicted searches and 95% PI. We annotated these plots with information about policies and payments that may ameliorate or accentuate risk factors for abuse, including the introduction of state-specific SIP policies (starting March 19, 2020 [21,22]), the date when individuals started receiving one-time Economic Impact Payments (April 17, 2020 [23]), and the end date of the PUC program (July 31, 2020 [23]), which provided an additional US $600 per week to claimants on top of usual unemployment benefits.

The R statistical software (version 4.1.0; R Foundation for Statistical Computing) was used to conduct this analysis. All code can be found in the GitHub repository [24].

### Ethical Considerations

No personal information is available to researchers through the Google Health Trends API (2020 Google Health Trends API Getting Started Guide, unpublished document provided with API key). Google search volumes below an unspecified lower bound are suppressed by Google and not made available to researchers.

## Results

### Google Search Volumes

Yearly average Google search volumes for the abuse outcomes ranged between volumes of 58.3 (child-witnessed IPV in 2019) and 87.0 (exposure to child abuse in 2020; Table 1 and Figures S1-3 in Multimedia Appendix 2). All models met the assumptions required for linear regression (Figures S4-6 in Multimedia Appendix 2).

**Table 1:** Yearly average Google search volume and SD for exposure to child abuse, child-witnessed intimate partner violence (IPV), and exposure to IPV.

Domain of abuse	Google search volume, mean (SD)			
	2017	2018	2019	2020
Exposure to child abuse	85.0 (9.4)	79.0 (7.5)	83.8 (6.3)	87.0 (8.2)
Child-witnessed IPV	60.0 (7.0)	58.7 (6.4)	58.3 (6.0)	64.0 (8.7)
Exposure to IPV	80.7 (5.9)	85.6 (6.2)	82.1 (7.3)	80.1 (7.2)

### Exposure to Child Abuse

**Figure 1:**
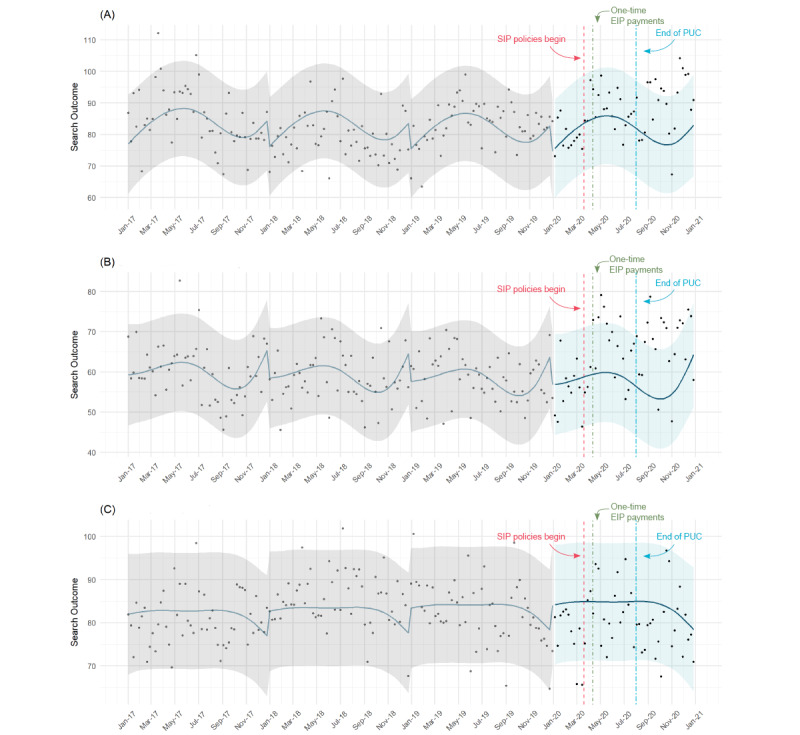
Average weekly Google search volume (points) alongside predicted Google search volume (curve) and 95% prediction intervals (grey and blue bands) for (A) exposure to child abuse, (B) child-witnessed intimate partner violence, and (C) exposure to intimate partner violence, United States, 2017-2020. State-specific shelter-in-place (SIP) policies began on March 19, 2020, with California’s SIP order, shortly after the national emergency was declared on March 13, 2020. The Coronavirus Aid, Relief, and Economic Security (CARES) Act was enacted on March, 27, 2020, and one-time Economic Impact Payments (EIP) were sent to nearly 90 million individuals by April 17, 2020, as part of the CARES Act. The Pandemic Unemployment Compensation (PUC) program, which was also part of the CARES act and provided an additional US $600 per week to claimants on top of usual unemployment benefits, expired on July 31, 2020.

Over the time period from 2017 to 2019, the Google search volume for child abuse was consistently highest in June and lowest in January, and the search volume decreased slightly year-to-year (Figure 1A). The model explained 15.3% of the variation in child abuse searches. From 2017 to 2019 inclusive, 5 out of 157 (3.2%) weeks fell outside of the PI, as expected, with 2 points above and 3 points below the upper and lower bounds, respectively. In 2020, 10 out of 52 (19%) weeks fell above the PI, all above the upper bound, suggesting an increase in child abuse searches. These increases were detected beginning August 30, 2020, about 4 weeks after the end of the PUC program.

### Child-Witnessed IPV

From 2017 to 2019, the Google search volume was consistently highest in December and lowest in October, and the search volume declined slightly year-to-year (Figure 1B). The model explained 11.4% of the variation in child-witnessed IPV searches. From 2017 to 2019 inclusive, 6 out of 157 (3.8%) weeks occurred outside of the PI (3 points each above and below the upper and lower bounds), as expected, while in 2020, 17 out of 52 (33%) fell above the PI, suggesting an increase in child-witnessed IPV searches. All increases occurred after SIP policies began and continued after the end of the PUC program.

### Exposure to IPV

From 2017 to 2019, IPV searches showed dips in November and December, with an otherwise flat yearly trend (Figure 1C). The model explained only 1.8% of the variation in the Google search volume for exposure to IPV. The model detected 3 out of 52 (6%) weeks with lower-than-expected search volumes in 2020, which was similar to prepandemic years (5%, 8/157).

## Discussion

### Principal Findings

Overall, we found that following the start of the COVID-19 pandemic, child abuse and child-witnessed IPV searches were elevated beyond that predicted by search history (from 2017 to 2019) for a substantial fraction of months/weeks. Child abuse searches increased a month after the PUC program ended. This pattern would be consistent with the hypothesis that the substantial loss in income from the end of the PUC program may have led to an increase in child abuse; this would be valuable to examine in future research. These findings are consistent with previous literature linking decreased family income downstream of policy changes to increased reports to Child Protective Services [25-27]. Child-witnessed IPV searches, but not exposure to IPV searches, increased at the time of SIP policies. This might suggest greater opportunities for children to witness IPV rather than an increase in IPV itself, although searches for IPV itself might have been impacted by less privacy with household members spending more time together. The findings of increases in child abuse and child-witnessed IPV align with documented increases in calls to *ChildHelp* and police reports for domestic violence [8,9]. In the first study, calls to the hotline *Childhelp* increased 14% during 2020 compared to 2019 [8]. In the second study, increases between 10% to 27% were reported in the number of arrests, calls, or reports to police departments related to domestic violence [9]. This study is important because Google search data are a promising alternative to traditional measures of child abuse and IPV, which have well-documented reporting biases. Surveys estimate that less than 10% of child abuse [28] and less than half of domestic abuse is reported [29], implying that the majority of abuse goes undetected using traditional measures. The consistency of our findings with research that used hotline and police report data adds to the limited evidence on the impacts of the pandemic on child abuse and IPV and supports the promise of this approach to measuring abuse. Google search data may become particularly salient for this purpose at times when traditional detection approaches may be disrupted.

### Limitations

Our study has limitations. First, we did not directly measure child abuse or IPV, and this study assumes that Google searches for child abuse and IPV track with the underlying incidence of the outcomes. A previous study found that Google searches for domestic abuse were associated with police calls for domestic violence in Finland [14], but no studies have examined this link in the United States. A second limitation is that Google searches can only be performed by individuals with access to the internet. Thus, the results may not generalize to households with no internet access, especially if the effect of the pandemic on abuse was larger or smaller compared to households with internet access. These results also do not reflect the experiences of children who do not use the internet, and thus may correspond more to the experiences of older children. Although some studies have found that Google searches can be affected by mass media related to the topic [30], we attempted to overcome this by limiting searches to those made by individuals experiencing or witnessing abuse, rather than focusing on broad searches like “child abuse” that may track with high-profile cases of abuse. We also removed terms that returned Google search results that were not relevant to exposure to abuse or child-witnessed IPV as part of a process we developed to use Google searches to measure epidemiologic constructs (Neumann et al, unpublished data, 2022). Lastly, the analytic approach we used can be hindered by multiple testing, since we deemed that a week of Google search volume was notable if it fell outside of the PI and we do this for each week in 2020. However, we found that multiple weeks—serially located in time—fall above the PI, which does not seem to suggest that we were detecting a “one-off” that happens to fall outside the PI. Thus, we do not believe that multiple testing played a role in these findings.

### Conclusions

Social and financial disruptions, which are common consequences of major disasters, may increase the risks for child abuse and child-witnessed IPV. The increase in child abuse searches after the abrupt loss of income when PUC payments ceased suggests that economic mitigation strategies may be protective if sustained, though this study did not establish causation. Public health responses to future disasters should incorporate strategies to mitigate household violence.

## Appendix

Multimedia Appendix 1 Search terms.

Multimedia Appendix 2 Maps of average search volume and regression model assumptions.

## Abbreviations

API: application programming interface
IPV: intimate partner violence
PI: prediction intervals
PUC: Pandemic Unemployment Compensation
SIP: shelter-in-place

## References

[1] Kim H, Wildeman C, Jonson-Reid M, Drake B (2017). Lifetime prevalence of investigating child maltreatment among US children. Am J Public Health.

[2] Smith SG, Zhang X, Basile KC, Merrick MT, Wang J, Kresnow MJ, Chen J (2018). National Intimate Partner and Sexual Violence Survey: 2015 data brief - updated release. National Center for Injury Prevention and Control, Centers for Disease Control and Prevention.

[3] Norman RE, Byambaa M, De R, Butchart A, Scott J, Vos T (2012). The long-term health consequences of child physical abuse, emotional abuse, and neglect: a systematic review and meta-analysis. PLoS Med.

[4] Rich-Edwards JW, Mason S, Rexrode K, Spiegelman D, Hibert E, Kawachi I, Jun HJ, Wright RJ (2012). Physical and sexual abuse in childhood as predictors of early-onset cardiovascular events in women. Circulation.

[5] Sachs-Ericsson N, Cromer K, Hernandez A, Kendall-Tackett K (2009). A review of childhood abuse, health, and pain-related problems: the role of psychiatric disorders and current life stress. J Trauma Dissociation.

[6] (2021). Risk and protective factors for perpetration. Centers for Disease Control and Prevention.

[7] (2022). Risk and protective factors. Centers for Disease Control and Prevention.

[8] Ortiz R, Kishton R, Sinko L, Fingerman M, Moreland D, Wood J, Venkataramani A (2021). Assessing child abuse hotline inquiries in the wake of COVID-19: answering the call. JAMA Pediatr.

[9] Boserup B, McKenney M, Elkbuli A (2020). Alarming trends in US domestic violence during the COVID-19 pandemic. Am J Emerg Med.

[10] Welch M, Haskins R (2020). What COVID-19 means for America's child welfare system. Brookings.

[11] Stephens-Davidowitz S (2013). Unreported victims of an economic downturn. Squarespace.

[12] Eysenbach G (2011). Infodemiology and infoveillance tracking online health information and cyberbehavior for public health. Am J Prev Med.

[13] Eysenbach G (2009). Infodemiology and infoveillance: framework for an emerging set of public health informatics methods to analyze search, communication and publication behavior on the Internet. J Med Internet Res.

[14] Koutaniemi EM, Einiö Elina (2021). Seasonal variation in seeking help for domestic violence based on Google search data and Finnish police calls in 2017. Scand J Public Health.

[15] Lazer D, Kennedy R, King G, Vespignani A (2014). The parable of Google Flu: traps in big data analysis. Science.

[16] Pollett S, Boscardin WJ, Azziz-Baumgartner E, Tinoco YO, Soto G, Romero C, Kok J, Biggerstaff M, Viboud C, Rutherford GW (2017). Evaluating Google flu trends in Latin America: important lessons for the next phase of digital disease detection. Clin Infect Dis.

[17] Lohr S (2014). Google flu trends: the limits of big data. Bits Blog.

[18] Walker A, Hopkins C, Surda P (2020). Use of Google Trends to investigate loss-of-smell-related searches during the COVID-19 outbreak. Int Forum Allergy Rhinol.

[19] Foster JM, Hagedorn WB (2014). Through the eyes of the wounded: a narrative analysis of children's sexual abuse experiences and recovery process. J Child Sex Abus.

[20] Kleinbaum DG, Kupper LL, Nizam A, Rosenberg ES (2014). 5.10 Prediction of a new value of Y at X0. Applied Regression Analysis and Other Multivariable Models. 5th ed.

[21] Mervosh S, Lu D, Swales V (2020). See which states and cities have told residents to stay at home. New York Times.

[22] Raifman J, Nocka K, Jones D, Bor J, Lipson S, Jay J (2021). COVID-19 US state policy database (CUSP).

[23] Han J, Meyer BD, Sullivan JX (2020). Income and poverty in the COVID-19 pandemic. National Bureau of Economic Research.

[24] Github.

[25] McLaughlin M (2017). Less money, more problems: how changes in disposable income affect child maltreatment. Child Abuse Negl.

[26] Brooks-Gunn J, Schneider W, Waldfogel J (2013). The Great Recession and the risk for child maltreatment. Child Abuse Negl.

[27] Kovski NL, Hill HD, Mooney SJ, Rivara FP, Morgan ER, Rowhani-Rahbar A (2021). Association of state-level earned income tax credits with rates of reported child maltreatment, 2004-2017. Child Maltreat.

[28] MacMillan HL, Jamieson E, Walsh CA (2003). Reported contact with child protection services among those reporting child physical and sexual abuse: results from a community survey. Child Abuse Negl.

[29] Morgan RE, Truman JL (2020). Criminal victimization, 2019. U.S. Department of Justice.

[30] Cervellin G, Comelli I, Lippi G (2017). Is Google Trends a reliable tool for digital epidemiology? Insights from different clinical settings. J Epidemiol Glob Health.

