# Supplementary material for: Excess Google Searches for Child Abuse and Intimate Partner Violence During the COVID-19 Pandemic: Infoveillance Approach
Source: J Med Internet Res. 2022 Jun 13;24(6):e36445. doi: 10.2196/36445 (PMC9202515; doi:10.2196/36445)
Supplement: Multimedia Appendix 1 [file jmir_v24i6e36445_app1.docx]

**Appendix S1 to the paper “Excess Google Searches for Child Abuse and Intimate Partner Violence During the Covid-19 Pandemic”**

**Appendix Overview**

Due to data repression from the Google Health Trends API for search queries that were too specific, we opted to create a single query that encapsulated all of the abuse subtypes of interest. Individual terms for each abuse type (i.e. “mom hit me”, “dad hit me”, etc.) were concatenated using the Boolean or operator “+.” The API considers each individual term between each “+” its own search and combines the results of each of them into the single abuse outcome (either intimate partner violence or child abuse) used. When deciding which individual terms to include in our query, we were cognizant that the API results include all searches containing any of the words in the term, in any order. For example the term “mom hits me” would also return results for the search “mom always hits me” and “when my mom hits it hurts me”. By default, no misspellings, spelling variations, synonyms, plural, or singular versions of the included words are included. To include them, we expanded the query to include various tenses, conjugations and common misspellings.

| **Domain of abuse** | **Page number** |
| --- | --- |
| Intimate partner violence victimization search terms: |  |
| Physical abuse ………....……………………………............ | 1 |
| Emotional abuse ………………...…………………………… | 5 |
| Sexual abuse ………………….....…………………………... | 11 |
| Other searches ……………..…………….....……................ | 15 |
| Child-witnessed intimate partner violence search terms: |  |
| Physical abuse ………....……………………………............ | 17 |
| Emotional abuse ………………...…………………………… | 31 |
| Other searches ……………..…………….....……................ | 39 |
| Child abuse victimization search terms: |  |
| Physical abuse ………....……………………………............ | 43 |
| Emotional abuse ………………...…………………………… | 50 |
| Sexual abuse ………………….....…………………………... | 57 |
| Other searches ……………..…………….....……................ | 69 |

**Intimate partner violence victimization search terms**

**Physical Abuse**

husband held gun to my head + husband pointed gun at me + husband points gun at me + boyfriend held gun to my head + boyfriend pointed gun at me + boyfriend points gun at me + ex-boyfriend held gun to my head + ex-boyfriend pointed gun at me + ex-boyfriend points gun at me + ex-husband held gun to my head + ex-husband pointed gun at me + ex-husband points gun at me + ex boyfriend held gun to my head + ex boyfriend pointed gun at me + ex boyfriend points gun at me + ex husband held gun to my head + ex husband pointed gun at me + ex husband points gun at me + exboyfriend held gun to my head + exboyfriend pointed gun at me + exboyfriend points gun at me + exhusband held gun to my head + exhusband pointed gun at me + exhusband points gun at me + husband slam me + husband slams me + husband slammed me + boyfriend slam me + boyfriend slams me + boyfriend slammed me + ex-boyfriend slam me + ex-boyfriend slams me + ex-boyfriend slammed me + ex-husband slam me + ex-husband slams me + ex-husband slammed me + ex boyfriend slam me + ex boyfriend slams me + ex boyfriend slammed me + ex husband slam me + ex husband slams me + ex husband slammed me + exboyfriend slam me + exboyfriend slams me + exboyfriend slammed me + exhusband slam me + exhusband slams me + exhusband slammed me + husband strangle me + husband strangles me + husband strangled me + boyfriend strangle me + boyfriend strangles me + boyfriend strangled me + ex-boyfriend strangle me + ex-boyfriend strangles me + ex-boyfriend strangled me + ex-husband strangle me + ex-husband strangles me + ex-husband strangled me + ex boyfriend strangle me + ex boyfriend strangles me + ex boyfriend strangled me + ex husband strangle me + ex husband strangles me + ex husband strangled me + exboyfriend strangle me + exboyfriend strangles me + exboyfriend strangled me + exhusband strangle me + exhusband strangles me + exhusband strangled me + husband punch me + husband punches me + husband punched me + boyfriend punch me + boyfriend punches me + boyfriend punched me + ex-boyfriend punch me + ex-boyfriend punches me + ex-boyfriend punched me + ex-husband punch me + ex-husband punches me + ex-husband punched me + ex boyfriend punch me + ex boyfriend punches me + ex boyfriend punched me + ex husband punch me + ex husband punches me + ex husband punched me + exboyfriend punch me + exboyfriend punches me + exboyfriend punched me + exhusband punch me + exhusband punches me + exhusband punched me + husband slap me + husband slaps me + husband slapped me + boyfriend slap me + boyfriend slaps me + boyfriend slapped me + ex-boyfriend slap me + ex-boyfriend slaps me + ex-boyfriend slapped me + ex-husband slap me + ex-husband slaps me + ex-husband slapped me + ex boyfriend slap me + ex boyfriend slaps me + ex boyfriend slapped me + ex husband slap me + ex husband slaps me + ex husband slapped me + exboyfriend slap me + exboyfriend slaps me + exboyfriend slapped me + exhusband slap me + exhusband slaps me + exhusband slapped me + husband beat me + husband beats me + boyfriend beat me + boyfriend beats me + ex-boyfriend beat me + ex-boyfriend beats me + ex-husband beat me + ex-husband beats me + ex boyfriend beat me + ex boyfriend beats me + ex husband beat me + ex husband beats me + exboyfriend beat me + exboyfriend beats me + exhusband beat me + exhusband beats me + husband hit me + husband hits me + boyfriend hit me + boyfriend hits me + ex-boyfriend hit me + ex-boyfriend hits me + ex-husband hit me + ex-husband hits me + ex boyfriend hit me + ex boyfriend hits me + ex husband hit me + ex husband hits me + exboyfriend hit me + exboyfriend hits me + wife held gun to my head + wife pointed gun at me + wife points gun at me + girlfriend held gun to my head + girlfriend pointed gun at me + girlfriend points gun at me + ex-wife held gun to my head + ex-wife pointed gun at me + ex-wife points gun at me + ex-girlfriend held gun to my head + ex-girlfriend pointed gun at me + ex-girlfriend points gun at me + ex wife held gun to my head + ex wife pointed gun at me + ex wife points gun at me + ex girlfriend held gun to my head + ex girlfriend pointed gun at me + ex girlfriend points gun at me + exwife held gun to my head + exwife pointed gun at me + exwife points gun at me + exgirlfriend held gun to my head + exgirlfriend pointed gun at me + exgirlfriend points gun at me + wife slam me + wife slams me + wife slammed me + girlfriend slam me + girlfriend slams me + girlfriend slammed me + ex-wife slam me + ex-wife slams me + ex-wife slammed me + ex-girlfriend slam me + ex-girlfriend slams me + ex-girlfriend slammed me + ex wife slam me + ex wife slams me + ex wife slammed me + ex girlfriend slam me + ex girlfriend slams me + ex girlfriend slammed me + exwife slam me + exwife slams me + exwife slammed me + exgirlfriend slam me + exgirlfriend slams me + exgirlfriend slammed me + wife strangle me + wife strangles me + wife strangled me + girlfriend strangle me + girlfriend strangles me + girlfriend strangled me + ex-wife strangle me + ex-wife strangles me + ex-wife strangled me + ex-girlfriend strangle me + ex-girlfriend strangles me + ex-girlfriend strangled me + ex wife strangle me + ex wife strangles me + ex wife strangled me + ex girlfriend strangle me + ex girlfriend strangles me + ex girlfriend strangled me + exwife strangle me + exwife strangles me + exwife strangled me + exgirlfriend strangle me + exgirlfriend strangles me + exgirlfriend strangled me + wife punch me + wife punches me + wife punched me + girlfriend punch me + girlfriend punches me + girlfriend punched me + ex-wife punch me + ex-wife punches me + ex-wife punched me + ex-girlfriend punch me + ex-girlfriend punches me + ex-girlfriend punched me + ex wife punch me + ex wife punches me + ex wife punched me + ex girlfriend punch me + ex girlfriend punches me + ex girlfriend punched me + exwife punch me + exwife punches me + exwife punched me + exgirlfriend punch me + exgirlfriend punches me + exgirlfriend punched me + wife slap me + wife slaps me + wife slapped me + girlfriend slap me + girlfriend slaps me + girlfriend slapped me + ex-wife slap me + ex-wife slaps me + ex-wife slapped me + ex-girlfriend slap me + ex-girlfriend slaps me + ex-girlfriend slapped me + ex wife slap me + ex wife slaps me + ex wife slapped me + ex girlfriend slap me + ex girlfriend slaps me + ex girlfriend slapped me + exwife slap me + exwife slaps me + exwife slapped me + exgirlfriend slap me + exgirlfriend slaps me + exgirlfriend slapped me + wife beat me + wife beats me + girlfriend beat me + girlfriend beats me + ex-wife beat me + ex-wife beats me + ex-girlfriend beat me + ex-girlfriend beats me + ex wife beat me + ex wife beats me + ex girlfriend beat me + ex girlfriend beats me + exwife beat me + exwife beats me + exgirlfriend beat me + exgirlfriend beats me + wife hit me + wife hits me + girlfriend hit me + girlfriend hits me + ex-wife hit me + ex-wife hits me + ex-girlfriend hit me + ex-girlfriend hits me + ex wife hit me + ex wife hits me + ex girlfriend hit me + ex girlfriend hits me + exwife hit me + exwife hits me + exgirlfriend hit me + exgirlfriend hits me + partner held gun to my head + partner pointed gun at me + partner points gun at me + ex-partner held gun to my head + ex-partner pointed gun at me + ex-partner points gun at me + expartner held gun to my head + expartner pointed gun at me + expartner points gun at me + ex partner held gun to my head + ex partner pointed gun at me + ex partner points gun at me + ex held gun to my head + ex pointed gun at me + ex points gun at me + spouse held gun to my head + spouse pointed gun at me + spouse points gun at me + ex-spouse held gun to my head + ex-spouse pointed gun at me + ex-spouse points gun at me + exspouse held gun to my head + exspouse pointed gun at me + exspouse points gun at me + ex spouse held gun to my head + ex spouse pointed gun at me + ex spouse points gun at me + significant other held gun to my head + significant other pointed gun at me + significant other points gun at me + partner slam me + partner slams me + partner slammed me + ex-partner slam me + ex-partner slams me + ex-partner slammed me + expartner slam me + expartner slams me + expartner slammed me + ex partner slam me + ex partner slams me + ex partner slammed me + ex slam me + ex slams me + ex slammed me + spouse slam me + spouse slams me + spouse slammed me + ex-spouse slam me + ex-spouse slams me + ex-spouse slammed me + exspouse slam me + exspouse slams me + exspouse slammed me + ex spouse slam me + ex spouse slams me + ex spouse slammed me + significant other slam me + significant other slams me + significant other slammed me + partner strangle me + partner strangles me + partner strangled me + ex-partner strangle me + ex-partner strangles me + ex-partner strangled me + expartner strangle me + expartner strangles me + expartner strangled me + ex partner strangle me + ex partner strangles me + ex partner strangled me + ex strangle me + ex strangles me + ex strangled me + spouse strangle me + spouse strangles me + spouse strangled me + ex-spouse strangle me + ex-spouse strangles me + ex-spouse strangled me + exspouse strangle me + exspouse strangles me + exspouse strangled me + ex spouse strangle me + ex spouse strangles me + ex spouse strangled me + significant other strangle me + significant other strangles me + significant other strangled me + partner punch me + partner punches me + partner punched me + ex-partner punch me + ex-partner punches me + ex-partner punched me + expartner punch me + expartner punches me + expartner punched me + ex partner punch me + ex partner punches me + ex partner punched me + ex punch me + ex punches me + ex punched me + spouse punch me + spouse punches me + spouse punched me + ex-spouse punch me + ex-spouse punches me + ex-spouse punched me + exspouse punch me + exspouse punches me + exspouse punched me + ex spouse punch me + ex spouse punches me + ex spouse punched me + significant other punch me + significant other punches me + significant other punched me + partner slap me + partner slaps me + partner slapped me + ex-partner slap me + ex-partner slaps me + ex-partner slapped me + expartner slap me + expartner slaps me + expartner slapped me + ex partner slap me + ex partner slaps me + ex partner slapped me + ex slap me + ex slaps me + ex slapped me + spouse slap me + spouse slaps me + spouse slapped me + ex-spouse slap me + ex-spouse slaps me + ex-spouse slapped me + exspouse slap me + exspouse slaps me + exspouse slapped me + ex spouse slap me + ex spouse slaps me + ex spouse slapped me + significant other slap me + significant other slaps me + significant other slapped me + partner beat me + partner beats me + ex-partner beat me + ex-partner beats me + expartner beat me + expartner beats me + ex partner beat me + ex partner beats me + ex beat me + ex beats me + spouse beat me + spouse beats me + ex-spouse beat me + ex-spouse beats me + exspouse beat me + exspouse beats me + ex spouse beat me + ex spouse beats me + significant other beat me + significant other beats me + partner hit me + partner hits me + ex-partner hit me + ex-partner hits me + expartner hit me + expartner hits me + ex partner hit me + ex partner hits me + ex hit me + ex hits me + spouse hit me + spouse hits me + ex-spouse hit me + ex-spouse hits me + exspouse hit me + exspouse hits me + ex spouse hit me + ex spouse hits me + significant other hit me + significant other hits me

**First-hand account intimate partner violence search terms**

**Emotional Abuse**

husband bully me + husband bullies me + husband bullied me + husband bullying me + boyfriend bully me + boyfriend bullies me + boyfriend bullied me + boyfriend bullying me + ex-boyfriend bully me + ex-boyfriend bullies me + ex-boyfriend bullied me + ex-boyfriend bullying me + ex-husband bully me + ex-husband bullies me + ex-husband bullied me + ex-husband bullying me + ex boyfriend bully me + ex boyfriend bullies me + ex boyfriend bullied me + ex boyfriend bullying me + ex husband bully me + ex husband bullies me + ex husband bullied me + ex husband bullying me + exboyfriend bully me + exboyfriend bullies me + exboyfriend bullied me + exboyfriend bullying me + exhusband bully me + exhusband bullies me + exhusband bullied me + exhusband bullying me + husband terrifies me + husband frightens me + boyfriend terrifies me + boyfriend frightens me + ex-boyfriend terrifies me + ex-boyfriend frightens me + ex-husband terrifies me + ex-husband frightens me + ex boyfriend terrifies me + ex boyfriend frightens me + ex husband terrifies me + ex husband frightens me + exboyfriend terrifies me + exboyfriend frightens me + exhusband terrifies me + exhusband frightens me + husband threatens my family + husband threatened my family + husband threaten my family + boyfriend threatens my family + boyfriend threatened my family + boyfriend threaten my family + ex-boyfriend threatens my family + ex-boyfriend threatened my family + ex-boyfriend threaten my family + ex-husband threatens my family + ex-husband threatened my family + ex-husband threaten my family + ex boyfriend threatens my family + ex boyfriend threatened my family + ex boyfriend threaten my family + ex husband threatens my family + ex husband threatened my family + ex husband threaten my family + exboyfriend threatens my family + exboyfriend threatened my family + exboyfriend threaten my family + exhusband threatens my family + exhusband threatened my family + exhusband threaten my family + husband put me down me + husband puts me down me + husband putting me down me + boyfriend put me down me + boyfriend puts me down me + boyfriend putting me down me + ex-boyfriend put me down me + ex-boyfriend puts me down me + ex-boyfriend putting me down me + ex-husband put me down me + ex-husband puts me down me + ex-husband putting me down me + ex boyfriend put me down me + ex boyfriend puts me down me + ex boyfriend putting me down me + ex husband put me down me + ex husband puts me down me + ex husband putting me down me + exboyfriend put me down me + exboyfriend puts me down me + exboyfriend putting me down me + exhusband put me down me + exhusband puts me down me + exhusband putting me down me + husband swear at me + husband swears at me + husband swore at me + boyfriend swear at me + boyfriend swears at me + boyfriend swore at me + ex-boyfriend swear at me + ex-boyfriend swears at me + ex-boyfriend swore at me + ex-husband swear at me + ex-husband swears at me + ex-husband swore at me + ex boyfriend swear at me + ex boyfriend swears at me + ex boyfriend swore at me + ex husband swear at me + ex husband swears at me + ex husband swore at me + exboyfriend swear at me + exboyfriend swears at me + exboyfriend swore at me + exhusband swear at me + exhusband swears at me + exhusband swore at me + husband insult me + husband insults me + husband insulted me + boyfriend insult me + boyfriend insults me + boyfriend insulted me + ex-boyfriend insult me + ex-boyfriend insults me + ex-boyfriend insulted me + ex-husband insult me + ex-husband insults me + ex-husband insulted me + ex boyfriend insult me + ex boyfriend insults me + ex boyfriend insulted me + ex husband insult me + ex husband insults me + ex husband insulted me + exboyfriend insult me + exboyfriend insults me + exboyfriend insulted me + exhusband insult me + exhusband insults me + exhusband insulted me + husband yell at me + husband yells at me + husband yelled at me + boyfriend yell at me + boyfriend yells at me + boyfriend yelled at me + ex-boyfriend yell at me + ex-boyfriend yells at me + ex-boyfriend yelled at me + ex-husband yell at me + ex-husband yells at me + ex-husband yelled at me + ex boyfriend yell at me + ex boyfriend yells at me + ex boyfriend yelled at me + ex husband yell at me + ex husband yells at me + ex husband yelled at me + exboyfriend yell at me + exboyfriend yells at me + exboyfriend yelled at me + exhusband yell at me + exhusband yells at me + exhusband yelled at me + husband scared me + husband scares me + husband scare me + scared of my husband + boyfriend scared me + boyfriend scares me + boyfriend scare me + scared of my boyfriend + ex-boyfriend scared me + ex-boyfriend scares me + ex-boyfriend scare me + scared of my ex-boyfriend + ex-husband scared me + ex-husband scares me + ex-husband scare me + scared of my ex-husband + ex boyfriend scared me + ex boyfriend scares me + ex boyfriend scare me + scared of my ex boyfriend + ex husband scared me + ex husband scares me + ex husband scare me + scared of my ex husband + exboyfriend scared me + exboyfriend scares me + exboyfriend scare me + scared of my exboyfriend + exhusband scared me + exhusband scares me + exhusband scare me + scared of my exhusband + afraid of my husband + husband makes me afraid + afraid of my boyfriend + boyfriend makes me afraid + afraid of my ex-boyfriend + ex-boyfriend makes me afraid + afraid of my ex-husband + ex-husband makes me afraid + afraid of my ex boyfriend + ex boyfriend makes me afraid + afraid of my ex husband + ex husband makes me afraid + afraid of my exboyfriend + exboyfriend makes me afraid + afraid of my exhusband + exhusband makes me afraid + husband control me + husband controlled me + husband controls me + husband controlling me + boyfriend control me + boyfriend controlled me + boyfriend controls me + boyfriend controlling me + ex-boyfriend control me + ex-boyfriend controlled me + ex-boyfriend controls me + ex-boyfriend controlling me + ex-husband control me + ex-husband controlled me + ex-husband controls me + ex-husband controlling me + ex boyfriend control me + ex boyfriend controlled me + ex boyfriend controls me + ex boyfriend controlling me + ex husband control me + ex husband controlled me + ex husband controls me + ex husband controlling me + exboyfriend control me + exboyfriend controlled me + exboyfriend controls me + exboyfriend controlling me + exhusband control me + exhusband controlled me + exhusband controls me + exhusband controlling me + husband threaten me + husband threatened me + husband threatens me + husband threatening me + boyfriend threaten me + boyfriend threatened me + boyfriend threatens me + boyfriend threatening me + ex-boyfriend threaten me + ex-boyfriend threatened me + ex-boyfriend threatens me + ex-boyfriend threatening me + ex-husband threaten me + ex-husband threatened me + ex-husband threatens me + ex-husband threatening me + ex boyfriend threaten me + ex boyfriend threatened me + ex boyfriend threatens me + ex boyfriend threatening me + ex husband threaten me + ex husband threatened me + ex husband threatens me + ex husband threatening me + exboyfriend threaten me + exboyfriend threatened me + exboyfriend threatens me + exboyfriend threatening me + exhusband threaten me + exhusband threatened me + exhusband threatens me + exhusband threatening me + wife bully me + wife bullies me + wife bullied me + wife bullying me + girlfriend bully me + girlfriend bullies me + girlfriend bullied me + girlfriend bullying me + ex-wife bully me + ex-wife bullies me + ex-wife bullied me + ex-wife bullying me + ex-girlfriend bully me + ex-girlfriend bullies me + ex-girlfriend bullied me + ex-girlfriend bullying me + ex wife bully me + ex wife bullies me + ex wife bullied me + ex wife bullying me + ex girlfriend bully me + ex girlfriend bullies me + ex girlfriend bullied me + ex girlfriend bullying me + exwife bully me + exwife bullies me + exwife bullied me + exwife bullying me + exgirlfriend bully me + exgirlfriend bullies me + exgirlfriend bullied me + exgirlfriend bullying me + wife terrifies me + wife frightens me + girlfriend terrifies me + girlfriend frightens me + ex-wife terrifies me + ex-wife frightens me + ex-girlfriend terrifies me + ex-girlfriend frightens me + ex wife terrifies me + ex wife frightens me + ex girlfriend terrifies me + ex girlfriend frightens me + exwife terrifies me + exwife frightens me + exgirlfriend terrifies me + exgirlfriend frightens me + wife threatens my family + wife threatened my family + wife threaten my family + girlfriend threatens my family + girlfriend threatened my family + girlfriend threaten my family + ex-wife threatens my family + ex-wife threatened my family + ex-wife threaten my family + ex-girlfriend threatens my family + ex-girlfriend threatened my family + ex-girlfriend threaten my family + ex wife threatens my family + ex wife threatened my family + ex wife threaten my family + ex girlfriend threatens my family + ex girlfriend threatened my family + ex girlfriend threaten my family + exwife threatens my family + exwife threatened my family + exwife threaten my family + exgirlfriend threatens my family + exgirlfriend threatened my family + exgirlfriend threaten my family + wife put me down me + wife puts me down me + wife putting me down me + girlfriend put me down me + girlfriend puts me down me + girlfriend putting me down me + ex-wife put me down me + ex-wife puts me down me + ex-wife putting me down me + ex-girlfriend put me down me + ex-girlfriend puts me down me + ex-girlfriend putting me down me + ex wife put me down me + ex wife puts me down me + ex wife putting me down me + ex girlfriend put me down me + ex girlfriend puts me down me + ex girlfriend putting me down me + exwife put me down me + exwife puts me down me + exwife putting me down me + exgirlfriend put me down me + exgirlfriend puts me down me + exgirlfriend putting me down me + wife swear at me + wife swears at me + wife swore at me + girlfriend swear at me + girlfriend swears at me + girlfriend swore at me + ex-wife swear at me + ex-wife swears at me + ex-wife swore at me + ex-girlfriend swear at me + ex-girlfriend swears at me + ex-girlfriend swore at me + ex wife swear at me + ex wife swears at me + ex wife swore at me + ex girlfriend swear at me + ex girlfriend swears at me + ex girlfriend swore at me + exwife swear at me + exwife swears at me + exwife swore at me + exgirlfriend swear at me + exgirlfriend swears at me + exgirlfriend swore at me + wife insult me + wife insults me + wife insulted me + girlfriend insult me + girlfriend insults me + girlfriend insulted me + ex-wife insult me + ex-wife insults me + ex-wife insulted me + ex-girlfriend insult me + ex-girlfriend insults me + ex-girlfriend insulted me + ex wife insult me + ex wife insults me + ex wife insulted me + ex girlfriend insult me + ex girlfriend insults me + ex girlfriend insulted me + exwife insult me + exwife insults me + exwife insulted me + exgirlfriend insult me + exgirlfriend insults me + exgirlfriend insulted me + wife yell at me + wife yells at me + wife yelled at me + girlfriend yell at me + girlfriend yells at me + girlfriend yelled at me + ex-wife yell at me + ex-wife yells at me + ex-wife yelled at me + ex-girlfriend yell at me + ex-girlfriend yells at me + ex-girlfriend yelled at me + ex wife yell at me + ex wife yells at me + ex wife yelled at me + ex girlfriend yell at me + ex girlfriend yells at me + ex girlfriend yelled at me + exwife yell at me + exwife yells at me + exwife yelled at me + exgirlfriend yell at me + exgirlfriend yells at me + exgirlfriend yelled at me + wife scared me + wife scares me + wife scare me + scared of my wife + girlfriend scared me + girlfriend scares me + girlfriend scare me + scared of my girlfriend + ex-wife scared me + ex-wife scares me + ex-wife scare me + scared of my ex-wife + ex-girlfriend scared me + ex-girlfriend scares me + ex-girlfriend scare me + scared of my ex-girlfriend + ex wife scared me + ex wife scares me + ex wife scare me + scared of my ex wife + ex girlfriend scared me + ex girlfriend scares me + ex girlfriend scare me + scared of my ex girlfriend + exwife scared me + exwife scares me + exwife scare me + scared of my exwife + exgirlfriend scared me + exgirlfriend scares me + exgirlfriend scare me + scared of my exgirlfriend + afraid of my wife + wife makes me afraid + afraid of my girlfriend + girlfriend makes me afraid + afraid of my ex-wife + ex-wife makes me afraid + afraid of my ex-girlfriend + ex-girlfriend makes me afraid + afraid of my ex wife + ex wife makes me afraid + afraid of my ex girlfriend + ex girlfriend makes me afraid + afraid of my exwife + exwife makes me afraid + afraid of my exgirlfriend + exgirlfriend makes me afraid + wife control me + wife controlled me + wife controls me + wife controlling me + girlfriend control me + girlfriend controlled me + girlfriend controls me + girlfriend controlling me + ex-wife control me + ex-wife controlled me + ex-wife controls me + ex-wife controlling me + ex-girlfriend control me + ex-girlfriend controlled me + ex-girlfriend controls me + ex-girlfriend controlling me + ex wife control me + ex wife controlled me + ex wife controls me + ex wife controlling me + ex girlfriend control me + ex girlfriend controlled me + ex girlfriend controls me + ex girlfriend controlling me + exwife control me + exwife controlled me + exwife controls me + exwife controlling me + exgirlfriend control me + exgirlfriend controlled me + exgirlfriend controls me + exgirlfriend controlling me + wife threaten me + wife threatened me + wife threatens me + wife threatening me + girlfriend threaten me + girlfriend threatened me + girlfriend threatens me + girlfriend threatening me + ex-wife threaten me + ex-wife threatened me + ex-wife threatens me + ex-wife threatening me + ex-girlfriend threaten me + ex-girlfriend threatened me + ex-girlfriend threatens me + ex-girlfriend threatening me + ex wife threaten me + ex wife threatened me + ex wife threatens me + ex wife threatening me + ex girlfriend threaten me + ex girlfriend threatened me + ex girlfriend threatens me + ex girlfriend threatening me + exwife threaten me + exwife threatened me + exwife threatens me + exwife threatening me + exgirlfriend threaten me + exgirlfriend threatened me + exgirlfriend threatens me + exgirlfriend threatening me + partner bully me + partner bullies me + partner bullied me + partner bullying me + ex-partner bully me + ex-partner bullies me + ex-partner bullied me + ex-partner bullying me + expartner bully me + expartner bullies me + expartner bullied me + expartner bullying me + ex partner bully me + ex partner bullies me + ex partner bullied me + ex partner bullying me + ex bully me + ex bullies me + ex bullied me + ex bullying me + spouse bully me + spouse bullies me + spouse bullied me + spouse bullying me + ex-spouse bully me + ex-spouse bullies me + ex-spouse bullied me + ex-spouse bullying me + exspouse bully me + exspouse bullies me + exspouse bullied me + exspouse bullying me + ex spouse bully me + ex spouse bullies me + ex spouse bullied me + ex spouse bullying me + significant other bully me + significant other bullies me + significant other bullied me + significant other bullying me + partner terrifies me + partner frightens me + ex-partner terrifies me + ex-partner frightens me + expartner terrifies me + expartner frightens me + ex partner terrifies me + ex partner frightens me + ex terrifies me + ex frightens me + spouse terrifies me + spouse frightens me + ex-spouse terrifies me + ex-spouse frightens me + exspouse terrifies me + exspouse frightens me + ex spouse terrifies me + ex spouse frightens me + significant other terrifies me + significant other frightens me + partner threatens my family + partner threatened my family + partner threaten my family + ex-partner threatens my family + ex-partner threatened my family + ex-partner threaten my family + expartner threatens my family + expartner threatened my family + expartner threaten my family + ex partner threatens my family + ex partner threatened my family + ex partner threaten my family + ex threatens my family + ex threatened my family + ex threaten my family + spouse threatens my family + spouse threatened my family + spouse threaten my family + ex-spouse threatens my family + ex-spouse threatened my family + ex-spouse threaten my family + exspouse threatens my family + exspouse threatened my family + exspouse threaten my family + ex spouse threatens my family + ex spouse threatened my family + ex spouse threaten my family + significant other threatens my family + significant other threatened my family + significant other threaten my family + partner put me down me + partner puts me down me + partner putting me down me + ex-partner put me down me + ex-partner puts me down me + ex-partner putting me down me + expartner put me down me + expartner puts me down me + expartner putting me down me + ex partner put me down me + ex partner puts me down me + ex partner putting me down me + ex put me down me + ex puts me down me + ex putting me down me + spouse put me down me + spouse puts me down me + spouse putting me down me + ex-spouse put me down me + ex-spouse puts me down me + ex-spouse putting me down me + exspouse put me down me + exspouse puts me down me + exspouse putting me down me + ex spouse put me down me + ex spouse puts me down me + ex spouse putting me down me + significant other put me down me + significant other puts me down me + significant other putting me down me + partner swear at me + partner swears at me + partner swore at me + ex-partner swear at me + ex-partner swears at me + ex-partner swore at me + expartner swear at me + expartner swears at me + expartner swore at me + ex partner swear at me + ex partner swears at me + ex partner swore at me + ex swear at me + ex swears at me + ex swore at me + spouse swear at me + spouse swears at me + spouse swore at me + ex-spouse swear at me + ex-spouse swears at me + ex-spouse swore at me + exspouse swear at me + exspouse swears at me + exspouse swore at me + ex spouse swear at me + ex spouse swears at me + ex spouse swore at me + significant other swear at me + significant other swears at me + significant other swore at me + partner insult me + partner insults me + partner insulted me + ex-partner insult me + ex-partner insults me + ex-partner insulted me + expartner insult me + expartner insults me + expartner insulted me + ex partner insult me + ex partner insults me + ex partner insulted me + ex insult me + ex insults me + ex insulted me + spouse insult me + spouse insults me + spouse insulted me + ex-spouse insult me + ex-spouse insults me + ex-spouse insulted me + exspouse insult me + exspouse insults me + exspouse insulted me + ex spouse insult me + ex spouse insults me + ex spouse insulted me + significant other insult me + significant other insults me + significant other insulted me + partner yell at me + partner yells at me + partner yelled at me + ex-partner yell at me + ex-partner yells at me + ex-partner yelled at me + expartner yell at me + expartner yells at me + expartner yelled at me + ex partner yell at me + ex partner yells at me + ex partner yelled at me + ex yell at me + ex yells at me + ex yelled at me + spouse yell at me + spouse yells at me + spouse yelled at me + ex-spouse yell at me + ex-spouse yells at me + ex-spouse yelled at me + exspouse yell at me + exspouse yells at me + exspouse yelled at me + ex spouse yell at me + ex spouse yells at me + ex spouse yelled at me + significant other yell at me + significant other yells at me + significant other yelled at me + partner scared me + partner scares me + partner scare me + scared of my partner + ex-partner scared me + ex-partner scares me + ex-partner scare me + scared of my ex-partner + expartner scared me + expartner scares me + expartner scare me + scared of my expartner + ex partner scared me + ex partner scares me + ex partner scare me + scared of my ex partner + ex scared me + ex scares me + ex scare me + scared of my ex + spouse scared me + spouse scares me + spouse scare me + scared of my spouse + ex-spouse scared me + ex-spouse scares me + ex-spouse scare me + scared of my ex-spouse + exspouse scared me + exspouse scares me + exspouse scare me + scared of my exspouse + ex spouse scared me + ex spouse scares me + ex spouse scare me + scared of my ex spouse + significant other scared me + significant other scares me + significant other scare me + scared of my significant other + afraid of my partner + partner makes me afraid + afraid of my ex-partner + ex-partner makes me afraid + afraid of my expartner + expartner makes me afraid + afraid of my ex partner + ex partner makes me afraid + afraid of my ex + ex makes me afraid + afraid of my spouse + spouse makes me afraid + afraid of my ex-spouse + ex-spouse makes me afraid + afraid of my exspouse + exspouse makes me afraid + afraid of my ex spouse + ex spouse makes me afraid + afraid of my significant other + significant other makes me afraid + partner control me + partner controlled me + partner controls me + partner controlling me + ex-partner control me + ex-partner controlled me + ex-partner controls me + ex-partner controlling me + expartner control me + expartner controlled me + expartner controls me + expartner controlling me + ex partner control me + ex partner controlled me + ex partner controls me + ex partner controlling me + ex control me + ex controlled me + ex controls me + ex controlling me + spouse control me + spouse controlled me + spouse controls me + spouse controlling me + ex-spouse control me + ex-spouse controlled me + ex-spouse controls me + ex-spouse controlling me + exspouse control me + exspouse controlled me + exspouse controls me + exspouse controlling me + ex spouse control me + ex spouse controlled me + ex spouse controls me + ex spouse controlling me + significant other control me + significant other controlled me + significant other controls me + significant other controlling me + partner threaten me + partner threatened me + partner threatens me + partner threatening me + ex-partner threaten me + ex-partner threatened me + ex-partner threatens me + ex-partner threatening me + expartner threaten me + expartner threatened me + expartner threatens me + expartner threatening me + ex partner threaten me + ex partner threatened me + ex partner threatens me + ex partner threatening me + ex threaten me + ex threatened me + ex threatens me + ex threatening me + spouse threaten me + spouse threatened me + spouse threatens me + spouse threatening me + ex-spouse threaten me + ex-spouse threatened me + ex-spouse threatens me + ex-spouse threatening me + exspouse threaten me + exspouse threatened me + exspouse threatens me + exspouse threatening me + ex spouse threaten me + ex spouse threatened me + ex spouse threatens me + ex spouse threatening me + significant other threaten me + significant other threatened me + significant other threatens me + significant other threatening me

**First-hand account intimate partner violence search terms**

**Sexual Abuse**

husband force me have sex + husband forced me have sex + husband forcing me have sex + husband forces me have sex + boyfriend force me have sex + boyfriend forced me have sex + boyfriend forcing me have sex + boyfriend forces me have sex + ex-boyfriend force me have sex + ex-boyfriend forced me have sex + ex-boyfriend forcing me have sex + ex-boyfriend forces me have sex + ex-husband force me have sex + ex-husband forced me have sex + ex-husband forcing me have sex + ex-husband forces me have sex + ex boyfriend force me have sex + ex boyfriend forced me have sex + ex boyfriend forcing me have sex + ex boyfriend forces me have sex + ex husband force me have sex + ex husband forced me have sex + ex husband forcing me have sex + ex husband forces me have sex + exboyfriend force me have sex + exboyfriend forced me have sex + exboyfriend forcing me have sex + exboyfriend forces me have sex + exhusband force me have sex + exhusband forced me have sex + exhusband forcing me have sex + exhusband forces me have sex + husband pressure me have sex + husband pressured me have sex + husband pressuring me have sex + husband pressures me have sex + boyfriend pressure me have sex + boyfriend pressured me have sex + boyfriend pressuring me have sex + boyfriend pressures me have sex + ex-boyfriend pressure me have sex + ex-boyfriend pressured me have sex + ex-boyfriend pressuring me have sex + ex-boyfriend pressures me have sex + ex-husband pressure me have sex + ex-husband pressured me have sex + ex-husband pressuring me have sex + ex-husband pressures me have sex + ex boyfriend pressure me have sex + ex boyfriend pressured me have sex + ex boyfriend pressuring me have sex + ex boyfriend pressures me have sex + ex husband pressure me have sex + ex husband pressured me have sex + ex husband pressuring me have sex + ex husband pressures me have sex + exboyfriend pressure me have sex + exboyfriend pressured me have sex + exboyfriend pressuring me have sex + exboyfriend pressures me have sex + exhusband pressure me have sex + exhusband pressured me have sex + exhusband pressuring me have sex + exhusband pressures me have sex + husband rape me + husband raped me + husband raping me + husband rapes me + boyfriend rape me + boyfriend raped me + boyfriend raping me + boyfriend rapes me + ex-boyfriend rape me + ex-boyfriend raped me + ex-boyfriend raping me + ex-boyfriend rapes me + ex-husband rape me + ex-husband raped me + ex-husband raping me + ex-husband rapes me + ex boyfriend rape me + ex boyfriend raped me + ex boyfriend raping me + ex boyfriend rapes me + ex husband rape me + ex husband raped me + ex husband raping me + ex husband rapes me + exboyfriend rape me + exboyfriend raped me + exboyfriend raping me + exboyfriend rapes me + exhusband rape me + exhusband raped me + exhusband raping me + exhusband rapes me + husband sexually abused me + husband sexually abusing me + husband sexually abuses me + boyfriend sexually abused me + boyfriend sexually abusing me + boyfriend sexually abuses me + ex-boyfriend sexually abused me + ex-boyfriend sexually abusing me + ex-boyfriend sexually abuses me + ex-husband sexually abused me + ex-husband sexually abusing me + ex-husband sexually abuses me + ex boyfriend sexually abused me + ex boyfriend sexually abusing me + ex boyfriend sexually abuses me + ex husband sexually abused me + ex husband sexually abusing me + ex husband sexually abuses me + exboyfriend sexually abused me + exboyfriend sexually abusing me + exboyfriend sexually abuses me + exhusband sexually abused me + exhusband sexually abusing me + exhusband sexually abuses me + husband made me have sex + husband makes me have sex + husband make me have sex + boyfriend made me have sex + boyfriend makes me have sex + boyfriend make me have sex + ex-boyfriend made me have sex + ex-boyfriend makes me have sex + ex-boyfriend make me have sex + ex-husband made me have sex + ex-husband makes me have sex + ex-husband make me have sex + ex boyfriend made me have sex + ex boyfriend makes me have sex + ex boyfriend make me have sex + ex husband made me have sex + ex husband makes me have sex + ex husband make me have sex + exboyfriend made me have sex + exboyfriend makes me have sex + exboyfriend make me have sex + exhusband made me have sex + exhusband makes me have sex + exhusband make me have sex + wife force me have sex + wife forced me have sex + wife forcing me have sex + wife forces me have sex + girlfriend force me have sex + girlfriend forced me have sex + girlfriend forcing me have sex + girlfriend forces me have sex + ex-wife force me have sex + ex-wife forced me have sex + ex-wife forcing me have sex + ex-wife forces me have sex + ex-girlfriend force me have sex + ex-girlfriend forced me have sex + ex-girlfriend forcing me have sex + ex-girlfriend forces me have sex + ex wife force me have sex + ex wife forced me have sex + ex wife forcing me have sex + ex wife forces me have sex + ex girlfriend force me have sex + ex girlfriend forced me have sex + ex girlfriend forcing me have sex + ex girlfriend forces me have sex + exwife force me have sex + exwife forced me have sex + exwife forcing me have sex + exwife forces me have sex + exgirlfriend force me have sex + exgirlfriend forced me have sex + exgirlfriend forcing me have sex + exgirlfriend forces me have sex + wife pressure me have sex + wife pressured me have sex + wife pressuring me have sex + wife pressures me have sex + girlfriend pressure me have sex + girlfriend pressured me have sex + girlfriend pressuring me have sex + girlfriend pressures me have sex + ex-wife pressure me have sex + ex-wife pressured me have sex + ex-wife pressuring me have sex + ex-wife pressures me have sex + ex-girlfriend pressure me have sex + ex-girlfriend pressured me have sex + ex-girlfriend pressuring me have sex + ex-girlfriend pressures me have sex + ex wife pressure me have sex + ex wife pressured me have sex + ex wife pressuring me have sex + ex wife pressures me have sex + ex girlfriend pressure me have sex + ex girlfriend pressured me have sex + ex girlfriend pressuring me have sex + ex girlfriend pressures me have sex + exwife pressure me have sex + exwife pressured me have sex + exwife pressuring me have sex + exwife pressures me have sex + exgirlfriend pressure me have sex + exgirlfriend pressured me have sex + exgirlfriend pressuring me have sex + exgirlfriend pressures me have sex + wife rape me + wife raped me + wife raping me + wife rapes me + girlfriend rape me + girlfriend raped me + girlfriend raping me + girlfriend rapes me + ex-wife rape me + ex-wife raped me + ex-wife raping me + ex-wife rapes me + ex-girlfriend rape me + ex-girlfriend raped me + ex-girlfriend raping me + ex-girlfriend rapes me + ex wife rape me + ex wife raped me + ex wife raping me + ex wife rapes me + ex girlfriend rape me + ex girlfriend raped me + ex girlfriend raping me + ex girlfriend rapes me + exwife rape me + exwife raped me + exwife raping me + exwife rapes me + exgirlfriend rape me + exgirlfriend raped me + exgirlfriend raping me + exgirlfriend rapes me + wife sexually abused me + wife sexually abusing me + wife sexually abuses me + girlfriend sexually abused me + girlfriend sexually abusing me + girlfriend sexually abuses me + ex-wife sexually abused me + ex-wife sexually abusing me + ex-wife sexually abuses me + ex-girlfriend sexually abused me + ex-girlfriend sexually abusing me + ex-girlfriend sexually abuses me + ex wife sexually abused me + ex wife sexually abusing me + ex wife sexually abuses me + ex girlfriend sexually abused me + ex girlfriend sexually abusing me + ex girlfriend sexually abuses me + exwife sexually abused me + exwife sexually abusing me + exwife sexually abuses me + exgirlfriend sexually abused me + exgirlfriend sexually abusing me + exgirlfriend sexually abuses me + wife made me have sex + wife makes me have sex + wife make me have sex + girlfriend made me have sex + girlfriend makes me have sex + girlfriend make me have sex + ex-wife made me have sex + ex-wife makes me have sex + ex-wife make me have sex + ex-girlfriend made me have sex + ex-girlfriend makes me have sex + ex-girlfriend make me have sex + ex wife made me have sex + ex wife makes me have sex + ex wife make me have sex + ex girlfriend made me have sex + ex girlfriend makes me have sex + ex girlfriend make me have sex + exwife made me have sex + exwife makes me have sex + exwife make me have sex + exgirlfriend made me have sex + exgirlfriend makes me have sex + exgirlfriend make me have sex + partner force me have sex + partner forced me have sex + partner forcing me have sex + partner forces me have sex + ex-partner force me have sex + ex-partner forced me have sex + ex-partner forcing me have sex + ex-partner forces me have sex + expartner force me have sex + expartner forced me have sex + expartner forcing me have sex + expartner forces me have sex + ex partner force me have sex + ex partner forced me have sex + ex partner forcing me have sex + ex partner forces me have sex + ex force me have sex + ex forced me have sex + ex forcing me have sex + ex forces me have sex + spouse force me have sex + spouse forced me have sex + spouse forcing me have sex + spouse forces me have sex + ex-spouse force me have sex + ex-spouse forced me have sex + ex-spouse forcing me have sex + ex-spouse forces me have sex + exspouse force me have sex + exspouse forced me have sex + exspouse forcing me have sex + exspouse forces me have sex + ex spouse force me have sex + ex spouse forced me have sex + ex spouse forcing me have sex + ex spouse forces me have sex + significant other force me have sex + significant other forced me have sex + significant other forcing me have sex + significant other forces me have sex + partner pressure me have sex + partner pressured me have sex + partner pressuring me have sex + partner pressures me have sex + ex-partner pressure me have sex + ex-partner pressured me have sex + ex-partner pressuring me have sex + ex-partner pressures me have sex + expartner pressure me have sex + expartner pressured me have sex + expartner pressuring me have sex + expartner pressures me have sex + ex partner pressure me have sex + ex partner pressured me have sex + ex partner pressuring me have sex + ex partner pressures me have sex + ex pressure me have sex + ex pressured me have sex + ex pressuring me have sex + ex pressures me have sex + spouse pressure me have sex + spouse pressured me have sex + spouse pressuring me have sex + spouse pressures me have sex + ex-spouse pressure me have sex + ex-spouse pressured me have sex + ex-spouse pressuring me have sex + ex-spouse pressures me have sex + exspouse pressure me have sex + exspouse pressured me have sex + exspouse pressuring me have sex + exspouse pressures me have sex + ex spouse pressure me have sex + ex spouse pressured me have sex + ex spouse pressuring me have sex + ex spouse pressures me have sex + significant other pressure me have sex + significant other pressured me have sex + significant other pressuring me have sex + significant other pressures me have sex + partner rape me + partner raped me + partner raping me + partner rapes me + ex-partner rape me + ex-partner raped me + ex-partner raping me + ex-partner rapes me + expartner rape me + expartner raped me + expartner raping me + expartner rapes me + ex partner rape me + ex partner raped me + ex partner raping me + ex partner rapes me + ex rape me + ex raped me + ex raping me + ex rapes me + spouse rape me + spouse raped me + spouse raping me + spouse rapes me + ex-spouse rape me + ex-spouse raped me + ex-spouse raping me + ex-spouse rapes me + exspouse rape me + exspouse raped me + exspouse raping me + exspouse rapes me + ex spouse rape me + ex spouse raped me + ex spouse raping me + ex spouse rapes me + significant other rape me + significant other raped me + significant other raping me + significant other rapes me + partner sexually abused me + partner sexually abusing me + partner sexually abuses me + ex-partner sexually abused me + ex-partner sexually abusing me + ex-partner sexually abuses me + expartner sexually abused me + expartner sexually abusing me + expartner sexually abuses me + ex partner sexually abused me + ex partner sexually abusing me + ex partner sexually abuses me + ex sexually abused me + ex sexually abusing me + ex sexually abuses me + spouse sexually abused me + spouse sexually abusing me + spouse sexually abuses me + ex-spouse sexually abused me + ex-spouse sexually abusing me + ex-spouse sexually abuses me + exspouse sexually abused me + exspouse sexually abusing me + exspouse sexually abuses me + ex spouse sexually abused me + ex spouse sexually abusing me + ex spouse sexually abuses me + significant other sexually abused me + significant other sexually abusing me + significant other sexually abuses me + partner made me have sex + partner makes me have sex + partner make me have sex + ex-partner made me have sex + ex-partner makes me have sex + ex-partner make me have sex + expartner made me have sex + expartner makes me have sex + expartner make me have sex + ex partner made me have sex + ex partner makes me have sex + ex partner make me have sex + ex made me have sex + ex makes me have sex + ex make me have sex + spouse made me have sex + spouse makes me have sex + spouse make me have sex + ex-spouse made me have sex + ex-spouse makes me have sex + ex-spouse make me have sex + exspouse made me have sex + exspouse makes me have sex + exspouse make me have sex + ex spouse made me have sex + ex spouse makes me have sex + ex spouse make me have sex + significant other made me have sex + significant other makes me have sex + significant other make me have sex

**First-hand account intimate partner violence search terms**

**Other Searches**

husband abuses me + husband abused me + husband is abusive + boyfriend abuses me + boyfriend abused me + boyfriend is abusive + ex-boyfriend abuses me + ex-boyfriend abused me + ex-boyfriend is abusive + ex-husband abuses me + ex-husband abused me + ex-husband is abusive + ex boyfriend abuses me + ex boyfriend abused me + ex boyfriend is abusive + ex husband abuses me + ex husband abused me + ex husband is abusive + exboyfriend abuses me + exboyfriend abused me + exboyfriend is abusive + exhusband abuses me + exhusband abused me + exhusband is abusive + husband assault me + husband assaults me + husband assaulted me + husband assaulting me + boyfriend assault me + boyfriend assaults me + boyfriend assaulted me + boyfriend assaulting me + ex-boyfriend assault me + ex-boyfriend assaults me + ex-boyfriend assaulted me + ex-boyfriend assaulting me + ex-husband assault me + ex-husband assaults me + ex-husband assaulted me + ex-husband assaulting me + ex boyfriend assault me + ex boyfriend assaults me + ex boyfriend assaulted me + ex boyfriend assaulting me + ex husband assault me + ex husband assaults me + ex husband assaulted me + ex husband assaulting me + exboyfriend assault me + exboyfriend assaults me + exboyfriend assaulted me + exboyfriend assaulting me + exhusband assault me + exhusband assaults me + exhusband assaulted me + exhusband assaulting me + husband harass me + husband harasses me + husband harassed me + husband harassing me + boyfriend harass me + boyfriend harasses me + boyfriend harassed me + boyfriend harassing me + ex-boyfriend harass me + ex-boyfriend harasses me + ex-boyfriend harassed me + ex-boyfriend harassing me + ex-husband harass me + ex-husband harasses me + ex-husband harassed me + ex-husband harassing me + ex boyfriend harass me + ex boyfriend harasses me + ex boyfriend harassed me + ex boyfriend harassing me + ex husband harass me + ex husband harasses me + ex husband harassed me + ex husband harassing me + exboyfriend harass me + exboyfriend harasses me + exboyfriend harassed me + exboyfriend harassing me + exhusband harass me + exhusband harasses me + exhusband harassed me + exhusband harassing me + wife abuses me + wife abused me + wife is abusive + girlfriend abuses me + girlfriend abused me + girlfriend is abusive + ex-wife abuses me + ex-wife abused me + ex-wife is abusive + ex-girlfriend abuses me + ex-girlfriend abused me + ex-girlfriend is abusive + ex wife abuses me + ex wife abused me + ex wife is abusive + ex girlfriend abuses me + ex girlfriend abused me + ex girlfriend is abusive + exwife abuses me + exwife abused me + exwife is abusive + exgirlfriend abuses me + exgirlfriend abused me + exgirlfriend is abusive + wife assault me + wife assaults me + wife assaulted me + wife assaulting me + girlfriend assault me + girlfriend assaults me + girlfriend assaulted me + girlfriend assaulting me + ex-wife assault me + ex-wife assaults me + ex-wife assaulted me + ex-wife assaulting me + ex-girlfriend assault me + ex-girlfriend assaults me + ex-girlfriend assaulted me + ex-girlfriend assaulting me + ex wife assault me + ex wife assaults me + ex wife assaulted me + ex wife assaulting me + ex girlfriend assault me + ex girlfriend assaults me + ex girlfriend assaulted me + ex girlfriend assaulting me + exwife assault me + exwife assaults me + exwife assaulted me + exwife assaulting me + exgirlfriend assault me + exgirlfriend assaults me + exgirlfriend assaulted me + exgirlfriend assaulting me + wife harass me + wife harasses me + wife harassed me + wife harassing me + girlfriend harass me + girlfriend harasses me + girlfriend harassed me + girlfriend harassing me + ex-wife harass me + ex-wife harasses me + ex-wife harassed me + ex-wife harassing me + ex-girlfriend harass me + ex-girlfriend harasses me + ex-girlfriend harassed me + ex-girlfriend harassing me + ex wife harass me + ex wife harasses me + ex wife harassed me + ex wife harassing me + ex girlfriend harass me + ex girlfriend harasses me + ex girlfriend harassed me + ex girlfriend harassing me + exwife harass me + exwife harasses me + exwife harassed me + exwife harassing me + exgirlfriend harass me + exgirlfriend harasses me + exgirlfriend harassed me + exgirlfriend harassing me + partner abuses me + partner abused me + partner is abusive + ex-partner abuses me + ex-partner abused me + ex-partner is abusive + expartner abuses me + expartner abused me + expartner is abusive + ex partner abuses me + ex partner abused me + ex partner is abusive + ex abuses me + ex abused me + ex is abusive + spouse abuses me + spouse abused me + spouse is abusive + ex-spouse abuses me + ex-spouse abused me + ex-spouse is abusive + exspouse abuses me + exspouse abused me + exspouse is abusive + ex spouse abuses me + ex spouse abused me + ex spouse is abusive + significant other abuses me + significant other abused me + significant other is abusive + partner assault me + partner assaults me + partner assaulted me + partner assaulting me + ex-partner assault me + ex-partner assaults me + ex-partner assaulted me + ex-partner assaulting me + expartner assault me + expartner assaults me + expartner assaulted me + expartner assaulting me + ex partner assault me + ex partner assaults me + ex partner assaulted me + ex partner assaulting me + ex assault me + ex assaults me + ex assaulted me + ex assaulting me + spouse assault me + spouse assaults me + spouse assaulted me + spouse assaulting me + ex-spouse assault me + ex-spouse assaults me + ex-spouse assaulted me + ex-spouse assaulting me + exspouse assault me + exspouse assaults me + exspouse assaulted me + exspouse assaulting me + ex spouse assault me + ex spouse assaults me + ex spouse assaulted me + ex spouse assaulting me + significant other assault me + significant other assaults me + significant other assaulted me + significant other assaulting me + partner harass me + partner harasses me + partner harassed me + partner harassing me + ex-partner harass me + ex-partner harasses me + ex-partner harassed me + ex-partner harassing me + expartner harass me + expartner harasses me + expartner harassed me + expartner harassing me + ex partner harass me + ex partner harasses me + ex partner harassed me + ex partner harassing me + ex harass me + ex harasses me + ex harassed me + ex harassing me + spouse harass me + spouse harasses me + spouse harassed me + spouse harassing me + ex-spouse harass me + ex-spouse harasses me + ex-spouse harassed me + ex-spouse harassing me + exspouse harass me + exspouse harasses me + exspouse harassed me + exspouse harassing me + ex spouse harass me + ex spouse harasses me + ex spouse harassed me + ex spouse harassing me + significant other harass me + significant other harasses me + significant other harassed me + significant other harassing me + domestic violence hotline + domestic violence help + domestic violence phone number + domestic abuse phone number + domestic abuse help + domestic abuse hotline + how to leave abuse relationship + signs your relationship is abusive

**Child-witnessed intimate partner violence search terms**

**Physical Abuse**

my mom hit my dad + my mom hits my dad + my mom hitting my dad + my mom hurt my dad + my mom hurts my dad + my mom hurting my dad + my mom kick my dad + my mom kicked my dad + my mom kicks my dad + my mom kicking my dad + my mom beat my dad + my mom beats my dad + my mom beating my dad + my mom slap my dad + my mom slaps my dad + my mom slapping my dad + my mom slapped my dad + my mom grab my dad + my mom grabs my dad + my mom grabbing my dad + my mom grabbed my dad + my mom push my dad + my mom pushes my dad + my mom pushing my dad + my mom pushed my dad + my mom harm my dad + my mom harms my dad + my mom harming my dad + my mom harmed my dad + my mom punch my dad + my mom punches my dad + my mom punching my dad + my mom punched my dad + my mom choke my dad + my mom chokes my dad + my mom choking my dad + my mom choked my dad + my mom strangle my dad + my mom strangles my dad + my mom strangling my dad + my mom strangled my dad + my mom slam my dad + my mom slams my dad + my mom slamming my dad + my mom slammed my dad + my mom point gun at my dad + my mom points gun at my dad + my mom pointing gun at my dad + my mom pointed gun at my dad + my mother hit my father + my mother hits my father + my mother hitting my father + my mother hurt my father + my mother hurts my father + my mother hurting my father + my mother kick my father + my mother kicked my father + my mother kicks my father + my mother kicking my father + my mother beat my father + my mother beats my father + my mother beating my father + my mother slap my father + my mother slaps my father + my mother slapping my father + my mother slapped my father + my mother grab my father + my mother grabs my father + my mother grabbing my father + my mother grabbed my father + my mother push my father + my mother pushes my father + my mother pushing my father + my mother pushed my father + my mother harm my father + my mother harms my father + my mother harming my father + my mother harmed my father + my mother punch my father + my mother punches my father + my mother punching my father + my mother punched my father + my mother choke my father + my mother chokes my father + my mother choking my father + my mother choked my father + my mother strangle my father + my mother strangles my father + my mother strangling my father + my mother strangled my father + my mother slam my father + my mother slams my father + my mother slamming my father + my mother slammed my father + my mother point gun at my father + my mother points gun at my father + my mother pointing gun at my father + my mother pointed gun at my father + my mom hit my step dad + my mom hits my step dad + my mom hitting my step dad + my mom hurt my step dad + my mom hurts my step dad + my mom hurting my step dad + my mom kick my step dad + my mom kicked my step dad + my mom kicks my step dad + my mom kicking my step dad + my mom beat my step dad + my mom beats my step dad + my mom beating my step dad + my mom slap my step dad + my mom slaps my step dad + my mom slapping my step dad + my mom slapped my step dad + my mom grab my step dad + my mom grabs my step dad + my mom grabbing my step dad + my mom grabbed my step dad + my mom push my step dad + my mom pushes my step dad + my mom pushing my step dad + my mom pushed my step dad + my mom harm my step dad + my mom harms my step dad + my mom harming my step dad + my mom harmed my step dad + my mom punch my step dad + my mom punches my step dad + my mom punching my step dad + my mom punched my step dad + my mom choke my step dad + my mom chokes my step dad + my mom choking my step dad + my mom choked my step dad + my mom strangle my step dad + my mom strangles my step dad + my mom strangling my step dad + my mom strangled my step dad + my mom slam my step dad + my mom slams my step dad + my mom slamming my step dad + my mom slammed my step dad + my mom point gun at my step dad + my mom points gun at my step dad + my mom pointing gun at my step dad + my mom pointed gun at my step dad + my mother hit my step father + my mother hits my step father + my mother hitting my step father + my mother hurt my step father + my mother hurts my step father + my mother hurting my step father + my mother kick my step father + my mother kicked my step father + my mother kicks my step father + my mother kicking my step father + my mother beat my step father + my mother beats my step father + my mother beating my step father + my mother slap my step father + my mother slaps my step father + my mother slapping my step father + my mother slapped my step father + my mother grab my step father + my mother grabs my step father + my mother grabbing my step father + my mother grabbed my step father + my mother push my step father + my mother pushes my step father + my mother pushing my step father + my mother pushed my step father + my mother harm my step father + my mother harms my step father + my mother harming my step father + my mother harmed my step father + my mother punch my step father + my mother punches my step father + my mother punching my step father + my mother punched my step father + my mother choke my step father + my mother chokes my step father + my mother choking my step father + my mother choked my step father + my mother strangle my step father + my mother strangles my step father + my mother strangling my step father + my mother strangled my step father + my mother slam my step father + my mother slams my step father + my mother slamming my step father + my mother slammed my step father + my mother point gun at my step father + my mother points gun at my step father + my mother pointing gun at my step father + my mother pointed gun at my step father + my mom hit her boyfriend + my mom hits her boyfriend + my mom hitting her boyfriend + my mom hurt her boyfriend + my mom hurts her boyfriend + my mom hurting her boyfriend + my mom kick her boyfriend + my mom kicked her boyfriend + my mom kicks her boyfriend + my mom kicking her boyfriend + my mom beat her boyfriend + my mom beats her boyfriend + my mom beating her boyfriend + my mom slap her boyfriend + my mom slaps her boyfriend + my mom slapping her boyfriend + my mom slapped her boyfriend + my mom grab her boyfriend + my mom grabs her boyfriend + my mom grabbing her boyfriend + my mom grabbed her boyfriend + my mom push her boyfriend + my mom pushes her boyfriend + my mom pushing her boyfriend + my mom pushed her boyfriend + my mom harm her boyfriend + my mom harms her boyfriend + my mom harming her boyfriend + my mom harmed her boyfriend + my mom punch her boyfriend + my mom punches her boyfriend + my mom punching her boyfriend + my mom punched her boyfriend + my mom choke her boyfriend + my mom chokes her boyfriend + my mom choking her boyfriend + my mom choked her boyfriend + my mom strangle her boyfriend + my mom strangles her boyfriend + my mom strangling her boyfriend + my mom strangled her boyfriend + my mom slam her boyfriend + my mom slams her boyfriend + my mom slamming her boyfriend + my mom slammed her boyfriend + my mom point gun at her boyfriend + my mom points gun at her boyfriend + my mom pointing gun at her boyfriend + my mom pointed gun at her boyfriend + my mother hit her boyfriend + my mother hits her boyfriend + my mother hitting her boyfriend + my mother hurt her boyfriend + my mother hurts her boyfriend + my mother hurting her boyfriend + my mother kick her boyfriend + my mother kicked her boyfriend + my mother kicks her boyfriend + my mother kicking her boyfriend + my mother beat her boyfriend + my mother beats her boyfriend + my mother beating her boyfriend + my mother slap her boyfriend + my mother slaps her boyfriend + my mother slapping her boyfriend + my mother slapped her boyfriend + my mother grab her boyfriend + my mother grabs her boyfriend + my mother grabbing her boyfriend + my mother grabbed her boyfriend + my mother push her boyfriend + my mother pushes her boyfriend + my mother pushing her boyfriend + my mother pushed her boyfriend + my mother harm her boyfriend + my mother harms her boyfriend + my mother harming her boyfriend + my mother harmed her boyfriend + my mother punch her boyfriend + my mother punches her boyfriend + my mother punching her boyfriend + my mother punched her boyfriend + my mother choke her boyfriend + my mother chokes her boyfriend + my mother choking her boyfriend + my mother choked her boyfriend + my mother strangle her boyfriend + my mother strangles her boyfriend + my mother strangling her boyfriend + my mother strangled her boyfriend + my mother slam her boyfriend + my mother slams her boyfriend + my mother slamming her boyfriend + my mother slammed her boyfriend + my mother point gun at her boyfriend + my mother points gun at her boyfriend + my mother pointing gun at her boyfriend + my mother pointed gun at her boyfriend + my mom\'s boyfriend hit her + my mom\'s boyfriend hits her + my mom\'s boyfriend hitting her + my mom\'s boyfriend hurt her + my mom\'s boyfriend hurts her + my mom\'s boyfriend hurting her + my mom\'s boyfriend kick her + my mom\'s boyfriend kicked her + my mom\'s boyfriend kicks her + my mom\'s boyfriend kicking her + my mom\'s boyfriend beat her + my mom\'s boyfriend beats her + my mom\'s boyfriend beating her + my mom\'s boyfriend slap her + my mom\'s boyfriend slaps her + my mom\'s boyfriend slapping her + my mom\'s boyfriend slapped her + my mom\'s boyfriend grab her + my mom\'s boyfriend grabs her + my mom\'s boyfriend grabbing her + my mom\'s boyfriend grabbed her + my mom\'s boyfriend push her + my mom\'s boyfriend pushes her + my mom\'s boyfriend pushing her + my mom\'s boyfriend pushed her + my mom\'s boyfriend harm her + my mom\'s boyfriend harms her + my mom\'s boyfriend harming her + my mom\'s boyfriend harmed her + my mom\'s boyfriend punch her + my mom\'s boyfriend punches her + my mom\'s boyfriend punching her + my mom\'s boyfriend punched her + my mom\'s boyfriend choke her + my mom\'s boyfriend chokes her + my mom\'s boyfriend choking her + my mom\'s boyfriend choked her + my mom\'s boyfriend strangle her + my mom\'s boyfriend strangles her + my mom\'s boyfriend strangling her + my mom\'s boyfriend strangled her + my mom\'s boyfriend slam her + my mom\'s boyfriend slams her + my mom\'s boyfriend slamming her + my mom\'s boyfriend slammed her + my mom\'s boyfriend point gun at her + my mom\'s boyfriend points gun at her + my mom\'s boyfriend pointing gun at her + my mom\'s boyfriend pointed gun at her + my mother\'s boyfriend hit her + my mother\'s boyfriend hits her + my mother\'s boyfriend hitting her + my mother\'s boyfriend hurt her + my mother\'s boyfriend hurts her + my mother\'s boyfriend hurting her + my mother\'s boyfriend kick her + my mother\'s boyfriend kicked her + my mother\'s boyfriend kicks her + my mother\'s boyfriend kicking her + my mother\'s boyfriend beat her + my mother\'s boyfriend beats her + my mother\'s boyfriend beating her + my mother\'s boyfriend slap her + my mother\'s boyfriend slaps her + my mother\'s boyfriend slapping her + my mother\'s boyfriend slapped her + my mother\'s boyfriend grab her + my mother\'s boyfriend grabs her + my mother\'s boyfriend grabbing her + my mother\'s boyfriend grabbed her + my mother\'s boyfriend push her + my mother\'s boyfriend pushes her + my mother\'s boyfriend pushing her + my mother\'s boyfriend pushed her + my mother\'s boyfriend harm her + my mother\'s boyfriend harms her + my mother\'s boyfriend harming her + my mother\'s boyfriend harmed her + my mother\'s boyfriend punch her + my mother\'s boyfriend punches her + my mother\'s boyfriend punching her + my mother\'s boyfriend punched her + my mother\'s boyfriend choke her + my mother\'s boyfriend chokes her + my mother\'s boyfriend choking her + my mother\'s boyfriend choked her + my mother\'s boyfriend strangle her + my mother\'s boyfriend strangles her + my mother\'s boyfriend strangling her + my mother\'s boyfriend strangled her + my mother\'s boyfriend slam her + my mother\'s boyfriend slams her + my mother\'s boyfriend slamming her + my mother\'s boyfriend slammed her + my mother\'s boyfriend point gun at her + my mother\'s boyfriend points gun at her + my mother\'s boyfriend pointing gun at her + my mother\'s boyfriend pointed gun at her + my mom hit her girlfriend + my mom hits her girlfriend + my mom hitting her girlfriend + my mom hurt her girlfriend + my mom hurts her girlfriend + my mom hurting her girlfriend + my mom kick her girlfriend + my mom kicked her girlfriend + my mom kicks her girlfriend + my mom kicking her girlfriend + my mom beat her girlfriend + my mom beats her girlfriend + my mom beating her girlfriend + my mom slap her girlfriend + my mom slaps her girlfriend + my mom slapping her girlfriend + my mom slapped her girlfriend + my mom grab her girlfriend + my mom grabs her girlfriend + my mom grabbing her girlfriend + my mom grabbed her girlfriend + my mom push her girlfriend + my mom pushes her girlfriend + my mom pushing her girlfriend + my mom pushed her girlfriend + my mom harm her girlfriend + my mom harms her girlfriend + my mom harming her girlfriend + my mom harmed her girlfriend + my mom punch her girlfriend + my mom punches her girlfriend + my mom punching her girlfriend + my mom punched her girlfriend + my mom choke her girlfriend + my mom chokes her girlfriend + my mom choking her girlfriend + my mom choked her girlfriend + my mom strangle her girlfriend + my mom strangles her girlfriend + my mom strangling her girlfriend + my mom strangled her girlfriend + my mom slam her girlfriend + my mom slams her girlfriend + my mom slamming her girlfriend + my mom slammed her girlfriend + my mom point gun at her girlfriend + my mom points gun at her girlfriend + my mom pointing gun at her girlfriend + my mom pointed gun at her girlfriend + my mother hit her girlfriend + my mother hits her girlfriend + my mother hitting her girlfriend + my mother hurt her girlfriend + my mother hurts her girlfriend + my mother hurting her girlfriend + my mother kick her girlfriend + my mother kicked her girlfriend + my mother kicks her girlfriend + my mother kicking her girlfriend + my mother beat her girlfriend + my mother beats her girlfriend + my mother beating her girlfriend + my mother slap her girlfriend + my mother slaps her girlfriend + my mother slapping her girlfriend + my mother slapped her girlfriend + my mother grab her girlfriend + my mother grabs her girlfriend + my mother grabbing her girlfriend + my mother grabbed her girlfriend + my mother push her girlfriend + my mother pushes her girlfriend + my mother pushing her girlfriend + my mother pushed her girlfriend + my mother harm her girlfriend + my mother harms her girlfriend + my mother harming her girlfriend + my mother harmed her girlfriend + my mother punch her girlfriend + my mother punches her girlfriend + my mother punching her girlfriend + my mother punched her girlfriend + my mother choke her girlfriend + my mother chokes her girlfriend + my mother choking her girlfriend + my mother choked her girlfriend + my mother strangle her girlfriend + my mother strangles her girlfriend + my mother strangling her girlfriend + my mother strangled her girlfriend + my mother slam her girlfriend + my mother slams her girlfriend + my mother slamming her girlfriend + my mother slammed her girlfriend + my mother point gun at her girlfriend + my mother points gun at her girlfriend + my mother pointing gun at her girlfriend + my mother pointed gun at her girlfriend + my mom\'s girlfriend hit her + my mom\'s girlfriend hits her + my mom\'s girlfriend hitting her + my mom\'s girlfriend hurt her + my mom\'s girlfriend hurts her + my mom\'s girlfriend hurting her + my mom\'s girlfriend kick her + my mom\'s girlfriend kicked her + my mom\'s girlfriend kicks her + my mom\'s girlfriend kicking her + my mom\'s girlfriend beat her + my mom\'s girlfriend beats her + my mom\'s girlfriend beating her + my mom\'s girlfriend slap her + my mom\'s girlfriend slaps her + my mom\'s girlfriend slapping her + my mom\'s girlfriend slapped her + my mom\'s girlfriend grab her + my mom\'s girlfriend grabs her + my mom\'s girlfriend grabbing her + my mom\'s girlfriend grabbed her + my mom\'s girlfriend push her + my mom\'s girlfriend pushes her + my mom\'s girlfriend pushing her + my mom\'s girlfriend pushed her + my mom\'s girlfriend harm her + my mom\'s girlfriend harms her + my mom\'s girlfriend harming her + my mom\'s girlfriend harmed her + my mom\'s girlfriend punch her + my mom\'s girlfriend punches her + my mom\'s girlfriend punching her + my mom\'s girlfriend punched her + my mom\'s girlfriend choke her + my mom\'s girlfriend chokes her + my mom\'s girlfriend choking her + my mom\'s girlfriend choked her + my mom\'s girlfriend strangle her + my mom\'s girlfriend strangles her + my mom\'s girlfriend strangling her + my mom\'s girlfriend strangled her + my mom\'s girlfriend slam her + my mom\'s girlfriend slams her + my mom\'s girlfriend slamming her + my mom\'s girlfriend slammed her + my mom\'s girlfriend point gun at her + my mom\'s girlfriend points gun at her + my mom\'s girlfriend pointing gun at her + my mom\'s girlfriend pointed gun at her + my mother\'s girlfriend hit her + my mother\'s girlfriend hits her + my mother\'s girlfriend hitting her + my mother\'s girlfriend hurt her + my mother\'s girlfriend hurts her + my mother\'s girlfriend hurting her + my mother\'s girlfriend kick her + my mother\'s girlfriend kicked her + my mother\'s girlfriend kicks her + my mother\'s girlfriend kicking her + my mother\'s girlfriend beat her + my mother\'s girlfriend beats her + my mother\'s girlfriend beating her + my mother\'s girlfriend slap her + my mother\'s girlfriend slaps her + my mother\'s girlfriend slapping her + my mother\'s girlfriend slapped her + my mother\'s girlfriend grab her + my mother\'s girlfriend grabs her + my mother\'s girlfriend grabbing her + my mother\'s girlfriend grabbed her + my mother\'s girlfriend push her + my mother\'s girlfriend pushes her + my mother\'s girlfriend pushing her + my mother\'s girlfriend pushed her + my mother\'s girlfriend harm her + my mother\'s girlfriend harms her + my mother\'s girlfriend harming her + my mother\'s girlfriend harmed her + my mother\'s girlfriend punch her + my mother\'s girlfriend punches her + my mother\'s girlfriend punching her + my mother\'s girlfriend punched her + my mother\'s girlfriend choke her + my mother\'s girlfriend chokes her + my mother\'s girlfriend choking her + my mother\'s girlfriend choked her + my mother\'s girlfriend strangle her + my mother\'s girlfriend strangles her + my mother\'s girlfriend strangling her + my mother\'s girlfriend strangled her + my mother\'s girlfriend slam her + my mother\'s girlfriend slams her + my mother\'s girlfriend slamming her + my mother\'s girlfriend slammed her + my mother\'s girlfriend point gun at her + my mother\'s girlfriend points gun at her + my mother\'s girlfriend pointing gun at her + my mother\'s girlfriend pointed gun at her + my mom hit my mom + my mom hits my mom + my mom hitting my mom + my mom hurt my mom + my mom hurts my mom + my mom hurting my mom + my mom kick my mom + my mom kicked my mom + my mom kicks my mom + my mom kicking my mom + my mom beat my mom + my mom beats my mom + my mom beating my mom + my mom slap my mom + my mom slaps my mom + my mom slapping my mom + my mom slapped my mom + my mom grab my mom + my mom grabs my mom + my mom grabbing my mom + my mom grabbed my mom + my mom push my mom + my mom pushes my mom + my mom pushing my mom + my mom pushed my mom + my mom harm my mom + my mom harms my mom + my mom harming my mom + my mom harmed my mom + my mom punch my mom + my mom punches my mom + my mom punching my mom + my mom punched my mom + my mom choke my mom + my mom chokes my mom + my mom choking my mom + my mom choked my mom + my mom strangle my mom + my mom strangles my mom + my mom strangling my mom + my mom strangled my mom + my mom slam my mom + my mom slams my mom + my mom slamming my mom + my mom slammed my mom + my mom point gun at my mom + my mom points gun at my mom + my mom pointing gun at my mom + my mom pointed gun at my mom + my mom hit my mother + my mom hits my mother + my mom hitting my mother + my mom hurt my mother + my mom hurts my mother + my mom hurting my mother + my mom kick my mother + my mom kicked my mother + my mom kicks my mother + my mom kicking my mother + my mom beat my mother + my mom beats my mother + my mom beating my mother + my mom slap my mother + my mom slaps my mother + my mom slapping my mother + my mom slapped my mother + my mom grab my mother + my mom grabs my mother + my mom grabbing my mother + my mom grabbed my mother + my mom push my mother + my mom pushes my mother + my mom pushing my mother + my mom pushed my mother + my mom harm my mother + my mom harms my mother + my mom harming my mother + my mom harmed my mother + my mom punch my mother + my mom punches my mother + my mom punching my mother + my mom punched my mother + my mom choke my mother + my mom chokes my mother + my mom choking my mother + my mom choked my mother + my mom strangle my mother + my mom strangles my mother + my mom strangling my mother + my mom strangled my mother + my mom slam my mother + my mom slams my mother + my mom slamming my mother + my mom slammed my mother + my mom point gun at my mother + my mom points gun at my mother + my mom pointing gun at my mother + my mom pointed gun at my mother + my mother hit my mother + my mother hits my mother + my mother hitting my mother + my mother hurt my mother + my mother hurts my mother + my mother hurting my mother + my mother kick my mother + my mother kicked my mother + my mother kicks my mother + my mother kicking my mother + my mother beat my mother + my mother beats my mother + my mother beating my mother + my mother slap my mother + my mother slaps my mother + my mother slapping my mother + my mother slapped my mother + my mother grab my mother + my mother grabs my mother + my mother grabbing my mother + my mother grabbed my mother + my mother push my mother + my mother pushes my mother + my mother pushing my mother + my mother pushed my mother + my mother harm my mother + my mother harms my mother + my mother harming my mother + my mother harmed my mother + my mother punch my mother + my mother punches my mother + my mother punching my mother + my mother punched my mother + my mother choke my mother + my mother chokes my mother + my mother choking my mother + my mother choked my mother + my mother strangle my mother + my mother strangles my mother + my mother strangling my mother + my mother strangled my mother + my mother slam my mother + my mother slams my mother + my mother slamming my mother + my mother slammed my mother + my mother point gun at my mother + my mother points gun at my mother + my mother pointing gun at my mother + my mother pointed gun at my mother + my dad hit my step mom + my dad hits my step mom + my dad hitting my step mom + my dad hurt my step mom + my dad hurts my step mom + my dad hurting my step mom + my dad kick my step mom + my dad kicked my step mom + my dad kicks my step mom + my dad kicking my step mom + my dad beat my step mom + my dad beats my step mom + my dad beating my step mom + my dad slap my step mom + my dad slaps my step mom + my dad slapping my step mom + my dad slapped my step mom + my dad grab my step mom + my dad grabs my step mom + my dad grabbing my step mom + my dad grabbed my step mom + my dad push my step mom + my dad pushes my step mom + my dad pushing my step mom + my dad pushed my step mom + my dad harm my step mom + my dad harms my step mom + my dad harming my step mom + my dad harmed my step mom + my dad punch my step mom + my dad punches my step mom + my dad punching my step mom + my dad punched my step mom + my dad choke my step mom + my dad chokes my step mom + my dad choking my step mom + my dad choked my step mom + my dad strangle my step mom + my dad strangles my step mom + my dad strangling my step mom + my dad strangled my step mom + my dad slam my step mom + my dad slams my step mom + my dad slamming my step mom + my dad slammed my step mom + my dad point gun at my step mom + my dad points gun at my step mom + my dad pointing gun at my step mom + my dad pointed gun at my step mom + my father hit my step mother + my father hits my step mother + my father hitting my step mother + my father hurt my step mother + my father hurts my step mother + my father hurting my step mother + my father kick my step mother + my father kicked my step mother + my father kicks my step mother + my father kicking my step mother + my father beat my step mother + my father beats my step mother + my father beating my step mother + my father slap my step mother + my father slaps my step mother + my father slapping my step mother + my father slapped my step mother + my father grab my step mother + my father grabs my step mother + my father grabbing my step mother + my father grabbed my step mother + my father push my step mother + my father pushes my step mother + my father pushing my step mother + my father pushed my step mother + my father harm my step mother + my father harms my step mother + my father harming my step mother + my father harmed my step mother + my father punch my step mother + my father punches my step mother + my father punching my step mother + my father punched my step mother + my father choke my step mother + my father chokes my step mother + my father choking my step mother + my father choked my step mother + my father strangle my step mother + my father strangles my step mother + my father strangling my step mother + my father strangled my step mother + my father slam my step mother + my father slams my step mother + my father slamming my step mother + my father slammed my step mother + my father point gun at my step mother + my father points gun at my step mother + my father pointing gun at my step mother + my father pointed gun at my step mother + my dad hit his girlfriend + my dad hits his girlfriend + my dad hitting his girlfriend + my dad hurt his girlfriend + my dad hurts his girlfriend + my dad hurting his girlfriend + my dad kick his girlfriend + my dad kicked his girlfriend + my dad kicks his girlfriend + my dad kicking his girlfriend + my dad beat his girlfriend + my dad beats his girlfriend + my dad beating his girlfriend + my dad slap his girlfriend + my dad slaps his girlfriend + my dad slapping his girlfriend + my dad slapped his girlfriend + my dad grab his girlfriend + my dad grabs his girlfriend + my dad grabbing his girlfriend + my dad grabbed his girlfriend + my dad push his girlfriend + my dad pushes his girlfriend + my dad pushing his girlfriend + my dad pushed his girlfriend + my dad harm his girlfriend + my dad harms his girlfriend + my dad harming his girlfriend + my dad harmed his girlfriend + my dad punch his girlfriend + my dad punches his girlfriend + my dad punching his girlfriend + my dad punched his girlfriend + my dad choke his girlfriend + my dad chokes his girlfriend + my dad choking his girlfriend + my dad choked his girlfriend + my dad strangle his girlfriend + my dad strangles his girlfriend + my dad strangling his girlfriend + my dad strangled his girlfriend + my dad slam his girlfriend + my dad slams his girlfriend + my dad slamming his girlfriend + my dad slammed his girlfriend + my dad point gun at his girlfriend + my dad points gun at his girlfriend + my dad pointing gun at his girlfriend + my dad pointed gun at his girlfriend + my father hit his girlfriend + my father hits his girlfriend + my father hitting his girlfriend + my father hurt his girlfriend + my father hurts his girlfriend + my father hurting his girlfriend + my father kick his girlfriend + my father kicked his girlfriend + my father kicks his girlfriend + my father kicking his girlfriend + my father beat his girlfriend + my father beats his girlfriend + my father beating his girlfriend + my father slap his girlfriend + my father slaps his girlfriend + my father slapping his girlfriend + my father slapped his girlfriend + my father grab his girlfriend + my father grabs his girlfriend + my father grabbing his girlfriend + my father grabbed his girlfriend + my father push his girlfriend + my father pushes his girlfriend + my father pushing his girlfriend + my father pushed his girlfriend + my father harm his girlfriend + my father harms his girlfriend + my father harming his girlfriend + my father harmed his girlfriend + my father punch his girlfriend + my father punches his girlfriend + my father punching his girlfriend + my father punched his girlfriend + my father choke his girlfriend + my father chokes his girlfriend + my father choking his girlfriend + my father choked his girlfriend + my father strangle his girlfriend + my father strangles his girlfriend + my father strangling his girlfriend + my father strangled his girlfriend + my father slam his girlfriend + my father slams his girlfriend + my father slamming his girlfriend + my father slammed his girlfriend + my father point gun at his girlfriend + my father points gun at his girlfriend + my father pointing gun at his girlfriend + my father pointed gun at his girlfriend + my dad\'s girlfriend hit him + my dad\'s girlfriend hits him + my dad\'s girlfriend hitting him + my dad\'s girlfriend hurt him + my dad\'s girlfriend hurts him + my dad\'s girlfriend hurting him + my dad\'s girlfriend kick him + my dad\'s girlfriend kicked him + my dad\'s girlfriend kicks him + my dad\'s girlfriend kicking him + my dad\'s girlfriend beat him + my dad\'s girlfriend beats him + my dad\'s girlfriend beating him + my dad\'s girlfriend slap him + my dad\'s girlfriend slaps him + my dad\'s girlfriend slapping him + my dad\'s girlfriend slapped him + my dad\'s girlfriend grab him + my dad\'s girlfriend grabs him + my dad\'s girlfriend grabbing him + my dad\'s girlfriend grabbed him + my dad\'s girlfriend push him + my dad\'s girlfriend pushes him + my dad\'s girlfriend pushing him + my dad\'s girlfriend pushed him + my dad\'s girlfriend harm him + my dad\'s girlfriend harms him + my dad\'s girlfriend harming him + my dad\'s girlfriend harmed him + my dad\'s girlfriend punch him + my dad\'s girlfriend punches him + my dad\'s girlfriend punching him + my dad\'s girlfriend punched him + my dad\'s girlfriend choke him + my dad\'s girlfriend chokes him + my dad\'s girlfriend choking him + my dad\'s girlfriend choked him + my dad\'s girlfriend strangle him + my dad\'s girlfriend strangles him + my dad\'s girlfriend strangling him + my dad\'s girlfriend strangled him + my dad\'s girlfriend slam him + my dad\'s girlfriend slams him + my dad\'s girlfriend slamming him + my dad\'s girlfriend slammed him + my dad\'s girlfriend point gun at him + my dad\'s girlfriend points gun at him + my dad\'s girlfriend pointing gun at him + my dad\'s girlfriend pointed gun at him + my father\'s girlfriend hit him + my father\'s girlfriend hits him + my father\'s girlfriend hitting him + my father\'s girlfriend hurt him + my father\'s girlfriend hurts him + my father\'s girlfriend hurting him + my father\'s girlfriend kick him + my father\'s girlfriend kicked him + my father\'s girlfriend kicks him + my father\'s girlfriend kicking him + my father\'s girlfriend beat him + my father\'s girlfriend beats him + my father\'s girlfriend beating him + my father\'s girlfriend slap him + my father\'s girlfriend slaps him + my father\'s girlfriend slapping him + my father\'s girlfriend slapped him + my father\'s girlfriend grab him + my father\'s girlfriend grabs him + my father\'s girlfriend grabbing him + my father\'s girlfriend grabbed him + my father\'s girlfriend push him + my father\'s girlfriend pushes him + my father\'s girlfriend pushing him + my father\'s girlfriend pushed him + my father\'s girlfriend harm him + my father\'s girlfriend harms him + my father\'s girlfriend harming him + my father\'s girlfriend harmed him + my father\'s girlfriend punch him + my father\'s girlfriend punches him + my father\'s girlfriend punching him + my father\'s girlfriend punched him + my father\'s girlfriend choke him + my father\'s girlfriend chokes him + my father\'s girlfriend choking him + my father\'s girlfriend choked him + my father\'s girlfriend strangle him + my father\'s girlfriend strangles him + my father\'s girlfriend strangling him + my father\'s girlfriend strangled him + my father\'s girlfriend slam him + my father\'s girlfriend slams him + my father\'s girlfriend slamming him + my father\'s girlfriend slammed him + my father\'s girlfriend point gun at him + my father\'s girlfriend points gun at him + my father\'s girlfriend pointing gun at him + my father\'s girlfriend pointed gun at him + my dad hit his boyfriend + my dad hits his boyfriend + my dad hitting his boyfriend + my dad hurt his boyfriend + my dad hurts his boyfriend + my dad hurting his boyfriend + my dad kick his boyfriend + my dad kicked his boyfriend + my dad kicks his boyfriend + my dad kicking his boyfriend + my dad beat his boyfriend + my dad beats his boyfriend + my dad beating his boyfriend + my dad slap his boyfriend + my dad slaps his boyfriend + my dad slapping his boyfriend + my dad slapped his boyfriend + my dad grab his boyfriend + my dad grabs his boyfriend + my dad grabbing his boyfriend + my dad grabbed his boyfriend + my dad push his boyfriend + my dad pushes his boyfriend + my dad pushing his boyfriend + my dad pushed his boyfriend + my dad harm his boyfriend + my dad harms his boyfriend + my dad harming his boyfriend + my dad harmed his boyfriend + my dad punch his boyfriend + my dad punches his boyfriend + my dad punching his boyfriend + my dad punched his boyfriend + my dad choke his boyfriend + my dad chokes his boyfriend + my dad choking his boyfriend + my dad choked his boyfriend + my dad strangle his boyfriend + my dad strangles his boyfriend + my dad strangling his boyfriend + my dad strangled his boyfriend + my dad slam his boyfriend + my dad slams his boyfriend + my dad slamming his boyfriend + my dad slammed his boyfriend + my dad point gun at his boyfriend + my dad points gun at his boyfriend + my dad pointing gun at his boyfriend + my dad pointed gun at his boyfriend + my father hit his boyfriend + my father hits his boyfriend + my father hitting his boyfriend + my father hurt his boyfriend + my father hurts his boyfriend + my father hurting his boyfriend + my father kick his boyfriend + my father kicked his boyfriend + my father kicks his boyfriend + my father kicking his boyfriend + my father beat his boyfriend + my father beats his boyfriend + my father beating his boyfriend + my father slap his boyfriend + my father slaps his boyfriend + my father slapping his boyfriend + my father slapped his boyfriend + my father grab his boyfriend + my father grabs his boyfriend + my father grabbing his boyfriend + my father grabbed his boyfriend + my father push his boyfriend + my father pushes his boyfriend + my father pushing his boyfriend + my father pushed his boyfriend + my father harm his boyfriend + my father harms his boyfriend + my father harming his boyfriend + my father harmed his boyfriend + my father punch his boyfriend + my father punches his boyfriend + my father punching his boyfriend + my father punched his boyfriend + my father choke his boyfriend + my father chokes his boyfriend + my father choking his boyfriend + my father choked his boyfriend + my father strangle his boyfriend + my father strangles his boyfriend + my father strangling his boyfriend + my father strangled his boyfriend + my father slam his boyfriend + my father slams his boyfriend + my father slamming his boyfriend + my father slammed his boyfriend + my father point gun at his boyfriend + my father points gun at his boyfriend + my father pointing gun at his boyfriend + my father pointed gun at his boyfriend + my dad\'s boyfriend hit him + my dad\'s boyfriend hits him + my dad\'s boyfriend hitting him + my dad\'s boyfriend hurt him + my dad\'s boyfriend hurts him + my dad\'s boyfriend hurting him + my dad\'s boyfriend kick him + my dad\'s boyfriend kicked him + my dad\'s boyfriend kicks him + my dad\'s boyfriend kicking him + my dad\'s boyfriend beat him + my dad\'s boyfriend beats him + my dad\'s boyfriend beating him + my dad\'s boyfriend slap him + my dad\'s boyfriend slaps him + my dad\'s boyfriend slapping him + my dad\'s boyfriend slapped him + my dad\'s boyfriend grab him + my dad\'s boyfriend grabs him + my dad\'s boyfriend grabbing him + my dad\'s boyfriend grabbed him + my dad\'s boyfriend push him + my dad\'s boyfriend pushes him + my dad\'s boyfriend pushing him + my dad\'s boyfriend pushed him + my dad\'s boyfriend harm him + my dad\'s boyfriend harms him + my dad\'s boyfriend harming him + my dad\'s boyfriend harmed him + my dad\'s boyfriend punch him + my dad\'s boyfriend punches him + my dad\'s boyfriend punching him + my dad\'s boyfriend punched him + my dad\'s boyfriend choke him + my dad\'s boyfriend chokes him + my dad\'s boyfriend choking him + my dad\'s boyfriend choked him + my dad\'s boyfriend strangle him + my dad\'s boyfriend strangles him + my dad\'s boyfriend strangling him + my dad\'s boyfriend strangled him + my dad\'s boyfriend slam him + my dad\'s boyfriend slams him + my dad\'s boyfriend slamming him + my dad\'s boyfriend slammed him + my dad\'s boyfriend point gun at him + my dad\'s boyfriend points gun at him + my dad\'s boyfriend pointing gun at him + my dad\'s boyfriend pointed gun at him + my father\'s boyfriend hit him + my father\'s boyfriend hits him + my father\'s boyfriend hitting him + my father\'s boyfriend hurt him + my father\'s boyfriend hurts him + my father\'s boyfriend hurting him + my father\'s boyfriend kick him + my father\'s boyfriend kicked him + my father\'s boyfriend kicks him + my father\'s boyfriend kicking him + my father\'s boyfriend beat him + my father\'s boyfriend beats him + my father\'s boyfriend beating him + my father\'s boyfriend slap him + my father\'s boyfriend slaps him + my father\'s boyfriend slapping him + my father\'s boyfriend slapped him + my father\'s boyfriend grab him + my father\'s boyfriend grabs him + my father\'s boyfriend grabbing him + my father\'s boyfriend grabbed him + my father\'s boyfriend push him + my father\'s boyfriend pushes him + my father\'s boyfriend pushing him + my father\'s boyfriend pushed him + my father\'s boyfriend harm him + my father\'s boyfriend harms him + my father\'s boyfriend harming him + my father\'s boyfriend harmed him + my father\'s boyfriend punch him + my father\'s boyfriend punches him + my father\'s boyfriend punching him + my father\'s boyfriend punched him + my father\'s boyfriend choke him + my father\'s boyfriend chokes him + my father\'s boyfriend choking him + my father\'s boyfriend choked him + my father\'s boyfriend strangle him + my father\'s boyfriend strangles him + my father\'s boyfriend strangling him + my father\'s boyfriend strangled him + my father\'s boyfriend slam him + my father\'s boyfriend slams him + my father\'s boyfriend slamming him + my father\'s boyfriend slammed him + my father\'s boyfriend point gun at him + my father\'s boyfriend points gun at him + my father\'s boyfriend pointing gun at him + my father\'s boyfriend pointed gun at him + my dad hit my dad + my dad hits my dad + my dad hitting my dad + my dad hurt my dad + my dad hurts my dad + my dad hurting my dad + my dad kick my dad + my dad kicked my dad + my dad kicks my dad + my dad kicking my dad + my dad beat my dad + my dad beats my dad + my dad beating my dad + my dad slap my dad + my dad slaps my dad + my dad slapping my dad + my dad slapped my dad + my dad grab my dad + my dad grabs my dad + my dad grabbing my dad + my dad grabbed my dad + my dad push my dad + my dad pushes my dad + my dad pushing my dad + my dad pushed my dad + my dad harm my dad + my dad harms my dad + my dad harming my dad + my dad harmed my dad + my dad punch my dad + my dad punches my dad + my dad punching my dad + my dad punched my dad + my dad choke my dad + my dad chokes my dad + my dad choking my dad + my dad choked my dad + my dad strangle my dad + my dad strangles my dad + my dad strangling my dad + my dad strangled my dad + my dad slam my dad + my dad slams my dad + my dad slamming my dad + my dad slammed my dad + my dad point gun at my dad + my dad points gun at my dad + my dad pointing gun at my dad + my dad pointed gun at my dad + my dad hit my father + my dad hits my father + my dad hitting my father + my dad hurt my father + my dad hurts my father + my dad hurting my father + my dad kick my father + my dad kicked my father + my dad kicks my father + my dad kicking my father + my dad beat my father + my dad beats my father + my dad beating my father + my dad slap my father + my dad slaps my father + my dad slapping my father + my dad slapped my father + my dad grab my father + my dad grabs my father + my dad grabbing my father + my dad grabbed my father + my dad push my father + my dad pushes my father + my dad pushing my father + my dad pushed my father + my dad harm my father + my dad harms my father + my dad harming my father + my dad harmed my father + my dad punch my father + my dad punches my father + my dad punching my father + my dad punched my father + my dad choke my father + my dad chokes my father + my dad choking my father + my dad choked my father + my dad strangle my father + my dad strangles my father + my dad strangling my father + my dad strangled my father + my dad slam my father + my dad slams my father + my dad slamming my father + my dad slammed my father + my dad point gun at my father + my dad points gun at my father + my dad pointing gun at my father + my dad pointed gun at my father + my father hit my father + my father hits my father + my father hitting my father + my father hurt my father + my father hurts my father + my father hurting my father + my father kick my father + my father kicked my father + my father kicks my father + my father kicking my father + my father beat my father + my father beats my father + my father beating my father + my father slap my father + my father slaps my father + my father slapping my father + my father slapped my father + my father grab my father + my father grabs my father + my father grabbing my father + my father grabbed my father + my father push my father + my father pushes my father + my father pushing my father + my father pushed my father + my father harm my father + my father harms my father + my father harming my father + my father harmed my father + my father punch my father + my father punches my father + my father punching my father + my father punched my father + my father choke my father + my father chokes my father + my father choking my father + my father choked my father + my father strangle my father + my father strangles my father + my father strangling my father + my father strangled my father + my father slam my father + my father slams my father + my father slamming my father + my father slammed my father + my father point gun at my father + my father points gun at my father + my father pointing gun at my father + my father pointed gun at my father + my mom\'s friend hit her + my mom\'s friend hits her + my mom\'s friend hitting her + my mom\'s friend hurt her + my mom\'s friend hurts her + my mom\'s friend hurting her + my mom\'s friend kick her + my mom\'s friend kicked her + my mom\'s friend kicks her + my mom\'s friend kicking her + my mom\'s friend beat her + my mom\'s friend beats her + my mom\'s friend beating her + my mom\'s friend slap her + my mom\'s friend slaps her + my mom\'s friend slapping her + my mom\'s friend slapped her + my mom\'s friend grab her + my mom\'s friend grabs her + my mom\'s friend grabbing her + my mom\'s friend grabbed her + my mom\'s friend push her + my mom\'s friend pushes her + my mom\'s friend pushing her + my mom\'s friend pushed her + my mom\'s friend harm her + my mom\'s friend harms her + my mom\'s friend harming her + my mom\'s friend harmed her + my mom\'s friend punch her + my mom\'s friend punches her + my mom\'s friend punching her + my mom\'s friend punched her + my mom\'s friend choke her + my mom\'s friend chokes her + my mom\'s friend choking her + my mom\'s friend choked her + my mom\'s friend strangle her + my mom\'s friend strangles her + my mom\'s friend strangling her + my mom\'s friend strangled her + my mom\'s friend slam her + my mom\'s friend slams her + my mom\'s friend slamming her + my mom\'s friend slammed her + my mom\'s friend point gun at her + my mom\'s friend points gun at her + my mom\'s friend pointing gun at her + my mom\'s friend pointed gun at her + my mother\'s friend hit her + my mother\'s friend hits her + my mother\'s friend hitting her + my mother\'s friend hurt her + my mother\'s friend hurts her + my mother\'s friend hurting her + my mother\'s friend kick her + my mother\'s friend kicked her + my mother\'s friend kicks her + my mother\'s friend kicking her + my mother\'s friend beat her + my mother\'s friend beats her + my mother\'s friend beating her + my mother\'s friend slap her + my mother\'s friend slaps her + my mother\'s friend slapping her + my mother\'s friend slapped her + my mother\'s friend grab her + my mother\'s friend grabs her + my mother\'s friend grabbing her + my mother\'s friend grabbed her + my mother\'s friend push her + my mother\'s friend pushes her + my mother\'s friend pushing her + my mother\'s friend pushed her + my mother\'s friend harm her + my mother\'s friend harms her + my mother\'s friend harming her + my mother\'s friend harmed her + my mother\'s friend punch her + my mother\'s friend punches her + my mother\'s friend punching her + my mother\'s friend punched her + my mother\'s friend choke her + my mother\'s friend chokes her + my mother\'s friend choking her + my mother\'s friend choked her + my mother\'s friend strangle her + my mother\'s friend strangles her + my mother\'s friend strangling her + my mother\'s friend strangled her + my mother\'s friend slam her + my mother\'s friend slams her + my mother\'s friend slamming her + my mother\'s friend slammed her + my mother\'s friend point gun at her + my mother\'s friend points gun at her + my mother\'s friend pointing gun at her + my mother\'s friend pointed gun at her + my dad hit his friend + my dad hits his friend + my dad hitting his friend + my dad hurt his friend + my dad hurts his friend + my dad hurting his friend + my dad kick his friend + my dad kicked his friend + my dad kicks his friend + my dad kicking his friend + my dad beat his friend + my dad beats his friend + my dad beating his friend + my dad slap his friend + my dad slaps his friend + my dad slapping his friend + my dad slapped his friend + my dad grab his friend + my dad grabs his friend + my dad grabbing his friend + my dad grabbed his friend + my dad push his friend + my dad pushes his friend + my dad pushing his friend + my dad pushed his friend + my dad harm his friend + my dad harms his friend + my dad harming his friend + my dad harmed his friend + my dad punch his friend + my dad punches his friend + my dad punching his friend + my dad punched his friend + my dad choke his friend + my dad chokes his friend + my dad choking his friend + my dad choked his friend + my dad strangle his friend + my dad strangles his friend + my dad strangling his friend + my dad strangled his friend + my dad slam his friend + my dad slams his friend + my dad slamming his friend + my dad slammed his friend + my dad point gun at his friend + my dad points gun at his friend + my dad pointing gun at his friend + my dad pointed gun at his friend + my father hit his friend + my father hits his friend + my father hitting his friend + my father hurt his friend + my father hurts his friend + my father hurting his friend + my father kick his friend + my father kicked his friend + my father kicks his friend + my father kicking his friend + my father beat his friend + my father beats his friend + my father beating his friend + my father slap his friend + my father slaps his friend + my father slapping his friend + my father slapped his friend + my father grab his friend + my father grabs his friend + my father grabbing his friend + my father grabbed his friend + my father push his friend + my father pushes his friend + my father pushing his friend + my father pushed his friend + my father harm his friend + my father harms his friend + my father harming his friend + my father harmed his friend + my father punch his friend + my father punches his friend + my father punching his friend + my father punched his friend + my father choke his friend + my father chokes his friend + my father choking his friend + my father choked his friend + my father strangle his friend + my father strangles his friend + my father strangling his friend + my father strangled his friend + my father slam his friend + my father slams his friend + my father slamming his friend + my father slammed his friend + my father point gun at his friend + my father points gun at his friend + my father pointing gun at his friend + my father pointed gun at his friend + my dad\'s friend hit him + my dad\'s friend hits him + my dad\'s friend hitting him + my dad\'s friend hurt him + my dad\'s friend hurts him + my dad\'s friend hurting him + my dad\'s friend kick him + my dad\'s friend kicked him + my dad\'s friend kicks him + my dad\'s friend kicking him + my dad\'s friend beat him + my dad\'s friend beats him + my dad\'s friend beating him + my dad\'s friend slap him + my dad\'s friend slaps him + my dad\'s friend slapping him + my dad\'s friend slapped him + my dad\'s friend grab him + my dad\'s friend grabs him + my dad\'s friend grabbing him + my dad\'s friend grabbed him + my dad\'s friend push him + my dad\'s friend pushes him + my dad\'s friend pushing him + my dad\'s friend pushed him + my dad\'s friend harm him + my dad\'s friend harms him + my dad\'s friend harming him + my dad\'s friend harmed him + my dad\'s friend punch him + my dad\'s friend punches him + my dad\'s friend punching him + my dad\'s friend punched him + my dad\'s friend choke him + my dad\'s friend chokes him + my dad\'s friend choking him + my dad\'s friend choked him + my dad\'s friend strangle him + my dad\'s friend strangles him + my dad\'s friend strangling him + my dad\'s friend strangled him + my dad\'s friend slam him + my dad\'s friend slams him + my dad\'s friend slamming him + my dad\'s friend slammed him + my dad\'s friend point gun at him + my dad\'s friend points gun at him + my dad\'s friend pointing gun at him + my dad\'s friend pointed gun at him + my father\'s friend hit him + my father\'s friend hits him + my father\'s friend hitting him + my father\'s friend hurt him + my father\'s friend hurts him + my father\'s friend hurting him + my father\'s friend kick him + my father\'s friend kicked him + my father\'s friend kicks him + my father\'s friend kicking him + my father\'s friend beat him + my father\'s friend beats him + my father\'s friend beating him + my father\'s friend slap him + my father\'s friend slaps him + my father\'s friend slapping him + my father\'s friend slapped him + my father\'s friend grab him + my father\'s friend grabs him + my father\'s friend grabbing him + my father\'s friend grabbed him + my father\'s friend push him + my father\'s friend pushes him + my father\'s friend pushing him + my father\'s friend pushed him + my father\'s friend harm him + my father\'s friend harms him + my father\'s friend harming him + my father\'s friend harmed him + my father\'s friend punch him + my father\'s friend punches him + my father\'s friend punching him + my father\'s friend punched him + my father\'s friend choke him + my father\'s friend chokes him + my father\'s friend choking him + my father\'s friend choked him + my father\'s friend strangle him + my father\'s friend strangles him + my father\'s friend strangling him + my father\'s friend strangled him + my father\'s friend slam him + my father\'s friend slams him + my father\'s friend slamming him + my father\'s friend slammed him + my father\'s friend point gun at him + my father\'s friend points gun at him + my father\'s friend pointing gun at him + my father\'s friend pointed gun at him

**Witnessed domestic violence search terms**

**Emotional Abuse**

my mom hate my dad + my mom hates my dad + my mom hating my dad + my mom shout at my dad + my mom shouts at my dad + my mom shouting at my dad + my mom shouted at my dad + my mom yell at my dad + my mom yells at my dad + my mom yelling at my dad + my mom yelled at my dad + my mom insult my dad + my mom insults my dad + my mom insulting my dad + my mom insulted my dad + my mom swear at my dad + my mom swears at my dad + my mom swearing at my dad + my mom swore at my dad + my mom threaten my dad + my mom threatens my dad + my mom threatening my dad + my mom threatened my dad + my mom put my dad down + my mom puts my dad down + my mom putting my dad down + my mother hate my father + my mother hates my father + my mother hating my father + my mother shout at my father + my mother shouts at my father + my mother shouting at my father + my mother shouted at my father + my mother yell at my father + my mother yells at my father + my mother yelling at my father + my mother yelled at my father + my mother insult my father + my mother insults my father + my mother insulting my father + my mother insulted my father + my mother swear at my father + my mother swears at my father + my mother swearing at my father + my mother swore at my father + my mother threaten my father + my mother threatens my father + my mother threatening my father + my mother threatened my father + my mother put my father down + my mother puts my father down + my mother putting my father down + my mom hate my step dad + my mom hates my step dad + my mom hating my step dad + my mom shout at my step dad + my mom shouts at my step dad + my mom shouting at my step dad + my mom shouted at my step dad + my mom yell at my step dad + my mom yells at my step dad + my mom yelling at my step dad + my mom yelled at my step dad + my mom insult my step dad + my mom insults my step dad + my mom insulting my step dad + my mom insulted my step dad + my mom swear at my step dad + my mom swears at my step dad + my mom swearing at my step dad + my mom swore at my step dad + my mom threaten my step dad + my mom threatens my step dad + my mom threatening my step dad + my mom threatened my step dad + my mom put my step dad down + my mom puts my step dad down + my mom putting my step dad down + my mother hate my step father + my mother hates my step father + my mother hating my step father + my mother shout at my step father + my mother shouts at my step father + my mother shouting at my step father + my mother shouted at my step father + my mother yell at my step father + my mother yells at my step father + my mother yelling at my step father + my mother yelled at my step father + my mother insult my step father + my mother insults my step father + my mother insulting my step father + my mother insulted my step father + my mother swear at my step father + my mother swears at my step father + my mother swearing at my step father + my mother swore at my step father + my mother threaten my step father + my mother threatens my step father + my mother threatening my step father + my mother threatened my step father + my mother put my step father down + my mother puts my step father down + my mother putting my step father down + my mom hate her boyfriend + my mom hates her boyfriend + my mom hating her boyfriend + my mom shout at her boyfriend + my mom shouts at her boyfriend + my mom shouting at her boyfriend + my mom shouted at her boyfriend + my mom yell at her boyfriend + my mom yells at her boyfriend + my mom yelling at her boyfriend + my mom yelled at her boyfriend + my mom insult her boyfriend + my mom insults her boyfriend + my mom insulting her boyfriend + my mom insulted her boyfriend + my mom swear at her boyfriend + my mom swears at her boyfriend + my mom swearing at her boyfriend + my mom swore at her boyfriend + my mom threaten her boyfriend + my mom threatens her boyfriend + my mom threatening her boyfriend + my mom threatened her boyfriend + my mom put her boyfriend down + my mom puts her boyfriend down + my mom putting her boyfriend down + my mother hate her boyfriend + my mother hates her boyfriend + my mother hating her boyfriend + my mother shout at her boyfriend + my mother shouts at her boyfriend + my mother shouting at her boyfriend + my mother shouted at her boyfriend + my mother yell at her boyfriend + my mother yells at her boyfriend + my mother yelling at her boyfriend + my mother yelled at her boyfriend + my mother insult her boyfriend + my mother insults her boyfriend + my mother insulting her boyfriend + my mother insulted her boyfriend + my mother swear at her boyfriend + my mother swears at her boyfriend + my mother swearing at her boyfriend + my mother swore at her boyfriend + my mother threaten her boyfriend + my mother threatens her boyfriend + my mother threatening her boyfriend + my mother threatened her boyfriend + my mother put her boyfriend down + my mother puts her boyfriend down + my mother putting her boyfriend down + my mom\'s boyfriend hate her + my mom\'s boyfriend hates her + my mom\'s boyfriend hating her + my mom\'s boyfriend shout at her + my mom\'s boyfriend shouts at her + my mom\'s boyfriend shouting at her + my mom\'s boyfriend shouted at her + my mom\'s boyfriend yell at her + my mom\'s boyfriend yells at her + my mom\'s boyfriend yelling at her + my mom\'s boyfriend yelled at her + my mom\'s boyfriend insult her + my mom\'s boyfriend insults her + my mom\'s boyfriend insulting her + my mom\'s boyfriend insulted her + my mom\'s boyfriend swear at her + my mom\'s boyfriend swears at her + my mom\'s boyfriend swearing at her + my mom\'s boyfriend swore at her + my mom\'s boyfriend threaten her + my mom\'s boyfriend threatens her + my mom\'s boyfriend threatening her + my mom\'s boyfriend threatened her + my mom\'s boyfriend put her down + my mom\'s boyfriend puts her down + my mom\'s boyfriend putting her down + my mother\'s boyfriend hate her + my mother\'s boyfriend hates her + my mother\'s boyfriend hating her + my mother\'s boyfriend shout at her + my mother\'s boyfriend shouts at her + my mother\'s boyfriend shouting at her + my mother\'s boyfriend shouted at her + my mother\'s boyfriend yell at her + my mother\'s boyfriend yells at her + my mother\'s boyfriend yelling at her + my mother\'s boyfriend yelled at her + my mother\'s boyfriend insult her + my mother\'s boyfriend insults her + my mother\'s boyfriend insulting her + my mother\'s boyfriend insulted her + my mother\'s boyfriend swear at her + my mother\'s boyfriend swears at her + my mother\'s boyfriend swearing at her + my mother\'s boyfriend swore at her + my mother\'s boyfriend threaten her + my mother\'s boyfriend threatens her + my mother\'s boyfriend threatening her + my mother\'s boyfriend threatened her + my mother\'s boyfriend put her down + my mother\'s boyfriend puts her down + my mother\'s boyfriend putting her down + my mom hate her girlfriend + my mom hates her girlfriend + my mom hating her girlfriend + my mom shout at her girlfriend + my mom shouts at her girlfriend + my mom shouting at her girlfriend + my mom shouted at her girlfriend + my mom yell at her girlfriend + my mom yells at her girlfriend + my mom yelling at her girlfriend + my mom yelled at her girlfriend + my mom insult her girlfriend + my mom insults her girlfriend + my mom insulting her girlfriend + my mom insulted her girlfriend + my mom swear at her girlfriend + my mom swears at her girlfriend + my mom swearing at her girlfriend + my mom swore at her girlfriend + my mom threaten her girlfriend + my mom threatens her girlfriend + my mom threatening her girlfriend + my mom threatened her girlfriend + my mom put her girlfriend down + my mom puts her girlfriend down + my mom putting her girlfriend down + my mother hate her girlfriend + my mother hates her girlfriend + my mother hating her girlfriend + my mother shout at her girlfriend + my mother shouts at her girlfriend + my mother shouting at her girlfriend + my mother shouted at her girlfriend + my mother yell at her girlfriend + my mother yells at her girlfriend + my mother yelling at her girlfriend + my mother yelled at her girlfriend + my mother insult her girlfriend + my mother insults her girlfriend + my mother insulting her girlfriend + my mother insulted her girlfriend + my mother swear at her girlfriend + my mother swears at her girlfriend + my mother swearing at her girlfriend + my mother swore at her girlfriend + my mother threaten her girlfriend + my mother threatens her girlfriend + my mother threatening her girlfriend + my mother threatened her girlfriend + my mother put her girlfriend down + my mother puts her girlfriend down + my mother putting her girlfriend down + my mom\'s girlfriend hate her + my mom\'s girlfriend hates her + my mom\'s girlfriend hating her + my mom\'s girlfriend shout at her + my mom\'s girlfriend shouts at her + my mom\'s girlfriend shouting at her + my mom\'s girlfriend shouted at her + my mom\'s girlfriend yell at her + my mom\'s girlfriend yells at her + my mom\'s girlfriend yelling at her + my mom\'s girlfriend yelled at her + my mom\'s girlfriend insult her + my mom\'s girlfriend insults her + my mom\'s girlfriend insulting her + my mom\'s girlfriend insulted her + my mom\'s girlfriend swear at her + my mom\'s girlfriend swears at her + my mom\'s girlfriend swearing at her + my mom\'s girlfriend swore at her + my mom\'s girlfriend threaten her + my mom\'s girlfriend threatens her + my mom\'s girlfriend threatening her + my mom\'s girlfriend threatened her + my mom\'s girlfriend put her down + my mom\'s girlfriend puts her down + my mom\'s girlfriend putting her down + my mother\'s girlfriend hate her + my mother\'s girlfriend hates her + my mother\'s girlfriend hating her + my mother\'s girlfriend shout at her + my mother\'s girlfriend shouts at her + my mother\'s girlfriend shouting at her + my mother\'s girlfriend shouted at her + my mother\'s girlfriend yell at her + my mother\'s girlfriend yells at her + my mother\'s girlfriend yelling at her + my mother\'s girlfriend yelled at her + my mother\'s girlfriend insult her + my mother\'s girlfriend insults her + my mother\'s girlfriend insulting her + my mother\'s girlfriend insulted her + my mother\'s girlfriend swear at her + my mother\'s girlfriend swears at her + my mother\'s girlfriend swearing at her + my mother\'s girlfriend swore at her + my mother\'s girlfriend threaten her + my mother\'s girlfriend threatens her + my mother\'s girlfriend threatening her + my mother\'s girlfriend threatened her + my mother\'s girlfriend put her down + my mother\'s girlfriend puts her down + my mother\'s girlfriend putting her down + my mom hate my mom + my mom hates my mom + my mom hating my mom + my mom shout at my mom + my mom shouts at my mom + my mom shouting at my mom + my mom shouted at my mom + my mom yell at my mom + my mom yells at my mom + my mom yelling at my mom + my mom yelled at my mom + my mom insult my mom + my mom insults my mom + my mom insulting my mom + my mom insulted my mom + my mom swear at my mom + my mom swears at my mom + my mom swearing at my mom + my mom swore at my mom + my mom threaten my mom + my mom threatens my mom + my mom threatening my mom + my mom threatened my mom + my mom put my mom down + my mom puts my mom down + my mom putting my mom down + my mom hate my mother + my mom hates my mother + my mom hating my mother + my mom shout at my mother + my mom shouts at my mother + my mom shouting at my mother + my mom shouted at my mother + my mom yell at my mother + my mom yells at my mother + my mom yelling at my mother + my mom yelled at my mother + my mom insult my mother + my mom insults my mother + my mom insulting my mother + my mom insulted my mother + my mom swear at my mother + my mom swears at my mother + my mom swearing at my mother + my mom swore at my mother + my mom threaten my mother + my mom threatens my mother + my mom threatening my mother + my mom threatened my mother + my mom put my mother down + my mom puts my mother down + my mom putting my mother down + my mother hate my mother + my mother hates my mother + my mother hating my mother + my mother shout at my mother + my mother shouts at my mother + my mother shouting at my mother + my mother shouted at my mother + my mother yell at my mother + my mother yells at my mother + my mother yelling at my mother + my mother yelled at my mother + my mother insult my mother + my mother insults my mother + my mother insulting my mother + my mother insulted my mother + my mother swear at my mother + my mother swears at my mother + my mother swearing at my mother + my mother swore at my mother + my mother threaten my mother + my mother threatens my mother + my mother threatening my mother + my mother threatened my mother + my mother put my mother down + my mother puts my mother down + my mother putting my mother down + my dad hate my step mom + my dad hates my step mom + my dad hating my step mom + my dad shout at my step mom + my dad shouts at my step mom + my dad shouting at my step mom + my dad shouted at my step mom + my dad yell at my step mom + my dad yells at my step mom + my dad yelling at my step mom + my dad yelled at my step mom + my dad insult my step mom + my dad insults my step mom + my dad insulting my step mom + my dad insulted my step mom + my dad swear at my step mom + my dad swears at my step mom + my dad swearing at my step mom + my dad swore at my step mom + my dad threaten my step mom + my dad threatens my step mom + my dad threatening my step mom + my dad threatened my step mom + my dad put my step mom down + my dad puts my step mom down + my dad putting my step mom down + my father hate my step mother + my father hates my step mother + my father hating my step mother + my father shout at my step mother + my father shouts at my step mother + my father shouting at my step mother + my father shouted at my step mother + my father yell at my step mother + my father yells at my step mother + my father yelling at my step mother + my father yelled at my step mother + my father insult my step mother + my father insults my step mother + my father insulting my step mother + my father insulted my step mother + my father swear at my step mother + my father swears at my step mother + my father swearing at my step mother + my father swore at my step mother + my father threaten my step mother + my father threatens my step mother + my father threatening my step mother + my father threatened my step mother + my father put my step mother down + my father puts my step mother down + my father putting my step mother down + my dad hate his girlfriend + my dad hates his girlfriend + my dad hating his girlfriend + my dad shout at his girlfriend + my dad shouts at his girlfriend + my dad shouting at his girlfriend + my dad shouted at his girlfriend + my dad yell at his girlfriend + my dad yells at his girlfriend + my dad yelling at his girlfriend + my dad yelled at his girlfriend + my dad insult his girlfriend + my dad insults his girlfriend + my dad insulting his girlfriend + my dad insulted his girlfriend + my dad swear at his girlfriend + my dad swears at his girlfriend + my dad swearing at his girlfriend + my dad swore at his girlfriend + my dad threaten his girlfriend + my dad threatens his girlfriend + my dad threatening his girlfriend + my dad threatened his girlfriend + my dad put his girlfriend down + my dad puts his girlfriend down + my dad putting his girlfriend down + my father hate his girlfriend + my father hates his girlfriend + my father hating his girlfriend + my father shout at his girlfriend + my father shouts at his girlfriend + my father shouting at his girlfriend + my father shouted at his girlfriend + my father yell at his girlfriend + my father yells at his girlfriend + my father yelling at his girlfriend + my father yelled at his girlfriend + my father insult his girlfriend + my father insults his girlfriend + my father insulting his girlfriend + my father insulted his girlfriend + my father swear at his girlfriend + my father swears at his girlfriend + my father swearing at his girlfriend + my father swore at his girlfriend + my father threaten his girlfriend + my father threatens his girlfriend + my father threatening his girlfriend + my father threatened his girlfriend + my father put his girlfriend down + my father puts his girlfriend down + my father putting his girlfriend down + my dad\'s girlfriend hate him + my dad\'s girlfriend hates him + my dad\'s girlfriend hating him + my dad\'s girlfriend shout at him + my dad\'s girlfriend shouts at him + my dad\'s girlfriend shouting at him + my dad\'s girlfriend shouted at him + my dad\'s girlfriend yell at him + my dad\'s girlfriend yells at him + my dad\'s girlfriend yelling at him + my dad\'s girlfriend yelled at him + my dad\'s girlfriend insult him + my dad\'s girlfriend insults him + my dad\'s girlfriend insulting him + my dad\'s girlfriend insulted him + my dad\'s girlfriend swear at him + my dad\'s girlfriend swears at him + my dad\'s girlfriend swearing at him + my dad\'s girlfriend swore at him + my dad\'s girlfriend threaten him + my dad\'s girlfriend threatens him + my dad\'s girlfriend threatening him + my dad\'s girlfriend threatened him + my dad\'s girlfriend put him down + my dad\'s girlfriend puts him down + my dad\'s girlfriend putting him down + my father\'s girlfriend hate him + my father\'s girlfriend hates him + my father\'s girlfriend hating him + my father\'s girlfriend shout at him + my father\'s girlfriend shouts at him + my father\'s girlfriend shouting at him + my father\'s girlfriend shouted at him + my father\'s girlfriend yell at him + my father\'s girlfriend yells at him + my father\'s girlfriend yelling at him + my father\'s girlfriend yelled at him + my father\'s girlfriend insult him + my father\'s girlfriend insults him + my father\'s girlfriend insulting him + my father\'s girlfriend insulted him + my father\'s girlfriend swear at him + my father\'s girlfriend swears at him + my father\'s girlfriend swearing at him + my father\'s girlfriend swore at him + my father\'s girlfriend threaten him + my father\'s girlfriend threatens him + my father\'s girlfriend threatening him + my father\'s girlfriend threatened him + my father\'s girlfriend put him down + my father\'s girlfriend puts him down + my father\'s girlfriend putting him down + my dad hate his boyfriend + my dad hates his boyfriend + my dad hating his boyfriend + my dad shout at his boyfriend + my dad shouts at his boyfriend + my dad shouting at his boyfriend + my dad shouted at his boyfriend + my dad yell at his boyfriend + my dad yells at his boyfriend + my dad yelling at his boyfriend + my dad yelled at his boyfriend + my dad insult his boyfriend + my dad insults his boyfriend + my dad insulting his boyfriend + my dad insulted his boyfriend + my dad swear at his boyfriend + my dad swears at his boyfriend + my dad swearing at his boyfriend + my dad swore at his boyfriend + my dad threaten his boyfriend + my dad threatens his boyfriend + my dad threatening his boyfriend + my dad threatened his boyfriend + my dad put his boyfriend down + my dad puts his boyfriend down + my dad putting his boyfriend down + my father hate his boyfriend + my father hates his boyfriend + my father hating his boyfriend + my father shout at his boyfriend + my father shouts at his boyfriend + my father shouting at his boyfriend + my father shouted at his boyfriend + my father yell at his boyfriend + my father yells at his boyfriend + my father yelling at his boyfriend + my father yelled at his boyfriend + my father insult his boyfriend + my father insults his boyfriend + my father insulting his boyfriend + my father insulted his boyfriend + my father swear at his boyfriend + my father swears at his boyfriend + my father swearing at his boyfriend + my father swore at his boyfriend + my father threaten his boyfriend + my father threatens his boyfriend + my father threatening his boyfriend + my father threatened his boyfriend + my father put his boyfriend down + my father puts his boyfriend down + my father putting his boyfriend down + my dad\'s boyfriend hate him + my dad\'s boyfriend hates him + my dad\'s boyfriend hating him + my dad\'s boyfriend shout at him + my dad\'s boyfriend shouts at him + my dad\'s boyfriend shouting at him + my dad\'s boyfriend shouted at him + my dad\'s boyfriend yell at him + my dad\'s boyfriend yells at him + my dad\'s boyfriend yelling at him + my dad\'s boyfriend yelled at him + my dad\'s boyfriend insult him + my dad\'s boyfriend insults him + my dad\'s boyfriend insulting him + my dad\'s boyfriend insulted him + my dad\'s boyfriend swear at him + my dad\'s boyfriend swears at him + my dad\'s boyfriend swearing at him + my dad\'s boyfriend swore at him + my dad\'s boyfriend threaten him + my dad\'s boyfriend threatens him + my dad\'s boyfriend threatening him + my dad\'s boyfriend threatened him + my dad\'s boyfriend put him down + my dad\'s boyfriend puts him down + my dad\'s boyfriend putting him down + my father\'s boyfriend hate him + my father\'s boyfriend hates him + my father\'s boyfriend hating him + my father\'s boyfriend shout at him + my father\'s boyfriend shouts at him + my father\'s boyfriend shouting at him + my father\'s boyfriend shouted at him + my father\'s boyfriend yell at him + my father\'s boyfriend yells at him + my father\'s boyfriend yelling at him + my father\'s boyfriend yelled at him + my father\'s boyfriend insult him + my father\'s boyfriend insults him + my father\'s boyfriend insulting him + my father\'s boyfriend insulted him + my father\'s boyfriend swear at him + my father\'s boyfriend swears at him + my father\'s boyfriend swearing at him + my father\'s boyfriend swore at him + my father\'s boyfriend threaten him + my father\'s boyfriend threatens him + my father\'s boyfriend threatening him + my father\'s boyfriend threatened him + my father\'s boyfriend put him down + my father\'s boyfriend puts him down + my father\'s boyfriend putting him down + my dad hate my dad + my dad hates my dad + my dad hating my dad + my dad shout at my dad + my dad shouts at my dad + my dad shouting at my dad + my dad shouted at my dad + my dad yell at my dad + my dad yells at my dad + my dad yelling at my dad + my dad yelled at my dad + my dad insult my dad + my dad insults my dad + my dad insulting my dad + my dad insulted my dad + my dad swear at my dad + my dad swears at my dad + my dad swearing at my dad + my dad swore at my dad + my dad threaten my dad + my dad threatens my dad + my dad threatening my dad + my dad threatened my dad + my dad put my dad down + my dad puts my dad down + my dad putting my dad down + my dad hate my father + my dad hates my father + my dad hating my father + my dad shout at my father + my dad shouts at my father + my dad shouting at my father + my dad shouted at my father + my dad yell at my father + my dad yells at my father + my dad yelling at my father + my dad yelled at my father + my dad insult my father + my dad insults my father + my dad insulting my father + my dad insulted my father + my dad swear at my father + my dad swears at my father + my dad swearing at my father + my dad swore at my father + my dad threaten my father + my dad threatens my father + my dad threatening my father + my dad threatened my father + my dad put my father down + my dad puts my father down + my dad putting my father down + my father hate my father + my father hates my father + my father hating my father + my father shout at my father + my father shouts at my father + my father shouting at my father + my father shouted at my father + my father yell at my father + my father yells at my father + my father yelling at my father + my father yelled at my father + my father insult my father + my father insults my father + my father insulting my father + my father insulted my father + my father swear at my father + my father swears at my father + my father swearing at my father + my father swore at my father + my father threaten my father + my father threatens my father + my father threatening my father + my father threatened my father + my father put my father down + my father puts my father down + my father putting my father down + my mom\'s friend hate her + my mom\'s friend hates her + my mom\'s friend hating her + my mom\'s friend shout at her + my mom\'s friend shouts at her + my mom\'s friend shouting at her + my mom\'s friend shouted at her + my mom\'s friend yell at her + my mom\'s friend yells at her + my mom\'s friend yelling at her + my mom\'s friend yelled at her + my mom\'s friend insult her + my mom\'s friend insults her + my mom\'s friend insulting her + my mom\'s friend insulted her + my mom\'s friend swear at her + my mom\'s friend swears at her + my mom\'s friend swearing at her + my mom\'s friend swore at her + my mom\'s friend threaten her + my mom\'s friend threatens her + my mom\'s friend threatening her + my mom\'s friend threatened her + my mom\'s friend put her down + my mom\'s friend puts her down + my mom\'s friend putting her down + my mother\'s friend hate her + my mother\'s friend hates her + my mother\'s friend hating her + my mother\'s friend shout at her + my mother\'s friend shouts at her + my mother\'s friend shouting at her + my mother\'s friend shouted at her + my mother\'s friend yell at her + my mother\'s friend yells at her + my mother\'s friend yelling at her + my mother\'s friend yelled at her + my mother\'s friend insult her + my mother\'s friend insults her + my mother\'s friend insulting her + my mother\'s friend insulted her + my mother\'s friend swear at her + my mother\'s friend swears at her + my mother\'s friend swearing at her + my mother\'s friend swore at her + my mother\'s friend threaten her + my mother\'s friend threatens her + my mother\'s friend threatening her + my mother\'s friend threatened her + my mother\'s friend put her down + my mother\'s friend puts her down + my mother\'s friend putting her down + my dad hate his friend + my dad hates his friend + my dad hating his friend + my dad shout at his friend + my dad shouts at his friend + my dad shouting at his friend + my dad shouted at his friend + my dad yell at his friend + my dad yells at his friend + my dad yelling at his friend + my dad yelled at his friend + my dad insult his friend + my dad insults his friend + my dad insulting his friend + my dad insulted his friend + my dad swear at his friend + my dad swears at his friend + my dad swearing at his friend + my dad swore at his friend + my dad threaten his friend + my dad threatens his friend + my dad threatening his friend + my dad threatened his friend + my dad put his friend down + my dad puts his friend down + my dad putting his friend down + my father hate his friend + my father hates his friend + my father hating his friend + my father shout at his friend + my father shouts at his friend + my father shouting at his friend + my father shouted at his friend + my father yell at his friend + my father yells at his friend + my father yelling at his friend + my father yelled at his friend + my father insult his friend + my father insults his friend + my father insulting his friend + my father insulted his friend + my father swear at his friend + my father swears at his friend + my father swearing at his friend + my father swore at his friend + my father threaten his friend + my father threatens his friend + my father threatening his friend + my father threatened his friend + my father put his friend down + my father puts his friend down + my father putting his friend down + my dad\'s friend hate him + my dad\'s friend hates him + my dad\'s friend hating him + my dad\'s friend shout at him + my dad\'s friend shouts at him + my dad\'s friend shouting at him + my dad\'s friend shouted at him + my dad\'s friend yell at him + my dad\'s friend yells at him + my dad\'s friend yelling at him + my dad\'s friend yelled at him + my dad\'s friend insult him + my dad\'s friend insults him + my dad\'s friend insulting him + my dad\'s friend insulted him + my dad\'s friend swear at him + my dad\'s friend swears at him + my dad\'s friend swearing at him + my dad\'s friend swore at him + my dad\'s friend threaten him + my dad\'s friend threatens him + my dad\'s friend threatening him + my dad\'s friend threatened him + my dad\'s friend put him down + my dad\'s friend puts him down + my dad\'s friend putting him down + my father\'s friend hate him + my father\'s friend hates him + my father\'s friend hating him + my father\'s friend shout at him + my father\'s friend shouts at him + my father\'s friend shouting at him + my father\'s friend shouted at him + my father\'s friend yell at him + my father\'s friend yells at him + my father\'s friend yelling at him + my father\'s friend yelled at him + my father\'s friend insult him + my father\'s friend insults him + my father\'s friend insulting him + my father\'s friend insulted him + my father\'s friend swear at him + my father\'s friend swears at him + my father\'s friend swearing at him + my father\'s friend swore at him + my father\'s friend threaten him + my father\'s friend threatens him + my father\'s friend threatening him + my father\'s friend threatened him + my father\'s friend put him down + my father\'s friend puts him down + my father\'s friend putting him down

**Child-witnessed intimate partner violence search terms**

**Other Searches**

my mom is abusive towards my dad + my mom abuse my dad + my mom abuses my dad + my mom abusing my dad + my mom abused my dad + my mom assault my dad + my mom assaults my dad + my mom assaulting my dad + my mom assaulted my dad + my mom harass my dad + my mom harasses my dad + my mom harassing my dad + my mom harassed my dad + my mother is abusive towards my father + my mother abuse my father + my mother abuses my father + my mother abusing my father + my mother abused my father + my mother assault my father + my mother assaults my father + my mother assaulting my father + my mother assaulted my father + my mother harass my father + my mother harasses my father + my mother harassing my father + my mother harassed my father + my mom is abusive towards my step dad + my mom abuse my step dad + my mom abuses my step dad + my mom abusing my step dad + my mom abused my step dad + my mom assault my step dad + my mom assaults my step dad + my mom assaulting my step dad + my mom assaulted my step dad + my mom harass my step dad + my mom harasses my step dad + my mom harassing my step dad + my mom harassed my step dad + my mother is abusive towards my step father + my mother abuse my step father + my mother abuses my step father + my mother abusing my step father + my mother abused my step father + my mother assault my step father + my mother assaults my step father + my mother assaulting my step father + my mother assaulted my step father + my mother harass my step father + my mother harasses my step father + my mother harassing my step father + my mother harassed my step father + my mom is abusive towards her boyfriend + my mom abuse her boyfriend + my mom abuses her boyfriend + my mom abusing her boyfriend + my mom abused her boyfriend + my mom assault her boyfriend + my mom assaults her boyfriend + my mom assaulting her boyfriend + my mom assaulted her boyfriend + my mom harass her boyfriend + my mom harasses her boyfriend + my mom harassing her boyfriend + my mom harassed her boyfriend + my mother is abusive towards her boyfriend + my mother abuse her boyfriend + my mother abuses her boyfriend + my mother abusing her boyfriend + my mother abused her boyfriend + my mother assault her boyfriend + my mother assaults her boyfriend + my mother assaulting her boyfriend + my mother assaulted her boyfriend + my mother harass her boyfriend + my mother harasses her boyfriend + my mother harassing her boyfriend + my mother harassed her boyfriend + my mom\'s boyfriend is abusive towards her + my mom\'s boyfriend abuse her + my mom\'s boyfriend abuses her + my mom\'s boyfriend abusing her + my mom\'s boyfriend abused her + my mom\'s boyfriend assault her + my mom\'s boyfriend assaults her + my mom\'s boyfriend assaulting her + my mom\'s boyfriend assaulted her + my mom\'s boyfriend harass her + my mom\'s boyfriend harasses her + my mom\'s boyfriend harassing her + my mom\'s boyfriend harassed her + my mother\'s boyfriend is abusive towards her + my mother\'s boyfriend abuse her + my mother\'s boyfriend abuses her + my mother\'s boyfriend abusing her + my mother\'s boyfriend abused her + my mother\'s boyfriend assault her + my mother\'s boyfriend assaults her + my mother\'s boyfriend assaulting her + my mother\'s boyfriend assaulted her + my mother\'s boyfriend harass her + my mother\'s boyfriend harasses her + my mother\'s boyfriend harassing her + my mother\'s boyfriend harassed her + my mom is abusive towards her girlfriend + my mom abuse her girlfriend + my mom abuses her girlfriend + my mom abusing her girlfriend + my mom abused her girlfriend + my mom assault her girlfriend + my mom assaults her girlfriend + my mom assaulting her girlfriend + my mom assaulted her girlfriend + my mom harass her girlfriend + my mom harasses her girlfriend + my mom harassing her girlfriend + my mom harassed her girlfriend + my mother is abusive towards her girlfriend + my mother abuse her girlfriend + my mother abuses her girlfriend + my mother abusing her girlfriend + my mother abused her girlfriend + my mother assault her girlfriend + my mother assaults her girlfriend + my mother assaulting her girlfriend + my mother assaulted her girlfriend + my mother harass her girlfriend + my mother harasses her girlfriend + my mother harassing her girlfriend + my mother harassed her girlfriend + my mom\'s girlfriend is abusive towards her + my mom\'s girlfriend abuse her + my mom\'s girlfriend abuses her + my mom\'s girlfriend abusing her + my mom\'s girlfriend abused her + my mom\'s girlfriend assault her + my mom\'s girlfriend assaults her + my mom\'s girlfriend assaulting her + my mom\'s girlfriend assaulted her + my mom\'s girlfriend harass her + my mom\'s girlfriend harasses her + my mom\'s girlfriend harassing her + my mom\'s girlfriend harassed her + my mother\'s girlfriend is abusive towards her + my mother\'s girlfriend abuse her + my mother\'s girlfriend abuses her + my mother\'s girlfriend abusing her + my mother\'s girlfriend abused her + my mother\'s girlfriend assault her + my mother\'s girlfriend assaults her + my mother\'s girlfriend assaulting her + my mother\'s girlfriend assaulted her + my mother\'s girlfriend harass her + my mother\'s girlfriend harasses her + my mother\'s girlfriend harassing her + my mother\'s girlfriend harassed her + my mom is abusive towards my mom + my mom abuse my mom + my mom abuses my mom + my mom abusing my mom + my mom abused my mom + my mom assault my mom + my mom assaults my mom + my mom assaulting my mom + my mom assaulted my mom + my mom harass my mom + my mom harasses my mom + my mom harassing my mom + my mom harassed my mom + my mom is abusive towards my mother + my mom abuse my mother + my mom abuses my mother + my mom abusing my mother + my mom abused my mother + my mom assault my mother + my mom assaults my mother + my mom assaulting my mother + my mom assaulted my mother + my mom harass my mother + my mom harasses my mother + my mom harassing my mother + my mom harassed my mother + my mother is abusive towards my mother + my mother abuse my mother + my mother abuses my mother + my mother abusing my mother + my mother abused my mother + my mother assault my mother + my mother assaults my mother + my mother assaulting my mother + my mother assaulted my mother + my mother harass my mother + my mother harasses my mother + my mother harassing my mother + my mother harassed my mother + my dad is abusive towards my step mom + my dad abuse my step mom + my dad abuses my step mom + my dad abusing my step mom + my dad abused my step mom + my dad assault my step mom + my dad assaults my step mom + my dad assaulting my step mom + my dad assaulted my step mom + my dad harass my step mom + my dad harasses my step mom + my dad harassing my step mom + my dad harassed my step mom + my father is abusive towards my step mother + my father abuse my step mother + my father abuses my step mother + my father abusing my step mother + my father abused my step mother + my father assault my step mother + my father assaults my step mother + my father assaulting my step mother + my father assaulted my step mother + my father harass my step mother + my father harasses my step mother + my father harassing my step mother + my father harassed my step mother + my dad is abusive towards his girlfriend + my dad abuse his girlfriend + my dad abuses his girlfriend + my dad abusing his girlfriend + my dad abused his girlfriend + my dad assault his girlfriend + my dad assaults his girlfriend + my dad assaulting his girlfriend + my dad assaulted his girlfriend + my dad harass his girlfriend + my dad harasses his girlfriend + my dad harassing his girlfriend + my dad harassed his girlfriend + my father is abusive towards his girlfriend + my father abuse his girlfriend + my father abuses his girlfriend + my father abusing his girlfriend + my father abused his girlfriend + my father assault his girlfriend + my father assaults his girlfriend + my father assaulting his girlfriend + my father assaulted his girlfriend + my father harass his girlfriend + my father harasses his girlfriend + my father harassing his girlfriend + my father harassed his girlfriend + my dad\'s girlfriend is abusive towards him + my dad\'s girlfriend abuse him + my dad\'s girlfriend abuses him + my dad\'s girlfriend abusing him + my dad\'s girlfriend abused him + my dad\'s girlfriend assault him + my dad\'s girlfriend assaults him + my dad\'s girlfriend assaulting him + my dad\'s girlfriend assaulted him + my dad\'s girlfriend harass him + my dad\'s girlfriend harasses him + my dad\'s girlfriend harassing him + my dad\'s girlfriend harassed him + my father\'s girlfriend is abusive towards him + my father\'s girlfriend abuse him + my father\'s girlfriend abuses him + my father\'s girlfriend abusing him + my father\'s girlfriend abused him + my father\'s girlfriend assault him + my father\'s girlfriend assaults him + my father\'s girlfriend assaulting him + my father\'s girlfriend assaulted him + my father\'s girlfriend harass him + my father\'s girlfriend harasses him + my father\'s girlfriend harassing him + my father\'s girlfriend harassed him + my dad is abusive towards his boyfriend + my dad abuse his boyfriend + my dad abuses his boyfriend + my dad abusing his boyfriend + my dad abused his boyfriend + my dad assault his boyfriend + my dad assaults his boyfriend + my dad assaulting his boyfriend + my dad assaulted his boyfriend + my dad harass his boyfriend + my dad harasses his boyfriend + my dad harassing his boyfriend + my dad harassed his boyfriend + my father is abusive towards his boyfriend + my father abuse his boyfriend + my father abuses his boyfriend + my father abusing his boyfriend + my father abused his boyfriend + my father assault his boyfriend + my father assaults his boyfriend + my father assaulting his boyfriend + my father assaulted his boyfriend + my father harass his boyfriend + my father harasses his boyfriend + my father harassing his boyfriend + my father harassed his boyfriend + my dad\'s boyfriend is abusive towards him + my dad\'s boyfriend abuse him + my dad\'s boyfriend abuses him + my dad\'s boyfriend abusing him + my dad\'s boyfriend abused him + my dad\'s boyfriend assault him + my dad\'s boyfriend assaults him + my dad\'s boyfriend assaulting him + my dad\'s boyfriend assaulted him + my dad\'s boyfriend harass him + my dad\'s boyfriend harasses him + my dad\'s boyfriend harassing him + my dad\'s boyfriend harassed him + my father\'s boyfriend is abusive towards him + my father\'s boyfriend abuse him + my father\'s boyfriend abuses him + my father\'s boyfriend abusing him + my father\'s boyfriend abused him + my father\'s boyfriend assault him + my father\'s boyfriend assaults him + my father\'s boyfriend assaulting him + my father\'s boyfriend assaulted him + my father\'s boyfriend harass him + my father\'s boyfriend harasses him + my father\'s boyfriend harassing him + my father\'s boyfriend harassed him + my dad is abusive towards my dad + my dad abuse my dad + my dad abuses my dad + my dad abusing my dad + my dad abused my dad + my dad assault my dad + my dad assaults my dad + my dad assaulting my dad + my dad assaulted my dad + my dad harass my dad + my dad harasses my dad + my dad harassing my dad + my dad harassed my dad + my dad is abusive towards my father + my dad abuse my father + my dad abuses my father + my dad abusing my father + my dad abused my father + my dad assault my father + my dad assaults my father + my dad assaulting my father + my dad assaulted my father + my dad harass my father + my dad harasses my father + my dad harassing my father + my dad harassed my father + my father is abusive towards my father + my father abuse my father + my father abuses my father + my father abusing my father + my father abused my father + my father assault my father + my father assaults my father + my father assaulting my father + my father assaulted my father + my father harass my father + my father harasses my father + my father harassing my father + my father harassed my father + my mom\'s friend is abusive towards her + my mom\'s friend abuse her + my mom\'s friend abuses her + my mom\'s friend abusing her + my mom\'s friend abused her + my mom\'s friend assault her + my mom\'s friend assaults her + my mom\'s friend assaulting her + my mom\'s friend assaulted her + my mom\'s friend harass her + my mom\'s friend harasses her + my mom\'s friend harassing her + my mom\'s friend harassed her + my mother\'s friend is abusive towards her + my mother\'s friend abuse her + my mother\'s friend abuses her + my mother\'s friend abusing her + my mother\'s friend abused her + my mother\'s friend assault her + my mother\'s friend assaults her + my mother\'s friend assaulting her + my mother\'s friend assaulted her + my mother\'s friend harass her + my mother\'s friend harasses her + my mother\'s friend harassing her + my mother\'s friend harassed her + my dad is abusive towards his friend + my dad abuse his friend + my dad abuses his friend + my dad abusing his friend + my dad abused his friend + my dad assault his friend + my dad assaults his friend + my dad assaulting his friend + my dad assaulted his friend + my dad harass his friend + my dad harasses his friend + my dad harassing his friend + my dad harassed his friend + my father is abusive towards his friend + my father abuse his friend + my father abuses his friend + my father abusing his friend + my father abused his friend + my father assault his friend + my father assaults his friend + my father assaulting his friend + my father assaulted his friend + my father harass his friend + my father harasses his friend + my father harassing his friend + my father harassed his friend + my dad\'s friend is abusive towards him + my dad\'s friend abuse him + my dad\'s friend abuses him + my dad\'s friend abusing him + my dad\'s friend abused him + my dad\'s friend assault him + my dad\'s friend assaults him + my dad\'s friend assaulting him + my dad\'s friend assaulted him + my dad\'s friend harass him + my dad\'s friend harasses him + my dad\'s friend harassing him + my dad\'s friend harassed him + my father\'s friend is abusive towards him + my father\'s friend abuse him + my father\'s friend abuses him + my father\'s friend abusing him + my father\'s friend abused him + my father\'s friend assault him + my father\'s friend assaults him + my father\'s friend assaulting him + my father\'s friend assaulted him + my father\'s friend harass him + my father\'s friend harasses him + my father\'s friend harassing him + my father\'s friend harassed him

**First-hand account child abuse search terms**

**Physical Abuse**

mom hit me + mother hit me + stepmom hit me + stepmother hit me + mom\'s boyfriend hit me + mom boyfriend hit me + mom\'s girlfriend hit me + mom girlfriend hit me + dad hit me + father hit me + stepdad hit me + stepfather hit me + dad\'s girlfriend hit me + dad girlfriend hit me + dad\'s boyfriend hit me + dad boyfriend hit me + grandma hit me + grandpa hit me + uncle hit me + aunt hit me + mom hits me + mother hits me + stepmom hits me + stepmother hits me + mom\'s boyfriend hits me + mom boyfriend hits me + mom\'s girlfriend hits me + mom girlfriend hits me + dad hits me + father hits me + stepdad hits me + stepfather hits me + dad\'s girlfriend hits me + dad girlfriend hits me + dad\'s boyfriend hits me + dad boyfriend hits me + grandma hits me + grandpa hits me + uncle hits me + aunt hits me + mom hurt me + mother hurt me + stepmom hurt me + stepmother hurt me + mom\'s boyfriend hurt me + mom boyfriend hurt me + mom\'s girlfriend hurt me + mom girlfriend hurt me + dad hurt me + father hurt me + stepdad hurt me + stepfather hurt me + dad\'s girlfriend hurt me + dad girlfriend hurt me + dad\'s boyfriend hurt me + dad boyfriend hurt me + grandma hurt me + grandpa hurt me + uncle hurt me + aunt hurt me + mom hurts me + mother hurts me + stepmom hurts me + stepmother hurts me + mom\'s boyfriend hurts me + mom boyfriend hurts me + mom\'s girlfriend hurts me + mom girlfriend hurts me + dad hurts me + father hurts me + stepdad hurts me + stepfather hurts me + dad\'s girlfriend hurts me + dad girlfriend hurts me + dad\'s boyfriend hurts me + dad boyfriend hurts me + grandma hurts me + grandpa hurts me + uncle hurts me + aunt hurts me + mom kick me + mother kick me + stepmom kick me + stepmother kick me + mom\'s boyfriend kick me + mom boyfriend kick me + mom\'s girlfriend kick me + mom girlfriend kick me + dad kick me + father kick me + stepdad kick me + stepfather kick me + dad\'s girlfriend kick me + dad girlfriend kick me + dad\'s boyfriend kick me + dad boyfriend kick me + grandma kick me + grandpa kick me + uncle kick me + aunt kick me + mom kicked me + mother kicked me + stepmom kicked me + stepmother kicked me + mom\'s boyfriend kicked me + mom boyfriend kicked me + mom\'s girlfriend kicked me + mom girlfriend kicked me + dad kicked me + father kicked me + stepdad kicked me + stepfather kicked me + dad\'s girlfriend kicked me + dad girlfriend kicked me + dad\'s boyfriend kicked me + dad boyfriend kicked me + grandma kicked me + grandpa kicked me + uncle kicked me + aunt kicked me + mom kicks me + mother kicks me + stepmom kicks me + stepmother kicks me + mom\'s boyfriend kicks me + mom boyfriend kicks me + mom\'s girlfriend kicks me + mom girlfriend kicks me + dad kicks me + father kicks me + stepdad kicks me + stepfather kicks me + dad\'s girlfriend kicks me + dad girlfriend kicks me + dad\'s boyfriend kicks me + dad boyfriend kicks me + grandma kicks me + grandpa kicks me + uncle kicks me + aunt kicks me + mom beat me + mother beat me + stepmom beat me + stepmother beat me + mom\'s boyfriend beat me + mom boyfriend beat me + mom\'s girlfriend beat me + mom girlfriend beat me + dad beat me + father beat me + stepdad beat me + stepfather beat me + dad\'s girlfriend beat me + dad girlfriend beat me + dad\'s boyfriend beat me + dad boyfriend beat me + grandma beat me + grandpa beat me + uncle beat me + aunt beat me + mom beats me + mother beats me + stepmom beats me + stepmother beats me + mom\'s boyfriend beats me + mom boyfriend beats me + mom\'s girlfriend beats me + mom girlfriend beats me + dad beats me + father beats me + stepdad beats me + stepfather beats me + dad\'s girlfriend beats me + dad girlfriend beats me + dad\'s boyfriend beats me + dad boyfriend beats me + grandma beats me + grandpa beats me + uncle beats me + aunt beats me + mom slap me + mother slap me + stepmom slap me + stepmother slap me + mom\'s boyfriend slap me + mom boyfriend slap me + mom\'s girlfriend slap me + mom girlfriend slap me + dad slap me + father slap me + stepdad slap me + stepfather slap me + dad\'s girlfriend slap me + dad girlfriend slap me + dad\'s boyfriend slap me + dad boyfriend slap me + grandma slap me + grandpa slap me + uncle slap me + aunt slap me + mom slaps me + mother slaps me + stepmom slaps me + stepmother slaps me + mom\'s boyfriend slaps me + mom boyfriend slaps me + mom\'s girlfriend slaps me + mom girlfriend slaps me + dad slaps me + father slaps me + stepdad slaps me + stepfather slaps me + dad\'s girlfriend slaps me + dad girlfriend slaps me + dad\'s boyfriend slaps me + dad boyfriend slaps me + grandma slaps me + grandpa slaps me + uncle slaps me + aunt slaps me + mom slapped me + mother slapped me + stepmom slapped me + stepmother slapped me + mom\'s boyfriend slapped me + mom boyfriend slapped me + mom\'s girlfriend slapped me + mom girlfriend slapped me + dad slapped me + father slapped me + stepdad slapped me + stepfather slapped me + dad\'s girlfriend slapped me + dad girlfriend slapped me + dad\'s boyfriend slapped me + dad boyfriend slapped me + grandma slapped me + grandpa slapped me + uncle slapped me + aunt slapped me + mom grab me + mother grab me + stepmom grab me + stepmother grab me + mom\'s boyfriend grab me + mom boyfriend grab me + mom\'s girlfriend grab me + mom girlfriend grab me + dad grab me + father grab me + stepdad grab me + stepfather grab me + dad\'s girlfriend grab me + dad girlfriend grab me + dad\'s boyfriend grab me + dad boyfriend grab me + grandma grab me + grandpa grab me + uncle grab me + aunt grab me + mom grabs me + mother grabs me + stepmom grabs me + stepmother grabs me + mom\'s boyfriend grabs me + mom boyfriend grabs me + mom\'s girlfriend grabs me + mom girlfriend grabs me + dad grabs me + father grabs me + stepdad grabs me + stepfather grabs me + dad\'s girlfriend grabs me + dad girlfriend grabs me + dad\'s boyfriend grabs me + dad boyfriend grabs me + grandma grabs me + grandpa grabs me + uncle grabs me + aunt grabs me + mom grabbed me + mother grabbed me + stepmom grabbed me + stepmother grabbed me + mom\'s boyfriend grabbed me + mom boyfriend grabbed me + mom\'s girlfriend grabbed me + mom girlfriend grabbed me + dad grabbed me + father grabbed me + stepdad grabbed me + stepfather grabbed me + dad\'s girlfriend grabbed me + dad girlfriend grabbed me + dad\'s boyfriend grabbed me + dad boyfriend grabbed me + grandma grabbed me + grandpa grabbed me + uncle grabbed me + aunt grabbed me + mom push me + mother push me + stepmom push me + stepmother push me + mom\'s boyfriend push me + mom boyfriend push me + mom\'s girlfriend push me + mom girlfriend push me + dad push me + father push me + stepdad push me + stepfather push me + dad\'s girlfriend push me + dad girlfriend push me + dad\'s boyfriend push me + dad boyfriend push me + grandma push me + grandpa push me + uncle push me + aunt push me + mom pushes me + mother pushes me + stepmom pushes me + stepmother pushes me + mom\'s boyfriend pushes me + mom boyfriend pushes me + mom\'s girlfriend pushes me + mom girlfriend pushes me + dad pushes me + father pushes me + stepdad pushes me + stepfather pushes me + dad\'s girlfriend pushes me + dad girlfriend pushes me + dad\'s boyfriend pushes me + dad boyfriend pushes me + grandma pushes me + grandpa pushes me + uncle pushes me + aunt pushes me + mom pushed me + mother pushed me + stepmom pushed me + stepmother pushed me + mom\'s boyfriend pushed me + mom boyfriend pushed me + mom\'s girlfriend pushed me + mom girlfriend pushed me + dad pushed me + father pushed me + stepdad pushed me + stepfather pushed me + dad\'s girlfriend pushed me + dad girlfriend pushed me + dad\'s boyfriend pushed me + dad boyfriend pushed me + grandma pushed me + grandpa pushed me + uncle pushed me + aunt pushed me + mom harm me + mother harm me + stepmom harm me + stepmother harm me + mom\'s boyfriend harm me + mom boyfriend harm me + mom\'s girlfriend harm me + mom girlfriend harm me + dad harm me + father harm me + stepdad harm me + stepfather harm me + dad\'s girlfriend harm me + dad girlfriend harm me + dad\'s boyfriend harm me + dad boyfriend harm me + grandma harm me + grandpa harm me + uncle harm me + aunt harm me + mom harms me + mother harms me + stepmom harms me + stepmother harms me + mom\'s boyfriend harms me + mom boyfriend harms me + mom\'s girlfriend harms me + mom girlfriend harms me + dad harms me + father harms me + stepdad harms me + stepfather harms me + dad\'s girlfriend harms me + dad girlfriend harms me + dad\'s boyfriend harms me + dad boyfriend harms me + grandma harms me + grandpa harms me + uncle harms me + aunt harms me + mom harmed me + mother harmed me + stepmom harmed me + stepmother harmed me + mom\'s boyfriend harmed me + mom boyfriend harmed me + mom\'s girlfriend harmed me + mom girlfriend harmed me + dad harmed me + father harmed me + stepdad harmed me + stepfather harmed me + dad\'s girlfriend harmed me + dad girlfriend harmed me + dad\'s boyfriend harmed me + dad boyfriend harmed me + grandma harmed me + grandpa harmed me + uncle harmed me + aunt harmed me + mom punch me + mother punch me + stepmom punch me + stepmother punch me + mom\'s boyfriend punch me + mom boyfriend punch me + mom\'s girlfriend punch me + mom girlfriend punch me + dad punch me + father punch me + stepdad punch me + stepfather punch me + dad\'s girlfriend punch me + dad girlfriend punch me + dad\'s boyfriend punch me + dad boyfriend punch me + grandma punch me + grandpa punch me + uncle punch me + aunt punch me + mom punches me + mother punches me + stepmom punches me + stepmother punches me + mom\'s boyfriend punches me + mom boyfriend punches me + mom\'s girlfriend punches me + mom girlfriend punches me + dad punches me + father punches me + stepdad punches me + stepfather punches me + dad\'s girlfriend punches me + dad girlfriend punches me + dad\'s boyfriend punches me + dad boyfriend punches me + grandma punches me + grandpa punches me + uncle punches me + aunt punches me + mom punched me + mother punched me + stepmom punched me + stepmother punched me + mom\'s boyfriend punched me + mom boyfriend punched me + mom\'s girlfriend punched me + mom girlfriend punched me + dad punched me + father punched me + stepdad punched me + stepfather punched me + dad\'s girlfriend punched me + dad girlfriend punched me + dad\'s boyfriend punched me + dad boyfriend punched me + dad boyfriend punched me + grandma punched me + grandpa punched me + uncle punched me + aunt punched me + babysitter hit me + nanny hit me + babysitter hits me + nanny hits me + babysitter hurt me + nanny hurt me + babysitter hurts me + nanny hurts me + babysitter kick me + nanny kick me + babysitter kicks me + nanny kicks me + babysitter kicked me + nanny kicked me + babysitter beat me + nanny beat me + babysitter beats me + nanny beats me + babysitter slap me + nanny slap me + babysitter slaps me + nanny slaps me + nanny slaps me + babysitter slapped me + nanny slapped me + babysitter grab me + nanny grab me + babysitter grabs me + nanny grabs me + babysitter grabbed me + nanny grabbed me + babysitter push me + nanny push me + babysitter pushes me + nanny pushes me + babysitter pushed me + nanny pushed me + babysitter harm me + nanny harm me + babysitter harms me + nanny harms me + babysitter harmed me + nanny harmed me + babysitter punch me + nanny punch me + babysitter punches me + nanny punches me + babysitter punched me + nanny punched me + mother\'s boyfriend hit me + mother boyfriend hit me + mother\'s girlfriend hit me + mother girlfriend hit me + father\'s boyfriend hit me + father boyfriend hit me + father\'s girlfriend hit me + father girlfriend hit me + parent hit me + parents hit me + mother\'s boyfriend hits me + mother boyfriend hits me + mother\'s girlfriend hits me + mother girlfriend hits me + father\'s boyfriend hits me + father boyfriend hits me + father\'s girlfriend hits me + father girlfriend hits me + parent hits me + parents hits me + mother\'s boyfriend hurt me + mother boyfriend hurt me + mother\'s girlfriend hurt me + mother girlfriend hurt me + father\'s boyfriend hurt me + father boyfriend hurt me + father\'s girlfriend hurt me + father girlfriend hurt me + parent hurt me + parents hurt me + mother\'s boyfriend hurts me + mother boyfriend hurts me + mother\'s girlfriend hurts me + mother girlfriend hurts me + father\'s boyfriend hurts me + father boyfriend hurts me + father\'s girlfriend hurts me + father girlfriend hurts me + parent hurts me + parents hurts me + mother\'s boyfriend kick me + mother boyfriend kick me + mother\'s girlfriend kick me + mother girlfriend kick me + father\'s boyfriend kick me + father boyfriend kick me + father\'s girlfriend kick me + father girlfriend kick me + parent kick me + parents kick me + mother\'s boyfriend kicks me + mother boyfriend kicks me + mother\'s girlfriend kicks me + mother girlfriend kicks me + father\'s boyfriend kicks me + father boyfriend kicks me + father\'s girlfriend kicks me + father girlfriend kicks me + parent kicks me + parents kicks me + mother\'s boyfriend kicked me + mother boyfriend kicked me + mother\'s girlfriend kicked me + mother girlfriend kicked me + father\'s boyfriend kicked me + father boyfriend kicked me + father\'s girlfriend kicked me + father girlfriend kicked me + parent kicked me + parents kicked me + mother\'s boyfriend beat me + mother boyfriend beat me + mother\'s girlfriend beat me + mother girlfriend beat me + father\'s boyfriend beat me + father boyfriend beat me + father\'s girlfriend beat me + father girlfriend beat me + parent beat me + parents beat me + mother\'s boyfriend beats me + mother boyfriend beats me + mother\'s girlfriend beats me + mother girlfriend beats me + father\'s boyfriend beats me + father boyfriend beats me + father\'s girlfriend beats me + father girlfriend beats me + parent beats me + parents beats me + mother\'s boyfriend slap me + mother boyfriend slap me + mother\'s girlfriend slap me + mother girlfriend slap me + father\'s boyfriend slap me + father boyfriend slap me + father\'s girlfriend slap me + father girlfriend slap me + parent slap me + parents slap me + mother\'s boyfriend slaps me + mother boyfriend slaps me + mother\'s girlfriend slaps me + mother girlfriend slaps me + father\'s boyfriend slaps me + father boyfriend slaps me + father\'s girlfriend slaps me + father girlfriend slaps me + parent slaps me + parents slaps me + mother\'s boyfriend slapped me + mother boyfriend slapped me + mother\'s girlfriend slapped me + mother girlfriend slapped me + father\'s boyfriend slapped me + father boyfriend slapped me + father\'s girlfriend slapped me + father girlfriend slapped me + parent slapped me + parents slapped me + mother\'s boyfriend grab me + mother boyfriend grab me + mother\'s girlfriend grab me + mother girlfriend grab me + father\'s boyfriend grab me + father boyfriend grab me + father\'s girlfriend grab me + father girlfriend grab me + parent grab me + parents grab me + mother\'s boyfriend grabs me + mother boyfriend grabs me + mother\'s girlfriend grabs me + mother girlfriend grabs me + father\'s boyfriend grabs me + father boyfriend grabs me + father\'s girlfriend grabs me + father girlfriend grabs me + parent grabs me + parents grabs me + mother\'s boyfriend grabbed me + mother boyfriend grabbed me + mother\'s girlfriend grabbed me + mother girlfriend grabbed me + father\'s boyfriend grabbed me + father boyfriend grabbed me + father\'s girlfriend grabbed me + father girlfriend grabbed me + parent grabbed me + parents grabbed me + mother\'s boyfriend push me + mother boyfriend push me + mother\'s girlfriend push me + mother girlfriend push me + father\'s boyfriend push me + father boyfriend push me + father\'s girlfriend push me + father girlfriend push me + parent push me + parents push me + mother\'s boyfriend pushes me + mother boyfriend pushes me + mother\'s girlfriend pushes me + mother girlfriend pushes me + father\'s boyfriend pushes me + father boyfriend pushes me + father\'s girlfriend pushes me + father girlfriend pushes me + parent pushes me + parents pushes me + mother\'s boyfriend pushed me + mother boyfriend pushed me + mother\'s girlfriend pushed me + mother girlfriend pushed me + father\'s boyfriend pushed me + father boyfriend pushed me + father\'s girlfriend pushed me + father girlfriend pushed me + parent pushed me + parents pushed me + mother\'s boyfriend harm me + mother boyfriend harm me + mother\'s girlfriend harm me + mother girlfriend harm me + father\'s boyfriend harm me + father boyfriend harm me + father\'s girlfriend harm me + father girlfriend harm me + parent harm me + parents harm me + mother\'s boyfriend harms me + mother boyfriend harms me + mother\'s girlfriend harms me + mother girlfriend harms me + father\'s boyfriend harms me + father boyfriend harms me + father\'s girlfriend harms me + father girlfriend harms me + parent harms me + parents harms me + mother\'s boyfriend harmed me + mother boyfriend harmed me + mother\'s girlfriend harmed me + mother girlfriend harmed me + father\'s boyfriend harmed me + father boyfriend harmed me + father\'s girlfriend harmed me + father girlfriend harmed me + parent harmed me + parents harmed me + mother\'s boyfriend punch me + mother boyfriend punch me + mother\'s girlfriend punch me + mother girlfriend punch me + father\'s boyfriend punch me + father boyfriend punch me + father\'s girlfriend punch me + father girlfriend punch me + parent punch me + parents punch me + mother\'s boyfriend punches me + mother boyfriend punches me + mother\'s girlfriend punches me + mother girlfriend punches me + father\'s boyfriend punches me + father boyfriend punches me + father\'s girlfriend punches me + father girlfriend punches me + parent punches me + parents punches me + mother\'s boyfriend punched me + mother boyfriend punched me + mother\'s girlfriend punched me + mother girlfriend punched me + father\'s boyfriend punched me + father boyfriend punched me + father\'s girlfriend punched me + father girlfriend punched me + parent punched me + parents punched me + mom hitting me + mother hitting me + stepmom hitting me + stepmother hitting me + mom\'s boyfriend hitting me + mom boyfriend hitting me + mom\'s girlfriend hitting me + mom girlfriend hitting me + dad hitting me + father hitting me + stepdad hitting me + stepfather hitting me + dad\'s girlfriend hitting me + dad girlfriend hitting me + dad\'s boyfriend hitting me + dad boyfriend hitting me + grandma hitting me + grandpa hitting me + uncle hitting me + aunt hitting me + mother\'s boyfriend hitting me + mother boyfriend hitting me + mother\'s girlfriend hitting me + mother girlfriend hitting me + father\'s boyfriend hitting me + father boyfriend hitting me + father\'s girlfriend hitting me + father girlfriend hitting me + parent hitting me + parents hitting me + nanny hitting me + babysitter hitting me + mom hurting me + mother hurting me + stepmom hurting me + stepmother hurting me + mom\'s boyfriend hurting me + mom boyfriend hurting me + mom\'s girlfriend hurting me + mom girlfriend hurting me + dad hurting me + father hurting me + stepdad hurting me + stepfather hurting me + dad\'s girlfriend hurting me + dad girlfriend hurting me + dad\'s boyfriend hurting me + dad boyfriend hurting me + grandma hurting me + grandpa hurting me + uncle hurting me + aunt hurting me + mother\'s boyfriend hurting me + mother boyfriend hurting me + mother\'s girlfriend hurting me + mother girlfriend hurting me + father\'s boyfriend hurting me + father boyfriend hurting me + father\'s girlfriend hurting me + father girlfriend hurting me + parent hurting me + parents hurting me + nanny hurting me + babysitter hurting me + mom kicking me + mother kicking me + stepmom kicking me + stepmother kicking me + mom\'s boyfriend kicking me + mom boyfriend kicking me + mom\'s girlfriend kicking me + mom girlfriend kicking me + dad kicking me + father kicking me + stepdad kicking me + stepfather kicking me + dad\'s girlfriend kicking me + dad girlfriend kicking me + dad\'s boyfriend kicking me + dad boyfriend kicking me + grandma kicking me + grandpa kicking me + uncle kicking me + aunt kicking me + mother\'s boyfriend kicking me + mother boyfriend kicking me + mother\'s girlfriend kicking me + mother girlfriend kicking me + father\'s boyfriend kicking me + father boyfriend kicking me + father\'s girlfriend kicking me + father girlfriend kicking me + parent kicking me + parents kicking me + nanny kicking me + babysitter kicking me + mom beating me + mother beating me + stepmom beating me + stepmother beating me + mom\'s boyfriend beating me + mom boyfriend beating me + mom\'s girlfriend beating me + mom girlfriend beating me + dad beating me + father beating me + stepdad beating me + stepfather beating me + dad\'s girlfriend beating me + dad girlfriend beating me + dad\'s boyfriend beating me + dad boyfriend beating me + grandma beating me + grandpa beating me + uncle beating me + aunt beating me + mother\'s boyfriend beating me + mother boyfriend beating me + mother\'s girlfriend beating me + mother girlfriend beating me + father\'s boyfriend beating me + father boyfriend beating me + father\'s girlfriend beating me + father girlfriend beating me + parent beating me + parents beating me + nanny beating me + babysitter beating me + mom slapping me + mother slapping me + stepmom slapping me + stepmother slapping me + mom\'s boyfriend slapping me + mom boyfriend slapping me + mom\'s girlfriend slapping me + mom girlfriend slapping me + dad slapping me + father slapping me + stepdad slapping me + stepfather slapping me + dad\'s girlfriend slapping me + dad girlfriend slapping me + dad\'s boyfriend slapping me + dad boyfriend slapping me + grandma slapping me + grandpa slapping me + uncle slapping me + aunt slapping me + mother\'s boyfriend slapping me + mother boyfriend slapping me + mother\'s girlfriend slapping me + mother girlfriend slapping me + father\'s boyfriend slapping me + father boyfriend slapping me + father\'s girlfriend slapping me + father girlfriend slapping me + parent slapping me + parents slapping me + nanny slapping me + babysitter slapping me + mom grabbing me + mother grabbing me + stepmom grabbing me + stepmother grabbing me + mom\'s boyfriend grabbing me + mom boyfriend grabbing me + mom\'s girlfriend grabbing me + mom girlfriend grabbing me + dad grabbing me + father grabbing me + stepdad grabbing me + stepfather grabbing me + dad\'s girlfriend grabbing me + dad girlfriend grabbing me + dad\'s boyfriend grabbing me + dad boyfriend grabbing me + grandma grabbing me + grandpa grabbing me + uncle grabbing me + aunt grabbing me + mother\'s boyfriend grabbing me + mother boyfriend grabbing me + mother\'s girlfriend grabbing me + mother girlfriend grabbing me + father\'s boyfriend grabbing me + father boyfriend grabbing me + father\'s girlfriend grabbing me + father girlfriend grabbing me + parent grabbing me + parents grabbing me + nanny grabbing me + babysitter grabbing me + mom pushing me + mother pushing me + stepmom pushing me + stepmother pushing me + mom\'s boyfriend pushing me + mom boyfriend pushing me + mom\'s girlfriend pushing me + mom girlfriend pushing me + dad pushing me + father pushing me + stepdad pushing me + stepfather pushing me + dad\'s girlfriend pushing me + dad girlfriend pushing me + dad\'s boyfriend pushing me + dad boyfriend pushing me + grandma pushing me + grandpa pushing me + uncle pushing me + aunt pushing me + mother\'s boyfriend pushing me + mother boyfriend pushing me + mother\'s girlfriend pushing me + mother girlfriend pushing me + father\'s boyfriend pushing me + father boyfriend pushing me + father\'s girlfriend pushing me + father girlfriend pushing me + parent pushing me + parents pushing me + nanny pushing me + babysitter pushing me + mom harming me + mother harming me + stepmom harming me + stepmother harming me + mom\'s boyfriend harming me + mom boyfriend harming me + mom\'s girlfriend harming me + mom girlfriend harming me + dad harming me + father harming me + stepdad harming me + stepfather harming me + dad\'s girlfriend harming me + dad girlfriend harming me + dad\'s boyfriend harming me + dad boyfriend harming me + grandma harming me + grandpa harming me + uncle harming me + aunt harming me + mother\'s boyfriend harming me + mother boyfriend harming me + mother\'s girlfriend harming me + mother girlfriend harming me + father\'s boyfriend harming me + father boyfriend harming me + father\'s girlfriend harming me + father girlfriend harming me + parent harming me + parents harming me + nanny harming me + babysitter harming me + mom punching me + mother punching me + stepmom punching me + stepmother punching me + mom\'s boyfriend punching me + mom boyfriend punching me + mom\'s girlfriend punching me + mom girlfriend punching me + dad punching me + father punching me + stepdad punching me + stepfather punching me + dad\'s girlfriend punching me + dad girlfriend punching me + dad\'s boyfriend punching me + dad boyfriend punching me + grandma punching me + grandpa punching me + uncle punching me + aunt punching me + mother\'s boyfriend punching me + mother boyfriend punching me + mother\'s girlfriend punching me + mother girlfriend punching me + father\'s boyfriend punching me + father boyfriend punching me + father\'s girlfriend punching me + father girlfriend punching me + parent punching me + parents punching me + nanny punching me + babysitter punching me

**First-hand account child abuse search terms**

**Emotional Abuse**

mom hates me + mother hates me + stepmom hates me + stepmother hates me + mom\'s boyfriend hates me + mom boyfriend hates me + mom\'s girlfriend hates me + mom girlfriend hates me + dad hates me + father hates me + stepdad hates me + stepfather hates me + dad\'s girlfriend hates me + dad girlfriend hates me + dad\'s boyfriend hates me + dad boyfriend hates me + grandma hates me + grandpa hates me + uncle hates me + aunt hates me + mom hate me + mother hate me + stepmom hate me + stepmother hate me + mom\'s boyfriend hate me + mom boyfriend hate me + mom\'s girlfriend hate me + mom girlfriend hate me + dad hate me + father hate me + stepdad hate me + stepfather hate me + dad\'s girlfriend hate me + dad girlfriend hate me + dad\'s boyfriend hate me + dad boyfriend hate me + grandma hate me + grandpa hate me + uncle hate me + aunt hate me + mom scare me + mother scare me + stepmom scare me + stepmother scare me + mom\'s boyfriend scare me + mom boyfriend scare me + mom\'s girlfriend scare me + mom girlfriend scare me + dad scare me + father scare me + stepdad scare me + stepfather scare me + dad\'s girlfriend scare me + dad girlfriend scare me + dad\'s boyfriend scare me + dad boyfriend scare me + grandma scare me + grandpa scare me + uncle scare me + aunt scare me + mom scares me + mother scares me + stepmom scares me + stepmother scares me + mom\'s boyfriend scares me + mom boyfriend scares me + mom\'s girlfriend scares me + mom girlfriend scares me + dad scares me + father scares me + stepdad scares me + stepfather scares me + dad\'s girlfriend scares me + dad girlfriend scares me + dad\'s boyfriend scares me + dad boyfriend scares me + grandma scares me + grandpa scares me + uncle scares me + aunt scares me + mom scared me + mother scared me + stepmom scared me + stepmother scared me + mom\'s boyfriend scared me + mom boyfriend scared me + mom\'s girlfriend scared me + mom girlfriend scared me + dad scared me + father scared me + stepdad scared me + stepfather scared me + dad\'s girlfriend scared me + dad girlfriend scared me + dad\'s boyfriend scared me + dad boyfriend scared me + grandma scared me + grandpa scared me + uncle scared me + aunt scared me + mom threaten me + mother threaten me + stepmom threaten me + stepmother threaten me + mom\'s boyfriend threaten me + mom boyfriend threaten me + mom\'s girlfriend threaten me + mom girlfriend threaten me + dad threaten me + father threaten me + stepdad threaten me + stepfather threaten me + dad\'s girlfriend threaten me + dad girlfriend threaten me + dad\'s boyfriend threaten me + dad boyfriend threaten me + grandma threaten me + grandpa threaten me + uncle threaten me + aunt threaten me + mom threatens me + mother threatens me + stepmom threatens me + stepmother threatens me + mom\'s boyfriend threatens me + mom boyfriend threatens me + mom\'s girlfriend threatens me + mom girlfriend threatens me + dad threatens me + father threatens me + stepdad threatens me + stepfather threatens me + dad\'s girlfriend threatens me + dad girlfriend threatens me + dad\'s boyfriend threatens me + dad boyfriend threatens me + grandma threatens me + grandpa threatens me + uncle threatens me + aunt threatens me + mom threatened me + mother threatened me + stepmom threatened me + stepmother threatened me + mom\'s boyfriend threatened me + mom boyfriend threatened me + mom\'s girlfriend threatened me + mom girlfriend threatened me + dad threatened me + father threatened me + stepdad threatened me + stepfather threatened me + dad\'s girlfriend threatened me + dad girlfriend threatened me + dad\'s boyfriend threatened me + dad boyfriend threatened me + grandma threatened me + grandpa threatened me + uncle threatened me + aunt threatened me + mom does not care about me + mother does not care about me + stepmom does not care about me + stepmother does not care about me + mom\'s boyfriend does not care about me + mom boyfriend does not care about me + mom\'s girlfriend does not care about me + mom girlfriend does not care about me + dad does not care about me + father does not care about me + stepdad does not care about me + stepfather does not care about me + dad\'s girlfriend does not care about me + dad girlfriend does not care about me + dad\'s boyfriend does not care about me + dad boyfriend does not care about me + grandma does not care about me + grandpa does not care about me + uncle does not care about me + aunt does not care about me + mom doesnt care about me + mother doesnt care about me + stepmom doesnt care about me + stepmother doesnt care about me + mom\'s boyfriend doesnt care about me + mom boyfriend doesnt care about me + mom\'s girlfriend doesnt care about me + mom girlfriend doesnt care about me + dad doesnt care about me + father doesnt care about me + stepdad doesnt care about me + stepfather doesnt care about me + dad\'s girlfriend doesnt care about me + dad girlfriend doesnt care about me + dad\'s boyfriend doesnt care about me + dad boyfriend doesnt care about me + grandma doesnt care about me + grandpa doesnt care about me + uncle doesnt care about me + aunt doesnt care about me + mom doesn\'t care about me + mother doesn\'t care about me + stepmom doesn\'t care about me + stepmother doesn\'t care about me + mom\'s boyfriend doesn\'t care about me + mom boyfriend doesn\'t care about me + mom\'s girlfriend doesn\'t care about me + mom girlfriend doesn\'t care about me + dad doesn\'t care about me + father doesn\'t care about me + stepdad doesn\'t care about me + stepfather doesn\'t care about me + dad\'s girlfriend doesn\'t care about me + dad girlfriend doesn\'t care about me + dad\'s boyfriend doesn\'t care about me + dad boyfriend doesn\'t care about me + grandma doesn\'t care about me + grandpa doesn\'t care about me + uncle doesn\'t care about me + aunt doesn\'t care about me + mom does not love me + mother does not love me + stepmom does not love me + stepmother does not love me + mom\'s boyfriend does not love me + mom boyfriend does not love me + mom\'s girlfriend does not love me + mom girlfriend does not love me + dad does not love me + father does not love me + stepdad does not love me + stepfather does not love me + dad\'s girlfriend does not love me + dad girlfriend does not love me + dad\'s boyfriend does not love me + dad boyfriend does not love me + grandma does not love me + grandpa does not love me + uncle does not love me + aunt does not love me + mom doesnt love me + mother doesnt love me + stepmom doesnt love me + stepmother doesnt love me + mom\'s boyfriend doesnt love me + mom boyfriend doesnt love me + mom\'s girlfriend doesnt love me + mom girlfriend doesnt love me + dad doesnt love me + father doesnt love me + stepdad doesnt love me + stepfather doesnt love me + dad\'s girlfriend doesnt love me + dad girlfriend doesnt love me + dad\'s boyfriend doesnt love me + dad boyfriend doesnt love me + grandma doesnt love me + grandpa doesnt love me + uncle doesnt love me + aunt doesnt love me + mom doesn\'t love me + mother doesn\'t love me + stepmom doesn\'t love me + stepmother doesn\'t love me + mom\'s boyfriend doesn\'t love me + mom boyfriend doesn\'t love me + mom\'s girlfriend doesn\'t love me + mom girlfriend doesn\'t love me + dad doesn\'t love me + father doesn\'t love me + stepdad doesn\'t love me + stepfather doesn\'t love me + dad\'s girlfriend doesn\'t love me + dad girlfriend doesn\'t love me + dad\'s boyfriend doesn\'t love me + dad boyfriend doesn\'t love me + grandma doesn\'t love me + grandpa doesn\'t love me + uncle doesn\'t love me + aunt doesn\'t love me + mom puts me down + mother puts me down + stepmom puts me down + stepmother puts me down + mom\'s boyfriend puts me down + mom boyfriend puts me down + mom\'s girlfriend puts me down + mom girlfriend puts me down + dad puts me down + father puts me down + stepdad puts me down + stepfather puts me down + dad\'s girlfriend puts me down + dad girlfriend puts me down + dad\'s boyfriend puts me down + dad boyfriend puts me down + grandma puts me down + grandpa puts me down + uncle puts me down + aunt puts me down + mom put me down + mother put me down + stepmom put me down + stepmother put me down + mom\'s boyfriend put me down + mom boyfriend put me down + mom\'s girlfriend put me down + mom girlfriend put me down + dad put me down + father put me down + stepdad put me down + stepfather put me down + dad\'s girlfriend put me down + dad girlfriend put me down + dad\'s boyfriend put me down + dad boyfriend put me down + grandma put me down + grandpa put me down + uncle put me down + aunt put me down + mom swears at me + mother swears at me + stepmom swears at me + stepmother swears at me + mom\'s boyfriend swears at me + mom boyfriend swears at me + mom\'s girlfriend swears at me + mom girlfriend swears at me + dad swears at me + father swears at me + stepdad swears at me + stepfather swears at me + dad\'s girlfriend swears at me + dad girlfriend swears at me + dad\'s boyfriend swears at me + dad boyfriend swears at me + grandma swears at me + grandpa swears at me + uncle swears at me + aunt swears at me + mom swear at me + mother swear at me + stepmom swear at me + stepmother swear at me + mom\'s boyfriend swear at me + mom boyfriend swear at me + mom\'s girlfriend swear at me + mom girlfriend swear at me + dad swear at me + father swear at me + stepdad swear at me + stepfather swear at me + dad\'s girlfriend swear at me + dad girlfriend swear at me + dad\'s boyfriend swear at me + dad boyfriend swear at me + grandma swear at me + grandpa swear at me + uncle swear at me + aunt swear at me + mom swore at me + mother swore at me + stepmom swore at me + stepmother swore at me + mom\'s boyfriend swore at me + mom boyfriend swore at me + mom\'s girlfriend swore at me + mom girlfriend swore at me + dad swore at me + father swore at me + stepdad swore at me + stepfather swore at me + dad\'s girlfriend swore at me + dad girlfriend swore at me + dad\'s boyfriend swore at me + dad boyfriend swore at me + grandma swore at me + grandpa swore at me + uncle swore at me + aunt swore at me + mom yell at me + mother yell at me + stepmom yell at me + stepmother yell at me + mom\'s boyfriend yell at me + mom boyfriend yell at me + mom\'s girlfriend yell at me + mom girlfriend yell at me + dad yell at me + father yell at me + stepdad yell at me + stepfather yell at me + dad\'s girlfriend yell at me + dad girlfriend yell at me + dad\'s boyfriend yell at me + dad boyfriend yell at me + grandma yell at me + grandpa yell at me + uncle yell at me + aunt yell at me + mom yells at me + mother yells at me + stepmom yells at me + stepmother yells at me + mom\'s boyfriend yells at me + mom boyfriend yells at me + mom\'s girlfriend yells at me + mom girlfriend yells at me + dad yells at me + father yells at me + stepdad yells at me + stepfather yells at me + dad\'s girlfriend yells at me + dad girlfriend yells at me + dad\'s boyfriend yells at me + dad boyfriend yells at me + grandma yells at me + grandpa yells at me + uncle yells at me + aunt yells at me + mom yelled at me + mother yelled at me + stepmom yelled at me + stepmother yelled at me + mom\'s boyfriend yelled at me + mom boyfriend yelled at me + mom\'s girlfriend yelled at me + mom girlfriend yelled at me + dad yelled at me + father yelled at me + stepdad yelled at me + stepfather yelled at me + dad\'s girlfriend yelled at me + dad girlfriend yelled at me + dad\'s boyfriend yelled at me + dad boyfriend yelled at me + grandma yelled at me + grandpa yelled at me + uncle yelled at me + aunt yelled at me + babysitter hate me + nanny hate me + babysitter hates me + nanny hates me + babysitter scare me + nanny scare me + babysitter scared me + nanny scared me + babysitter threaten me + nanny threaten me + babysitter threatens me + nanny threatens me + babysitter threatened me + nanny threatened me + babysitter puts me down + nanny puts me down + babysitter put me down + nanny put me down + babysitter swear at me + nanny swear at me + babysitter swears at me + nanny swears at me + babysitter swore at me + nanny swore at me + babysitter yell at me + nanny yell at me + babysitter yells at me + nanny yells at me + babysitter yelled at me + nanny yelled at me + mother\'s boyfriend hate me + mother boyfriend hate me + mother\'s girlfriend hate me + mother girlfriend hate me + father\'s boyfriend hate me + father boyfriend hate me + father\'s girlfriend hate me + father girlfriend hate me + parent hate me + parents hate me + mother\'s boyfriend hates me + mother boyfriend hates me + mother\'s girlfriend hates me + mother girlfriend hates me + father\'s boyfriend hates me + father boyfriend hates me + father\'s girlfriend hates me + father girlfriend hates me + parent hates me + parents hates me + mother\'s boyfriend scare me + mother boyfriend scare me + mother\'s girlfriend scare me + mother girlfriend scare me + father\'s boyfriend scare me + father boyfriend scare me + father\'s girlfriend scare me + father girlfriend scare me + parent scare me + parents scare me + mother\'s boyfriend scares me + mother boyfriend scares me + mother\'s girlfriend scares me + mother girlfriend scares me + father\'s boyfriend scares me + father boyfriend scares me + father\'s girlfriend scares me + father girlfriend scares me + parent scares me + parents scares me + mother\'s boyfriend scared me + mother boyfriend scared me + mother\'s girlfriend scared me + mother girlfriend scared me + father\'s boyfriend scared me + father boyfriend scared me + father\'s girlfriend scared me + father girlfriend scared me + parent scared me + parents scared me + mother\'s boyfriend threaten me + mother boyfriend threaten me + mother\'s girlfriend threaten me + mother girlfriend threaten me + father\'s boyfriend threaten me + father boyfriend threaten me + father\'s girlfriend threaten me + father girlfriend threaten me + parent threaten me + parents threaten me + mother\'s boyfriend threatens me + mother boyfriend threatens me + mother\'s girlfriend threatens me + mother girlfriend threatens me + father\'s boyfriend threatens me + father boyfriend threatens me + father\'s girlfriend threatens me + father girlfriend threatens me + parent threatens me + parents threatens me + mother\'s boyfriend threatened me + mother boyfriend threatened me + mother\'s girlfriend threatened me + mother girlfriend threatened me + father\'s boyfriend threatened me + father boyfriend threatened me + father\'s girlfriend threatened me + father girlfriend threatened me + parent threatened me + parents threatened me + mother\'s boyfriend does not care about me + mother boyfriend does not care about me + mother\'s girlfriend does not care about me + mother girlfriend does not care about me + father\'s boyfriend does not care about me + father boyfriend does not care about me + father\'s girlfriend does not care about me + father girlfriend does not care about me + parent does not care about me + parents does not care about me + mother\'s boyfriend doesnt care about me + mother boyfriend doesnt care about me + mother\'s girlfriend doesnt care about me + mother girlfriend doesnt care about me + father\'s boyfriend doesnt care about me + father boyfriend doesnt care about me + father\'s girlfriend doesnt care about me + father girlfriend doesnt care about me + parent doesnt care about me + parents doesnt care about me + mother\'s boyfriend doesn\'t care about me + mother boyfriend doesn\'t care about me + mother\'s girlfriend doesn\'t care about me + mother girlfriend doesn\'t care about me + father\'s boyfriend doesn\'t care about me + father boyfriend doesn\'t care about me + father\'s girlfriend doesn\'t care about me + father girlfriend doesn\'t care about me + parent doesn\'t care about me + parents doesn\'t care about me + mother\'s boyfriend does not love me + mother boyfriend does not love me + mother\'s girlfriend does not love me + mother girlfriend does not love me + father\'s boyfriend does not love me + father boyfriend does not love me + father\'s girlfriend does not love me + father girlfriend does not love me + parent does not love me + parents does not love me + mother\'s boyfriend doesnt love me + mother boyfriend doesnt love me + mother\'s girlfriend doesnt love me + mother girlfriend doesnt love me + father\'s boyfriend doesnt love me + father boyfriend doesnt love me + father\'s girlfriend doesnt love me + father girlfriend doesnt love me + parent doesnt love me + parents doesnt love me + mother\'s boyfriend doesn\'t love me + mother boyfriend doesn\'t love me + mother\'s girlfriend doesn\'t love me + mother girlfriend doesn\'t love me + father\'s boyfriend doesn\'t love me + father boyfriend doesn\'t love me + father\'s girlfriend doesn\'t love me + father girlfriend doesn\'t love me + parent doesn\'t love me + parents doesn\'t love me + mother\'s boyfriend puts me down + mother boyfriend puts me down + mother\'s girlfriend puts me down + mother girlfriend puts me down + father\'s boyfriend puts me down + father boyfriend puts me down + father\'s girlfriend puts me down + father girlfriend puts me down + parent puts me down + parents puts me down + mother\'s boyfriend put me down + mother boyfriend put me down + mother\'s girlfriend put me down + mother girlfriend put me down + father\'s boyfriend put me down + father boyfriend put me down + father\'s girlfriend put me down + father girlfriend put me down + parent put me down + parents put me down + mother\'s boyfriend swear at me + mother boyfriend swear at me + mother\'s girlfriend swear at me + mother girlfriend swear at me + father\'s boyfriend swear at me + father boyfriend swear at me + father\'s girlfriend swear at me + father girlfriend swear at me + parent swear at me + parents swear at me + mother\'s boyfriend swears at me + mother boyfriend swears at me + mother\'s girlfriend swears at me + mother girlfriend swears at me + father\'s boyfriend swears at me + father boyfriend swears at me + father\'s girlfriend swears at me + father girlfriend swears at me + parent swears at me + parents swears at me + mother\'s boyfriend swore at me + mother boyfriend swore at me + mother\'s girlfriend swore at me + mother girlfriend swore at me + father\'s boyfriend swore at me + father boyfriend swore at me + father\'s girlfriend swore at me + father girlfriend swore at me + parent swore at me + parents swore at me + mother\'s boyfriend yell at me + mother boyfriend yell at me + mother\'s girlfriend yell at me + mother girlfriend yell at me + father\'s boyfriend yell at me + father boyfriend yell at me + father\'s girlfriend yell at me + father girlfriend yell at me + parent yell at me + parents yell at me + mother\'s boyfriend yells at me + mother boyfriend yells at me + mother\'s girlfriend yells at me + mother girlfriend yells at me + father\'s boyfriend yells at me + father boyfriend yells at me + father\'s girlfriend yells at me + father girlfriend yells at me + parent yells at me + parents yells at me + mother\'s boyfriend yelled at me + mother boyfriend yelled at me + mother\'s girlfriend yelled at me + mother girlfriend yelled at me + father\'s boyfriend yelled at me + father boyfriend yelled at me + father\'s girlfriend yelled at me + father girlfriend yelled at me + parent yelled at me + parents yelled at me + mom scaring me + mother scaring me + stepmom scaring me + stepmother scaring me + mom\'s boyfriend scaring me + mom boyfriend scaring me + mom\'s girlfriend scaring me + mom girlfriend scaring me + dad scaring me + father scaring me + stepdad scaring me + stepfather scaring me + dad\'s girlfriend scaring me + dad girlfriend scaring me + dad\'s boyfriend scaring me + dad boyfriend scaring me + grandma scaring me + grandpa scaring me + uncle scaring me + aunt scaring me + mother\'s boyfriend scaring me + mother boyfriend scaring me + mother\'s girlfriend scaring me + mother girlfriend scaring me + father\'s boyfriend scaring me + father boyfriend scaring me + father\'s girlfriend scaring me + father girlfriend scaring me + parent scaring me + parents scaring me + nanny scaring me + babysitter scaring me + mom threatening me + mother threatening me + stepmom threatening me + stepmother threatening me + mom\'s boyfriend threatening me + mom boyfriend threatening me + mom\'s girlfriend threatening me + mom girlfriend threatening me + dad threatening me + father threatening me + stepdad threatening me + stepfather threatening me + dad\'s girlfriend threatening me + dad girlfriend threatening me + dad\'s boyfriend threatening me + dad boyfriend threatening me + grandma threatening me + grandpa threatening me + uncle threatening me + aunt threatening me + mother\'s boyfriend threatening me + mother boyfriend threatening me + mother\'s girlfriend threatening me + mother girlfriend threatening me + father\'s boyfriend threatening me + father boyfriend threatening me + father\'s girlfriend threatening me + father girlfriend threatening me + parent threatening me + parents threatening me + nanny threatening me + babysitter threatening me + mom putting me down + mother putting me down + stepmom putting me down + stepmother putting me down + mom\'s boyfriend putting me down + mom boyfriend putting me down + mom\'s girlfriend putting me down + mom girlfriend putting me down + dad putting me down + father putting me down + stepdad putting me down + stepfather putting me down + dad\'s girlfriend putting me down + dad girlfriend putting me down + dad\'s boyfriend putting me down + dad boyfriend putting me down + grandma putting me down + grandpa putting me down + uncle putting me down + aunt putting me down + mother\'s boyfriend putting me down + mother boyfriend putting me down + mother\'s girlfriend putting me down + mother girlfriend putting me down + father\'s boyfriend putting me down + father boyfriend putting me down + father\'s girlfriend putting me down + father girlfriend putting me down + parent putting me down + parents putting me down + nanny putting me down + babysitter putting me down + mom swearing at me + mother swearing at me + stepmom swearing at me + stepmother swearing at me + mom\'s boyfriend swearing at me + mom boyfriend swearing at me + mom\'s girlfriend swearing at me + mom girlfriend swearing at me + dad swearing at me + father swearing at me + stepdad swearing at me + stepfather swearing at me + dad\'s girlfriend swearing at me + dad girlfriend swearing at me + dad\'s boyfriend swearing at me + dad boyfriend swearing at me + grandma swearing at me + grandpa swearing at me + uncle swearing at me + aunt swearing at me + mother\'s boyfriend swearing at me + mother boyfriend swearing at me + mother\'s girlfriend swearing at me + mother girlfriend swearing at me + father\'s boyfriend swearing at me + father boyfriend swearing at me + father\'s girlfriend swearing at me + father girlfriend swearing at me + parent swearing at me + parents swearing at me + nanny swearing at me + babysitter swearing at me + "afraid of my mom" + "afraid of my mother" + "afraid of my stepmom" + "afraid of my stepmother" + "afraid of my mom\'s boyfriend" + "afraid of my mom boyfriend" + "afraid of my mom\'s girlfriend" + "afraid of my mom girlfriend" + "afraid of my dad" + "afraid of my father" + "afraid of my stepdad" + "afraid of my stepfather" + "afraid of my dad\'s girlfriend" + "afraid of my dad girlfriend" + "afraid of my dad\'s boyfriend" + "afraid of my dad boyfriend" + "afraid of my grandma" + "afraid of my grandpa" + "afraid of my uncle" + "afraid of my aunt" + "afraid of my mother\'s boyfriend" + "afraid of my mother boyfriend" + "afraid of my mother\'s girlfriend" + "afraid of my mother girlfriend" + "afraid of my father\'s boyfriend" + "afraid of my father boyfriend" + "afraid of my father\'s girlfriend" + "afraid of my father girlfriend" + "afraid of my parent" + "afraid of my parents" + "afraid of my nanny" + "afraid of my babysitter" + "scared of my mom" + "scared of my mother" + "scared of my stepmom" + "scared of my stepmother" + "scared of my mom\'s boyfriend" + "scared of my mom boyfriend" + "scared of my mom\'s girlfriend" + "scared of my mom girlfriend" + "scared of my dad" + "scared of my father" + "scared of my stepdad" + "scared of my stepfather" + "scared of my dad\'s girlfriend" + "scared of my dad girlfriend" + "scared of my dad\'s boyfriend" + "scared of my dad boyfriend" + "scared of my grandma" + "scared of my grandpa" + "scared of my uncle" + "scared of my aunt" + "scared of my mother\'s boyfriend" + "scared of my mother boyfriend" + "scared of my mother\'s girlfriend" + "scared of my mother girlfriend" + "scared of my father\'s boyfriend" + "scared of my father boyfriend" + "scared of my father\'s girlfriend" + "scared of my father girlfriend" + "scared of my parent" + "scared of my parents" + "scared of my nanny" + "scared of my babysitter" + mom yelling at me + mother yelling at me + stepmom yelling at me + stepmother yelling at me + mom\'s boyfriend yelling at me + mom boyfriend yelling at me + mom\'s girlfriend yelling at me + mom girlfriend yelling at me + dad yelling at me + father yelling at me + stepdad yelling at me + stepfather yelling at me + dad\'s girlfriend yelling at me + dad girlfriend yelling at me + dad\'s boyfriend yelling at me + dad boyfriend yelling at me + grandma yelling at me + grandpa yelling at me + uncle yelling at me + aunt yelling at me + mother\'s boyfriend yelling at me + mother boyfriend yelling at me + mother\'s girlfriend yelling at me + mother girlfriend yelling at me + father\'s boyfriend yelling at me + father boyfriend yelling at me + father\'s girlfriend yelling at me + father girlfriend yelling at me + parent yelling at me + parents yelling at me + nanny yelling at me + babysitter yelling at me

**First-hand account child abuse search terms**

**Sexual Abuse**

mom touch my + mother touch my + stepmom touch my + stepmother touch my + mom\'s boyfriend touch my + mom boyfriend touch my + mom\'s girlfriend touch my + mom girlfriend touch my + dad touch my + father touch my + stepdad touch my + stepfather touch my + dad\'s girlfriend touch my + dad girlfriend touch my + dad\'s boyfriend touch my + dad boyfriend touch my + grandma touch my + grandpa touch my + brother touch my + sister touch my + uncle touch my + aunt touch my + mom touches my + mother touches my + stepmom touches my + stepmother touches my + mom\'s boyfriend touches my + mom boyfriend touches my + mom\'s girlfriend touches my + mom girlfriend touches my + dad touches my + father touches my + stepdad touches my + stepfather touches my + dad\'s girlfriend touches my + dad girlfriend touches my + dad\'s boyfriend touches my + dad boyfriend touches my + grandma touches my + grandpa touches my + brother touches my + sister touches my + uncle touches my + aunt touches my + mom touched my + mother touched my + stepmom touched my + stepmother touched my + mom\'s boyfriend touched my + mom boyfriend touched my + mom\'s girlfriend touched my + mom girlfriend touched my + dad touched my + father touched my + stepdad touched my + stepfather touched my + dad\'s girlfriend touched my + dad girlfriend touched my + dad\'s boyfriend touched my + dad boyfriend touched my + grandma touched my + grandpa touched my + brother touched my + sister touched my + uncle touched my + aunt touched my + mom rub my + mother rub my + stepmom rub my + stepmother rub my + mom\'s boyfriend rub my + mom boyfriend rub my + mom\'s girlfriend rub my + mom girlfriend rub my + dad rub my + father rub my + stepdad rub my + stepfather rub my + dad\'s girlfriend rub my + dad girlfriend rub my + dad\'s boyfriend rub my + dad boyfriend rub my + grandma rub my + grandpa rub my + brother rub my + sister rub my + uncle rub my + aunt rub my + mom rubs my + mother rubs my + stepmom rubs my + stepmother rubs my + mom\'s boyfriend rubs my + mom boyfriend rubs my + mom\'s girlfriend rubs my + mom girlfriend rubs my + dad rubs my + father rubs my + stepdad rubs my + stepfather rubs my + dad\'s girlfriend rubs my + dad girlfriend rubs my + dad\'s boyfriend rubs my + dad boyfriend rubs my + grandma rubs my + grandpa rubs my + brother rubs my + sister rubs my + uncle rubs my + aunt rubs my + mom rubbed my + mother rubbed my + stepmom rubbed my + stepmother rubbed my + mom\'s boyfriend rubbed my + mom boyfriend rubbed my + mom\'s girlfriend rubbed my + mom girlfriend rubbed my + dad rubbed my + father rubbed my + stepdad rubbed my + stepfather rubbed my + dad\'s girlfriend rubbed my + dad girlfriend rubbed my + dad\'s boyfriend rubbed my + dad boyfriend rubbed my + grandma rubbed my + grandpa rubbed my + brother rubbed my + sister rubbed my + uncle rubbed my + aunt rubbed my + mom makes me touch their + mother makes me touch their + stepmom makes me touch their + stepmother makes me touch their + mom\'s boyfriend makes me touch their + mom boyfriend makes me touch their + mom\'s girlfriend makes me touch their + mom girlfriend makes me touch their + dad makes me touch their + father makes me touch their + stepdad makes me touch their + stepfather makes me touch their + dad\'s girlfriend makes me touch their + dad girlfriend makes me touch their + dad\'s boyfriend makes me touch their + dad boyfriend makes me touch their + grandma makes me touch their + grandpa makes me touch their + brother makes me touch their + sister makes me touch their + uncle makes me touch their + aunt makes me touch their + mom makes me touch there + mother makes me touch there + stepmom makes me touch there + stepmother makes me touch there + mom\'s boyfriend makes me touch there + mom boyfriend makes me touch there + mom\'s girlfriend makes me touch there + mom girlfriend makes me touch there + dad makes me touch there + father makes me touch there + stepdad makes me touch there + stepfather makes me touch there + dad\'s girlfriend makes me touch there + dad girlfriend makes me touch there + dad\'s boyfriend makes me touch there + dad boyfriend makes me touch there + grandma makes me touch there + grandpa makes me touch there + brother makes me touch there + sister makes me touch there + uncle makes me touch there + aunt makes me touch there + mom made me touch their + mother made me touch their + stepmom made me touch their + stepmother made me touch their + mom\'s boyfriend made me touch their + mom boyfriend made me touch their + mom\'s girlfriend made me touch their + mom girlfriend made me touch their + dad made me touch their + father made me touch their + stepdad made me touch their + stepfather made me touch their + dad\'s girlfriend made me touch their + dad girlfriend made me touch their + dad\'s boyfriend made me touch their + dad boyfriend made me touch their + grandma made me touch their + grandpa made me touch their + brother made me touch their + sister made me touch their + uncle made me touch their + aunt made me touch their + mom made me touch there + mother made me touch there + stepmom made me touch there + stepmother made me touch there + mom\'s boyfriend made me touch there + mom boyfriend made me touch there + mom\'s girlfriend made me touch there + mom girlfriend made me touch there + dad made me touch there + father made me touch there + stepdad made me touch there + stepfather made me touch there + dad\'s girlfriend made me touch there + dad girlfriend made me touch there + dad\'s boyfriend made me touch there + dad boyfriend made me touch there + grandma made me touch there + grandpa made me touch there + brother made me touch there + sister made me touch there + uncle made me touch there + aunt made me touch there + mom molest me + mother molest me + stepmom molest me + stepmother molest me + mom\'s boyfriend molest me + mom boyfriend molest me + mom\'s girlfriend molest me + mom girlfriend molest me + dad molest me + father molest me + stepdad molest me + stepfather molest me + dad\'s girlfriend molest me + dad girlfriend molest me + dad\'s boyfriend molest me + dad boyfriend molest me + grandma molest me + grandpa molest me + brother molest me + sister molest me + uncle molest me + aunt molest me + mom molested me + mother molested me + stepmom molested me + stepmother molested me + mom\'s boyfriend molested me + mom boyfriend molested me + mom\'s girlfriend molested me + mom girlfriend molested me + dad molested me + father molested me + stepdad molested me + stepfather molested me + dad\'s girlfriend molested me + dad girlfriend molested me + dad\'s boyfriend molested me + dad boyfriend molested me + grandma molested me + grandpa molested me + brother molested me + sister molested me + uncle molested me + aunt molested me + mom rape me + mother rape me + stepmom rape me + stepmother rape me + mom\'s boyfriend rape me + mom boyfriend rape me + mom\'s girlfriend rape me + mom girlfriend rape me + dad rape me + father rape me + stepdad rape me + stepfather rape me + dad\'s girlfriend rape me + dad girlfriend rape me + dad\'s boyfriend rape me + dad boyfriend rape me + grandma rape me + grandpa rape me + brother rape me + sister rape me + uncle rape me + aunt rape me + mom raped me + mother raped me + stepmom raped me + stepmother raped me + mom\'s boyfriend raped me + mom boyfriend raped me + mom\'s girlfriend raped me + mom girlfriend raped me + dad raped me + father raped me + stepdad raped me + stepfather raped me + dad\'s girlfriend raped me + dad girlfriend raped me + dad\'s boyfriend raped me + dad boyfriend raped me + grandma raped me + grandpa raped me + brother raped me + sister raped me + uncle raped me + aunt raped me + mom have sex with me + mother have sex with me + stepmom have sex with me + stepmother have sex with me + mom\'s boyfriend have sex with me + mom boyfriend have sex with me + mom\'s girlfriend have sex with me + mom girlfriend have sex with me + dad have sex with me + father have sex with me + stepdad have sex with me + stepfather have sex with me + dad\'s girlfriend have sex with me + dad girlfriend have sex with me + dad\'s boyfriend have sex with me + dad boyfriend have sex with me + grandma have sex with me + grandpa have sex with me + brother have sex with me + sister have sex with me + uncle have sex with me + aunt have sex with me + mom had sex with me + mother had sex with me + stepmom had sex with me + stepmother had sex with me + mom\'s boyfriend had sex with me + mom boyfriend had sex with me + mom\'s girlfriend had sex with me + mom girlfriend had sex with me + dad had sex with me + father had sex with me + stepdad had sex with me + stepfather had sex with me + dad\'s girlfriend had sex with me + dad girlfriend had sex with me + dad\'s boyfriend had sex with me + dad boyfriend had sex with me + grandma had sex with me + grandpa had sex with me + brother had sex with me + sister had sex with me + uncle had sex with me + aunt had sex with me + babysitter touch my + nanny touch my + babysitter touched my + nanny touched my + babysitter touches my + nanny touches my + babysitter rub my + nanny rub my + babysitter rubs my + nanny rubs my + babysitter rubbed my + nanny rubbed my + babysitter makes me touch their + nanny makes me touch their + babysitter made me touch their + nanny made me touch their + babysitter makes me touch there + nanny makes me touch there + babysitter made me touch there + nanny made me touch there + babysitter molest me + nanny molest me + babysitter molested me + nanny molested me + babysitter rape me + nanny rape me + babysitter raped me + nanny raped me + babysitter have sex with me + nanny have sex with me + babysitter had sex with me + nanny had sex with me + mother\'s boyfriend touch my + mother boyfriend touch my + mother\'s girlfriend touch my + mother girlfriend touch my + father\'s boyfriend touch my + father boyfriend touch my + father\'s girlfriend touch my + father girlfriend touch my + parent touch my + parents touch my + mother\'s boyfriend touches my + mother boyfriend touches my + mother\'s girlfriend touches my + mother girlfriend touches my + father\'s boyfriend touches my + father boyfriend touches my + father\'s girlfriend touches my + father girlfriend touches my + parent touches my + parents touches my + mother\'s boyfriend touched my + mother boyfriend touched my + mother\'s girlfriend touched my + mother girlfriend touched my + father\'s boyfriend touched my + father boyfriend touched my + father\'s girlfriend touched my + father girlfriend touched my + parent touched my + parents touched my + mother\'s boyfriend rub my + mother boyfriend rub my + mother\'s girlfriend rub my + mother girlfriend rub my + father\'s boyfriend rub my + father boyfriend rub my + father\'s girlfriend rub my + father girlfriend rub my + parent rub my + parents rub my + mother\'s boyfriend rubs my + mother boyfriend rubs my + mother\'s girlfriend rubs my + mother girlfriend rubs my + father\'s boyfriend rubs my + father boyfriend rubs my + father\'s girlfriend rubs my + father girlfriend rubs my + parent rubs my + parents rubs my + mother\'s boyfriend rubbed my + mother boyfriend rubbed my + mother\'s girlfriend rubbed my + mother girlfriend rubbed my + father\'s boyfriend rubbed my + father boyfriend rubbed my + father\'s girlfriend rubbed my + father girlfriend rubbed my + parent rubbed my + parents rubbed my + mother\'s boyfriend molest me + mother boyfriend molest me + mother\'s girlfriend molest me + mother girlfriend molest me + father\'s boyfriend molest me + father boyfriend molest me + father\'s girlfriend molest me + father girlfriend molest me + parent molest me + parents molest me + mother\'s boyfriend molested me + mother boyfriend molested me + mother\'s girlfriend molested me + mother girlfriend molested me + father\'s boyfriend molested me + father boyfriend molested me + father\'s girlfriend molested me + father girlfriend molested me + parent molested me + parents molested me + mother\'s boyfriend rape me + mother boyfriend rape me + mother\'s girlfriend rape me + mother girlfriend rape me + father\'s boyfriend rape me + father boyfriend rape me + father\'s girlfriend rape me + father girlfriend rape me + parent rape me + parents rape me + mother\'s boyfriend raped me + mother boyfriend raped me + mother\'s girlfriend raped me + mother girlfriend raped me + father\'s boyfriend raped me + father boyfriend raped me + father\'s girlfriend raped me + father girlfriend raped me + parent raped me + parents raped me + mother\'s boyfriend have sex with me + mother boyfriend have sex with me + mother\'s girlfriend have sex with me + mother girlfriend have sex with me + father\'s boyfriend have sex with me + father boyfriend have sex with me + father\'s girlfriend have sex with me + father girlfriend have sex with me + parent have sex with me + parents have sex with me + mother\'s boyfriend had sex with me + mother boyfriend had sex with me + mother\'s girlfriend had sex with me + mother girlfriend had sex with me + father\'s boyfriend had sex with me + father boyfriend had sex with me + father\'s girlfriend had sex with me + father girlfriend had sex with me + parent had sex with me + parents had sex with me + mother\'s boyfriend makes me touch their + mother boyfriend makes me touch their + mother\'s girlfriend makes me touch their + mother girlfriend makes me touch their + father\'s boyfriend makes me touch their + father boyfriend makes me touch their + father\'s girlfriend makes me touch their + father girlfriend makes me touch their + parent makes me touch their + parents makes me touch their + mother\'s boyfriend makes me touch there + mother boyfriend makes me touch there + mother\'s girlfriend makes me touch there + mother girlfriend makes me touch there + father\'s boyfriend makes me touch there + father boyfriend makes me touch there + father\'s girlfriend makes me touch there + father girlfriend makes me touch there + parent makes me touch there + parents makes me touch there + mother\'s boyfriend made me touch their + mother boyfriend made me touch their + mother\'s girlfriend made me touch their + mother girlfriend made me touch their + father\'s boyfriend made me touch their + father boyfriend made me touch their + father\'s girlfriend made me touch their + father girlfriend made me touch their + parent made me touch their + parents made me touch their + mother\'s boyfriend made me touch there + mother boyfriend made me touch there + mother\'s girlfriend made me touch there + mother girlfriend made me touch there + father\'s boyfriend made me touch there + father boyfriend made me touch there + father\'s girlfriend made me touch there + father girlfriend made me touch there + parent made me touch there + parents made me touch there + mom touching my + mother touching my + stepmom touching my + stepmother touching my + mom\'s boyfriend touching my + mom boyfriend touching my + mom\'s girlfriend touching my + mom girlfriend touching my + dad touching my + father touching my + stepdad touching my + stepfather touching my + dad\'s girlfriend touching my + dad girlfriend touching my + dad\'s boyfriend touching my + dad boyfriend touching my + grandma touching my + grandpa touching my + uncle touching my + aunt touching my + mother\'s boyfriend touching my + mother boyfriend touching my + mother\'s girlfriend touching my + mother girlfriend touching my + father\'s boyfriend touching my + father boyfriend touching my + father\'s girlfriend touching my + father girlfriend touching my + parent touching my + parents touching my + nanny touching my + babysitter touching my + brother touching my + sister touching my + mom rubbing my + mother rubbing my + stepmom rubbing my + stepmother rubbing my + mom\'s boyfriend rubbing my + mom boyfriend rubbing my + mom\'s girlfriend rubbing my + mom girlfriend rubbing my + dad rubbing my + father rubbing my + stepdad rubbing my + stepfather rubbing my + dad\'s girlfriend rubbing my + dad girlfriend rubbing my + dad\'s boyfriend rubbing my + dad boyfriend rubbing my + grandma rubbing my + grandpa rubbing my + uncle rubbing my + aunt rubbing my + mother\'s boyfriend rubbing my + mother boyfriend rubbing my + mother\'s girlfriend rubbing my + mother girlfriend rubbing my + father\'s boyfriend rubbing my + father boyfriend rubbing my + father\'s girlfriend rubbing my + father girlfriend rubbing my + parent rubbing my + parents rubbing my + nanny rubbing my + babysitter rubbing my + brother rubbing my + sister rubbing my + mom molesting me + mother molesting me + stepmom molesting me + stepmother molesting me + mom\'s boyfriend molesting me + mom boyfriend molesting me + mom\'s girlfriend molesting me + mom girlfriend molesting me + dad molesting me + father molesting me + stepdad molesting me + stepfather molesting me + dad\'s girlfriend molesting me + dad girlfriend molesting me + dad\'s boyfriend molesting me + dad boyfriend molesting me + grandma molesting me + grandpa molesting me + uncle molesting me + aunt molesting me + mother\'s boyfriend molesting me + mother boyfriend molesting me + mother\'s girlfriend molesting me + mother girlfriend molesting me + father\'s boyfriend molesting me + father boyfriend molesting me + father\'s girlfriend molesting me + father girlfriend molesting me + parent molesting me + parents molesting me + nanny molesting me + babysitter molesting me + brother molesting me + sister molesting me + mom molests me + mother molests me + stepmom molests me + stepmother molests me + mom\'s boyfriend molests me + mom boyfriend molests me + mom\'s girlfriend molests me + mom girlfriend molests me + dad molests me + father molests me + stepdad molests me + stepfather molests me + dad\'s girlfriend molests me + dad girlfriend molests me + dad\'s boyfriend molests me + dad boyfriend molests me + grandma molests me + grandpa molests me + uncle molests me + aunt molests me + mother\'s boyfriend molests me + mother boyfriend molests me + mother\'s girlfriend molests me + mother girlfriend molests me + father\'s boyfriend molests me + father boyfriend molests me + father\'s girlfriend molests me + father girlfriend molests me + parent molests me + parents molests me + nanny molests me + babysitter molests me + brother molests me + sister molests me + mom raping me + mother raping me + stepmom raping me + stepmother raping me + mom\'s boyfriend raping me + mom boyfriend raping me + mom\'s girlfriend raping me + mom girlfriend raping me + dad raping me + father raping me + stepdad raping me + stepfather raping me + dad\'s girlfriend raping me + dad girlfriend raping me + dad\'s boyfriend raping me + dad boyfriend raping me + grandma raping me + grandpa raping me + uncle raping me + aunt raping me + mother\'s boyfriend raping me + mother boyfriend raping me + mother\'s girlfriend raping me + mother girlfriend raping me + father\'s boyfriend raping me + father boyfriend raping me + father\'s girlfriend raping me + father girlfriend raping me + parent raping me + parents raping me + nanny raping me + babysitter raping me + brother raping me + sister raping me + mom rapes me + mother rapes me + stepmom rapes me + stepmother rapes me + mom\'s boyfriend rapes me + mom boyfriend rapes me + mom\'s girlfriend rapes me + mom girlfriend rapes me + dad rapes me + father rapes me + stepdad rapes me + stepfather rapes me + dad\'s girlfriend rapes me + dad girlfriend rapes me + dad\'s boyfriend rapes me + dad boyfriend rapes me + grandma rapes me + grandpa rapes me + uncle rapes me + aunt rapes me + mother\'s boyfriend rapes me + mother boyfriend rapes me + mother\'s girlfriend rapes me + mother girlfriend rapes me + father\'s boyfriend rapes me + father boyfriend rapes me + father\'s girlfriend rapes me + father girlfriend rapes me + parent rapes me + parents rapes me + nanny rapes me + babysitter rapes me + brother rapes me + sister rapes me + mom has sex with me + mother has sex with me + stepmom has sex with me + stepmother has sex with me + mom\'s boyfriend has sex with me + mom boyfriend has sex with me + mom\'s girlfriend has sex with me + mom girlfriend has sex with me + dad has sex with me + father has sex with me + stepdad has sex with me + stepfather has sex with me + dad\'s girlfriend has sex with me + dad girlfriend has sex with me + dad\'s boyfriend has sex with me + dad boyfriend has sex with me + grandma has sex with me + grandpa has sex with me + uncle has sex with me + aunt has sex with me + mother\'s boyfriend has sex with me + mother boyfriend has sex with me + mother\'s girlfriend has sex with me + mother girlfriend has sex with me + father\'s boyfriend has sex with me + father boyfriend has sex with me + father\'s girlfriend has sex with me + father girlfriend has sex with me + parent has sex with me + parents has sex with me + nanny has sex with me + babysitter has sex with me + brother has sex with me + sister has sex with me + mom having sex with me + mother having sex with me + stepmom having sex with me + stepmother having sex with me + mom\'s boyfriend having sex with me + mom boyfriend having sex with me + mom\'s girlfriend having sex with me + mom girlfriend having sex with me + dad having sex with me + father having sex with me + stepdad having sex with me + stepfather having sex with me + dad\'s girlfriend having sex with me + dad girlfriend having sex with me + dad\'s boyfriend having sex with me + dad boyfriend having sex with me + grandma having sex with me + grandpa having sex with me + uncle having sex with me + aunt having sex with me + mother\'s boyfriend having sex with me + mother boyfriend having sex with me + mother\'s girlfriend having sex with me + mother girlfriend having sex with me + father\'s boyfriend having sex with me + father boyfriend having sex with me + father\'s girlfriend having sex with me + father girlfriend having sex with me + parent having sex with me + parents having sex with me + nanny having sex with me + babysitter having sex with me + brother having sex with me + sister having sex with me + mom makes me touch her + mother makes me touch her + stepmom makes me touch her + stepmother makes me touch her + mom\'s boyfriend makes me touch his + mom boyfriend makes me touch his + mom\'s girlfriend makes me touch her + mom girlfriend makes me touch her + dad makes me touch his + father makes me touch his + stepdad makes me touch his + stepfather makes me touch his + dad\'s girlfriend makes me touch her + dad girlfriend makes me touch her + dad\'s boyfriend makes me touch his + dad boyfriend makes me touch his + grandma makes me touch her + grandpa makes me touch his + uncle makes me touch his + aunt makes me touch her + mother\'s boyfriend makes me touch his + mother boyfriend makes me touch his + mother\'s girlfriend makes me touch her + mother girlfriend makes me touch her + father\'s boyfriend makes me touch his + father boyfriend makes me touch his + father\'s girlfriend makes me touch her + father girlfriend makes me touch her + parent makes me touch her + parent makes me touch his + nanny makes me touch her + nanny makes me touch his + babysitter makes me touch his + babysitter makes me touch her + brother makes me touch his + sister makes me touch her + mom made me touch her + mother made me touch her + stepmom made me touch her + stepmother made me touch her + mom\'s boyfriend made me touch his + mom boyfriend made me touch his + mom\'s girlfriend made me touch her + mom girlfriend made me touch her + dad made me touch his + father made me touch his + stepdad made me touch his + stepfather made me touch his + dad\'s girlfriend made me touch her + dad girlfriend made me touch her + dad\'s boyfriend made me touch his + dad boyfriend made me touch his + grandma made me touch her + grandpa made me touch his + uncle made me touch his + aunt made me touch her + mother\'s boyfriend made me touch his + mother boyfriend made me touch his + mother\'s girlfriend made me touch her + mother girlfriend made me touch her + father\'s boyfriend made me touch his + father boyfriend made me touch his + father\'s girlfriend made me touch her + father girlfriend made me touch her + parent made me touch her + parent made me touch his + nanny made me touch her + nanny made me touch his + babysitter made me touch his + babysitter made me touch her + brother made me touch his + sister made me touch her + mom making me touch her + mother making me touch her + stepmom making me touch her + stepmother making me touch her + mom\'s boyfriend making me touch his + mom boyfriend making me touch his + mom\'s girlfriend making me touch her + mom girlfriend making me touch her + dad making me touch his + father making me touch his + stepdad making me touch his + stepfather making me touch his + dad\'s girlfriend making me touch her + dad girlfriend making me touch her + dad\'s boyfriend making me touch his + dad boyfriend making me touch his + grandma making me touch her + grandpa making me touch his + uncle making me touch his + aunt making me touch her + mother\'s boyfriend making me touch his + mother boyfriend making me touch his + mother\'s girlfriend making me touch her + mother girlfriend making me touch her + father\'s boyfriend making me touch his + father boyfriend making me touch his + father\'s girlfriend making me touch her + father girlfriend making me touch her + parent making me touch her + parent making me touch his + nanny making me touch her + nanny making me touch his + babysitter making me touch his + babysitter making me touch her + brother making me touch his + sister making me touch her + mom make me touch her + mother make me touch her + stepmom make me touch her + stepmother make me touch her + mom\'s boyfriend make me touch his + mom boyfriend make me touch his + mom\'s girlfriend make me touch her + mom girlfriend make me touch her + dad make me touch his + father make me touch his + stepdad make me touch his + stepfather make me touch his + dad\'s girlfriend make me touch her + dad girlfriend make me touch her + dad\'s boyfriend make me touch his + dad boyfriend make me touch his + grandma make me touch her + grandpa make me touch his + uncle make me touch his + aunt make me touch her + mother\'s boyfriend make me touch his + mother boyfriend make me touch his + mother\'s girlfriend make me touch her + mother girlfriend make me touch her + father\'s boyfriend make me touch his + father boyfriend make me touch his + father\'s girlfriend make me touch her + father girlfriend make me touch her + parent make me touch her + parent make me touch his + nanny make me touch her + nanny make me touch his + babysitter make me touch his + babysitter make me touch her + brother make me touch his + sister make me touch her + mom making me touch their + mother making me touch their + stepmom making me touch their + stepmother making me touch their + mom\'s boyfriend making me touch their + mom boyfriend making me touch their + mom\'s girlfriend making me touch their + mom girlfriend making me touch their + dad making me touch their + father making me touch their + stepdad making me touch their + stepfather making me touch their + dad\'s girlfriend making me touch their + dad girlfriend making me touch their + dad\'s boyfriend making me touch their + dad boyfriend making me touch their + grandma making me touch their + grandpa making me touch their + uncle making me touch their + aunt making me touch their + mother\'s boyfriend making me touch their + mother boyfriend making me touch their + mother\'s girlfriend making me touch their + mother girlfriend making me touch their + father\'s boyfriend making me touch their + father boyfriend making me touch their + father\'s girlfriend making me touch their + father girlfriend making me touch their + parent making me touch their + parents making me touch their + nanny making me touch their + babysitter making me touch their + brother making me touch their + sister making me touch their + mom making me touch there + mother making me touch there + stepmom making me touch there + stepmother making me touch there + mom\'s boyfriend making me touch there + mom boyfriend making me touch there + mom\'s girlfriend making me touch there + mom girlfriend making me touch there + dad making me touch there + father making me touch there + stepdad making me touch there + stepfather making me touch there + dad\'s girlfriend making me touch there + dad girlfriend making me touch there + dad\'s boyfriend making me touch there + dad boyfriend making me touch there + grandma making me touch there + grandpa making me touch there + uncle making me touch there + aunt making me touch there + mother\'s boyfriend making me touch there + mother boyfriend making me touch there + mother\'s girlfriend making me touch there + mother girlfriend making me touch there + father\'s boyfriend making me touch there + father boyfriend making me touch there + father\'s girlfriend making me touch there + father girlfriend making me touch there + parent making me touch there + parents making me touch there + nanny making me touch there + babysitter making me touch there + brother making me touch there + sister making me touch there + mom make me touch their + mother make me touch their + stepmom make me touch their + stepmother make me touch their + mom\'s boyfriend make me touch their + mom boyfriend make me touch their + mom\'s girlfriend make me touch their + mom girlfriend make me touch their + dad make me touch their + father make me touch their + stepdad make me touch their + stepfather make me touch their + dad\'s girlfriend make me touch their + dad girlfriend make me touch their + dad\'s boyfriend make me touch their + dad boyfriend make me touch their + grandma make me touch their + grandpa make me touch their + uncle make me touch their + aunt make me touch their + mother\'s boyfriend make me touch their + mother boyfriend make me touch their + mother\'s girlfriend make me touch their + mother girlfriend make me touch their + father\'s boyfriend make me touch their + father boyfriend make me touch their + father\'s girlfriend make me touch their + father girlfriend make me touch their + parent make me touch their + parents make me touch their + nanny make me touch their + babysitter make me touch their + brother make me touch their + sister make me touch their + mom make me touch there + mother make me touch there + stepmom make me touch there + stepmother make me touch there + mom\'s boyfriend make me touch there + mom boyfriend make me touch there + mom\'s girlfriend make me touch there + mom girlfriend make me touch there + dad make me touch there + father make me touch there + stepdad make me touch there + stepfather make me touch there + dad\'s girlfriend make me touch there + dad girlfriend make me touch there + dad\'s boyfriend make me touch there + dad boyfriend make me touch there + grandma make me touch there + grandpa make me touch there + uncle make me touch there + aunt make me touch there + mother\'s boyfriend make me touch there + mother boyfriend make me touch there + mother\'s girlfriend make me touch there + mother girlfriend make me touch there + father\'s boyfriend make me touch there + father boyfriend make me touch there + father\'s girlfriend make me touch there + father girlfriend make me touch there + parent make me touch there + parents make me touch there + nanny make me touch there + babysitter make me touch there + brother make me touch there + sister make me touch there + mom force me touch their + mother force me touch their + stepmom force me touch their + stepmother force me touch their + mom\'s boyfriend force me touch their + mom boyfriend force me touch their + mom\'s girlfriend force me touch their + mom girlfriend force me touch their + dad force me touch their + father force me touch their + stepdad force me touch their + stepfather force me touch their + dad\'s girlfriend force me touch their + dad girlfriend force me touch their + dad\'s boyfriend force me touch their + dad boyfriend force me touch their + grandma force me touch their + grandpa force me touch their + uncle force me touch their + aunt force me touch their + mother\'s boyfriend force me touch their + mother boyfriend force me touch their + mother\'s girlfriend force me touch their + mother girlfriend force me touch their + father\'s boyfriend force me touch their + father boyfriend force me touch their + father\'s girlfriend force me touch their + father girlfriend force me touch their + parent force me touch their + parents force me touch their + nanny force me touch their + babysitter force me touch their + brother force me touch their + sister force me touch their + mom force me touch there + mother force me touch there + stepmom force me touch there + stepmother force me touch there + mom\'s boyfriend force me touch there + mom boyfriend force me touch there + mom\'s girlfriend force me touch there + mom girlfriend force me touch there + dad force me touch there + father force me touch there + stepdad force me touch there + stepfather force me touch there + dad\'s girlfriend force me touch there + dad girlfriend force me touch there + dad\'s boyfriend force me touch there + dad boyfriend force me touch there + grandma force me touch there + grandpa force me touch there + uncle force me touch there + aunt force me touch there + mother\'s boyfriend force me touch there + mother boyfriend force me touch there + mother\'s girlfriend force me touch there + mother girlfriend force me touch there + father\'s boyfriend force me touch there + father boyfriend force me touch there + father\'s girlfriend force me touch there + father girlfriend force me touch there + parent force me touch there + parents force me touch there + nanny force me touch there + babysitter force me touch there + brother force me touch there + sister force me touch there + mom forces me touch their + mother forces me touch their + stepmom forces me touch their + stepmother forces me touch their + mom\'s boyfriend forces me touch their + mom boyfriend forces me touch their + mom\'s girlfriend forces me touch their + mom girlfriend forces me touch their + dad forces me touch their + father forces me touch their + stepdad forces me touch their + stepfather forces me touch their + dad\'s girlfriend forces me touch their + dad girlfriend forces me touch their + dad\'s boyfriend forces me touch their + dad boyfriend forces me touch their + grandma forces me touch their + grandpa forces me touch their + uncle forces me touch their + aunt forces me touch their + mother\'s boyfriend forces me touch their + mother boyfriend forces me touch their + mother\'s girlfriend forces me touch their + mother girlfriend forces me touch their + father\'s boyfriend forces me touch their + father boyfriend forces me touch their + father\'s girlfriend forces me touch their + father girlfriend forces me touch their + parent forces me touch their + parents forces me touch their + nanny forces me touch their + babysitter forces me touch their + brother forces me touch their + sister forces me touch their + mom forces me touch there + mother forces me touch there + stepmom forces me touch there + stepmother forces me touch there + mom\'s boyfriend forces me touch there + mom boyfriend forces me touch there + mom\'s girlfriend forces me touch there + mom girlfriend forces me touch there + dad forces me touch there + father forces me touch there + stepdad forces me touch there + stepfather forces me touch there + dad\'s girlfriend forces me touch there + dad girlfriend forces me touch there + dad\'s boyfriend forces me touch there + dad boyfriend forces me touch there + grandma forces me touch there + grandpa forces me touch there + uncle forces me touch there + aunt forces me touch there + mother\'s boyfriend forces me touch there + mother boyfriend forces me touch there + mother\'s girlfriend forces me touch there + mother girlfriend forces me touch there + father\'s boyfriend forces me touch there + father boyfriend forces me touch there + father\'s girlfriend forces me touch there + father girlfriend forces me touch there + parent forces me touch there + parents forces me touch there + nanny forces me touch there + babysitter forces me touch there + brother forces me touch there + sister forces me touch there + mom forced me touch their + mother forced me touch their + stepmom forced me touch their + stepmother forced me touch their + mom\'s boyfriend forced me touch their + mom boyfriend forced me touch their + mom\'s girlfriend forced me touch their + mom girlfriend forced me touch their + dad forced me touch their + father forced me touch their + stepdad forced me touch their + stepfather forced me touch their + dad\'s girlfriend forced me touch their + dad girlfriend forced me touch their + dad\'s boyfriend forced me touch their + dad boyfriend forced me touch their + grandma forced me touch their + grandpa forced me touch their + uncle forced me touch their + aunt forced me touch their + mother\'s boyfriend forced me touch their + mother boyfriend forced me touch their + mother\'s girlfriend forced me touch their + mother girlfriend forced me touch their + father\'s boyfriend forced me touch their + father boyfriend forced me touch their + father\'s girlfriend forced me touch their + father girlfriend forced me touch their + parent forced me touch their + parents forced me touch their + nanny forced me touch their + babysitter forced me touch their + brother forced me touch their + sister forced me touch their + mom forced me touch there + mother forced me touch there + stepmom forced me touch there + stepmother forced me touch there + mom\'s boyfriend forced me touch there + mom boyfriend forced me touch there + mom\'s girlfriend forced me touch there + mom girlfriend forced me touch there + dad forced me touch there + father forced me touch there + stepdad forced me touch there + stepfather forced me touch there + dad\'s girlfriend forced me touch there + dad girlfriend forced me touch there + dad\'s boyfriend forced me touch there + dad boyfriend forced me touch there + grandma forced me touch there + grandpa forced me touch there + uncle forced me touch there + aunt forced me touch there + mother\'s boyfriend forced me touch there + mother boyfriend forced me touch there + mother\'s girlfriend forced me touch there + mother girlfriend forced me touch there + father\'s boyfriend forced me touch there + father boyfriend forced me touch there + father\'s girlfriend forced me touch there + father girlfriend forced me touch there + parent forced me touch there + parents forced me touch there + nanny forced me touch there + babysitter forced me touch there + brother forced me touch there + sister forced me touch there + mom forcing me touch their + mother forcing me touch their + stepmom forcing me touch their + stepmother forcing me touch their + mom\'s boyfriend forcing me touch their + mom boyfriend forcing me touch their + mom\'s girlfriend forcing me touch their + mom girlfriend forcing me touch their + dad forcing me touch their + father forcing me touch their + stepdad forcing me touch their + stepfather forcing me touch their + dad\'s girlfriend forcing me touch their + dad girlfriend forcing me touch their + dad\'s boyfriend forcing me touch their + dad boyfriend forcing me touch their + grandma forcing me touch their + grandpa forcing me touch their + uncle forcing me touch their + aunt forcing me touch their + mother\'s boyfriend forcing me touch their + mother boyfriend forcing me touch their + mother\'s girlfriend forcing me touch their + mother girlfriend forcing me touch their + father\'s boyfriend forcing me touch their + father boyfriend forcing me touch their + father\'s girlfriend forcing me touch their + father girlfriend forcing me touch their + parent forcing me touch their + parents forcing me touch their + nanny forcing me touch their + babysitter forcing me touch their + brother forcing me touch their + sister forcing me touch their + mom forcing me touch there + mother forcing me touch there + stepmom forcing me touch there + stepmother forcing me touch there + mom\'s boyfriend forcing me touch there + mom boyfriend forcing me touch there + mom\'s girlfriend forcing me touch there + mom girlfriend forcing me touch there + dad forcing me touch there + father forcing me touch there + stepdad forcing me touch there + stepfather forcing me touch there + dad\'s girlfriend forcing me touch there + dad girlfriend forcing me touch there + dad\'s boyfriend forcing me touch there + dad boyfriend forcing me touch there + grandma forcing me touch there + grandpa forcing me touch there + uncle forcing me touch there + aunt forcing me touch there + mother\'s boyfriend forcing me touch there + mother boyfriend forcing me touch there + mother\'s girlfriend forcing me touch there + mother girlfriend forcing me touch there + father\'s boyfriend forcing me touch there + father boyfriend forcing me touch there + father\'s girlfriend forcing me touch there + father girlfriend forcing me touch there + parent forcing me touch there + parents forcing me touch there + nanny forcing me touch there + babysitter forcing me touch there + brother forcing me touch there + sister forcing me touch there + mom force me touch her + mother force me touch her + stepmom force me touch her + stepmother force me touch her + mom\'s boyfriend force me touch his + mom boyfriend force me touch his + mom\'s girlfriend force me touch her + mom girlfriend force me touch her + dad force me touch his + father force me touch his + stepdad force me touch his + stepfather force me touch his + dad\'s girlfriend force me touch her + dad girlfriend force me touch her + dad\'s boyfriend force me touch his + dad boyfriend force me touch his + grandma force me touch her + grandpa force me touch his + uncle force me touch his + aunt force me touch her + mother\'s boyfriend force me touch his + mother boyfriend force me touch his + mother\'s girlfriend force me touch her + mother girlfriend force me touch her + father\'s boyfriend force me touch his + father boyfriend force me touch his + father\'s girlfriend force me touch her + father girlfriend force me touch her + parent force me touch her + parent force me touch his + nanny force me touch her + nanny force me touch his + babysitter force me touch his + babysitter force me touch her + brother force me touch his + sister force me touch her + mom forces me touch her + mother forces me touch her + stepmom forces me touch her + stepmother forces me touch her + mom\'s boyfriend forces me touch his + mom boyfriend forces me touch his + mom\'s girlfriend forces me touch her + mom girlfriend forces me touch her + dad forces me touch his + father forces me touch his + stepdad forces me touch his + stepfather forces me touch his + dad\'s girlfriend forces me touch her + dad girlfriend forces me touch her + dad\'s boyfriend forces me touch his + dad boyfriend forces me touch his + grandma forces me touch her + grandpa forces me touch his + uncle forces me touch his + aunt forces me touch her + mother\'s boyfriend forces me touch his + mother boyfriend forces me touch his + mother\'s girlfriend forces me touch her + mother girlfriend forces me touch her + father\'s boyfriend forces me touch his + father boyfriend forces me touch his + father\'s girlfriend forces me touch her + father girlfriend forces me touch her + parent forces me touch her + parent forces me touch his + nanny forces me touch her + nanny forces me touch his + babysitter forces me touch his + babysitter forces me touch her + brother forces me touch his + sister forces me touch her + mom forcing me touch her + mother forcing me touch her + stepmom forcing me touch her + stepmother forcing me touch her + mom\'s boyfriend forcing me touch his + mom boyfriend forcing me touch his + mom\'s girlfriend forcing me touch her + mom girlfriend forcing me touch her + dad forcing me touch his + father forcing me touch his + stepdad forcing me touch his + stepfather forcing me touch his + dad\'s girlfriend forcing me touch her + dad girlfriend forcing me touch her + dad\'s boyfriend forcing me touch his + dad boyfriend forcing me touch his + grandma forcing me touch her + grandpa forcing me touch his + uncle forcing me touch his + aunt forcing me touch her + mother\'s boyfriend forcing me touch his + mother boyfriend forcing me touch his + mother\'s girlfriend forcing me touch her + mother girlfriend forcing me touch her + father\'s boyfriend forcing me touch his + father boyfriend forcing me touch his + father\'s girlfriend forcing me touch her + father girlfriend forcing me touch her + parent forcing me touch her + parent forcing me touch his + nanny forcing me touch her + nanny forcing me touch his + babysitter forcing me touch his + babysitter forcing me touch her + brother forcing me touch his + sister forcing me touch her + mom forced me touch her + mother forced me touch her + stepmom forced me touch her + stepmother forced me touch her + mom\'s boyfriend forced me touch his + mom boyfriend forced me touch his + mom\'s girlfriend forced me touch her + mom girlfriend forced me touch her + dad forced me touch his + father forced me touch his + stepdad forced me touch his + stepfather forced me touch his + dad\'s girlfriend forced me touch her + dad girlfriend forced me touch her + dad\'s boyfriend forced me touch his + dad boyfriend forced me touch his + grandma forced me touch her + grandpa forced me touch his + uncle forced me touch his + aunt forced me touch her + mother\'s boyfriend forced me touch his + mother boyfriend forced me touch his + mother\'s girlfriend forced me touch her + mother girlfriend forced me touch her + father\'s boyfriend forced me touch his + father boyfriend forced me touch his + father\'s girlfriend forced me touch her + father girlfriend forced me touch her + parent forced me touch her + parent forced me touch his + nanny forced me touch her + nanny forced me touch his + babysitter forced me touch his + babysitter forced me touch her + brother forced me touch his + sister forced me touch her

**First-hand account child abuse search terms**

**Other searches**

child abuse hotline
