# Supplementary material for: Excess Google Searches for Child Abuse and Intimate Partner Violence During the COVID-19 Pandemic: Infoveillance Approach
Source: J Med Internet Res. 2022 Jun 13;24(6):e36445. doi: 10.2196/36445 (PMC9202515; doi:10.2196/36445)
Supplement: Multimedia Appendix 2 [file jmir_v24i6e36445_app2.docx]

**Appendix S2 to the paper “Excess Google Searches for Child Abuse and Intimate Partner Violence During the Covid-19 Pandemic: An Infoveillance Approach”**

**Figure S1: Tile map of average search volume for child abuse victimization as a function of time**

**
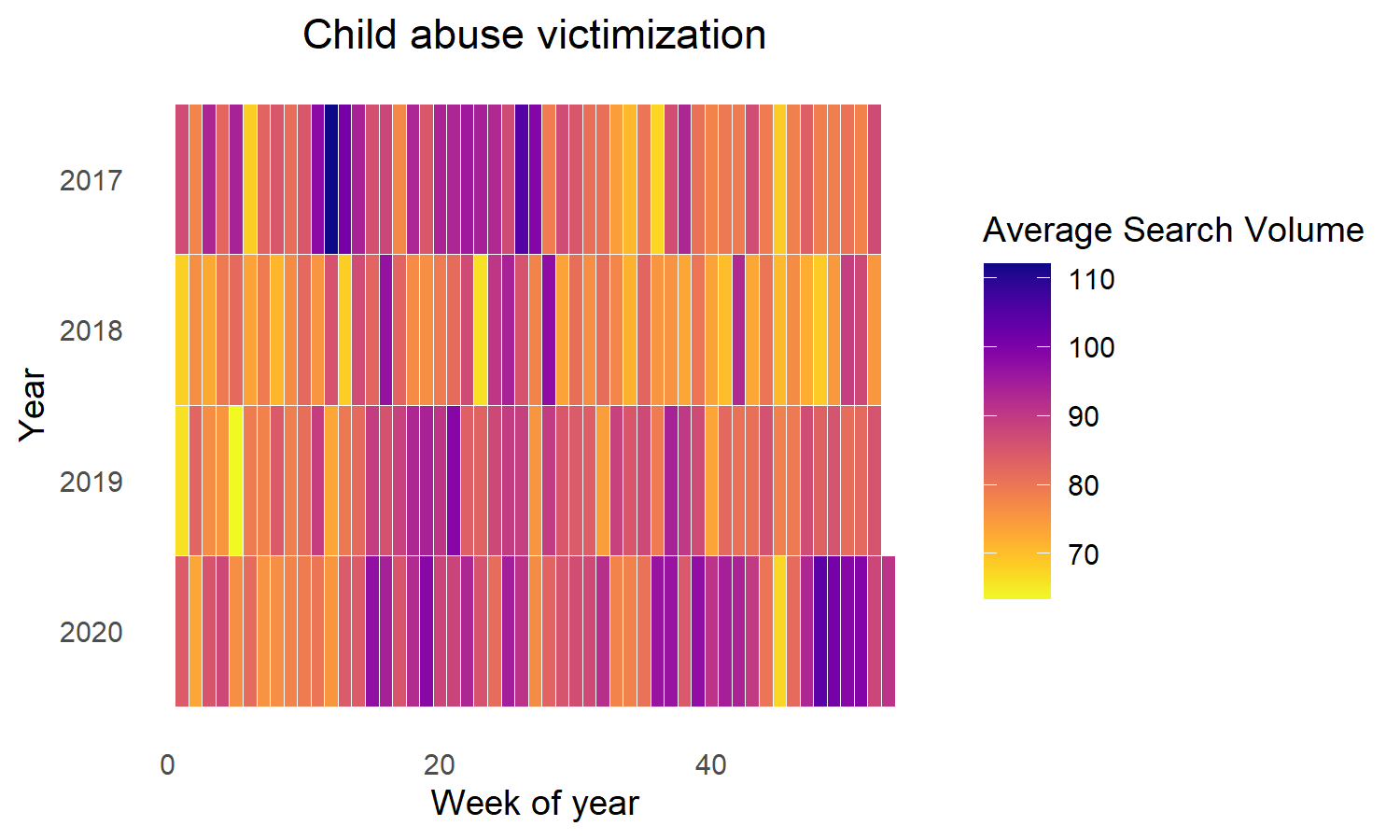
**

**Figure S2: Tile map of average search volume for child-witnessed IPV as a function of time**

**
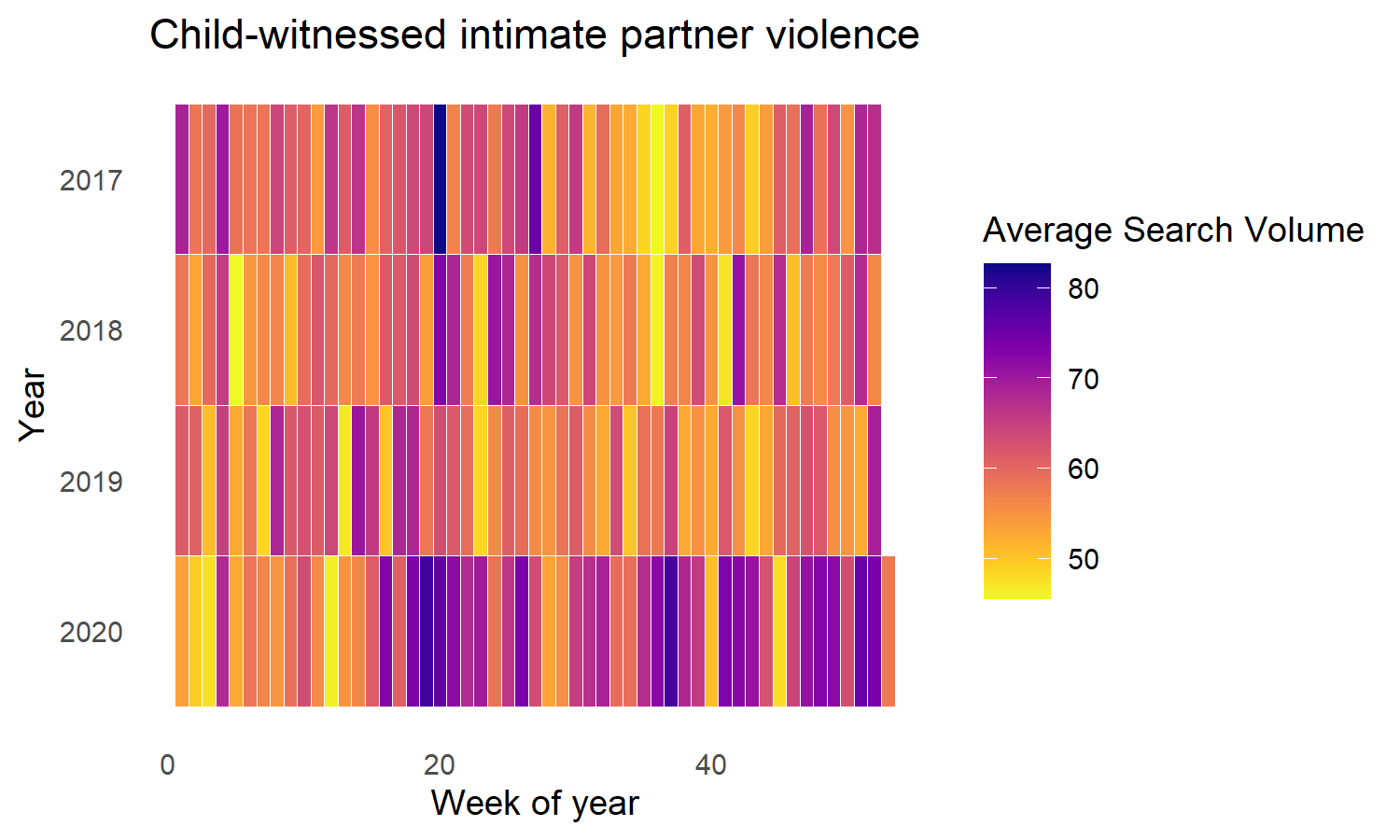
**

**Figure S3: Tile map of average search volume for IPV as a function of time**

**
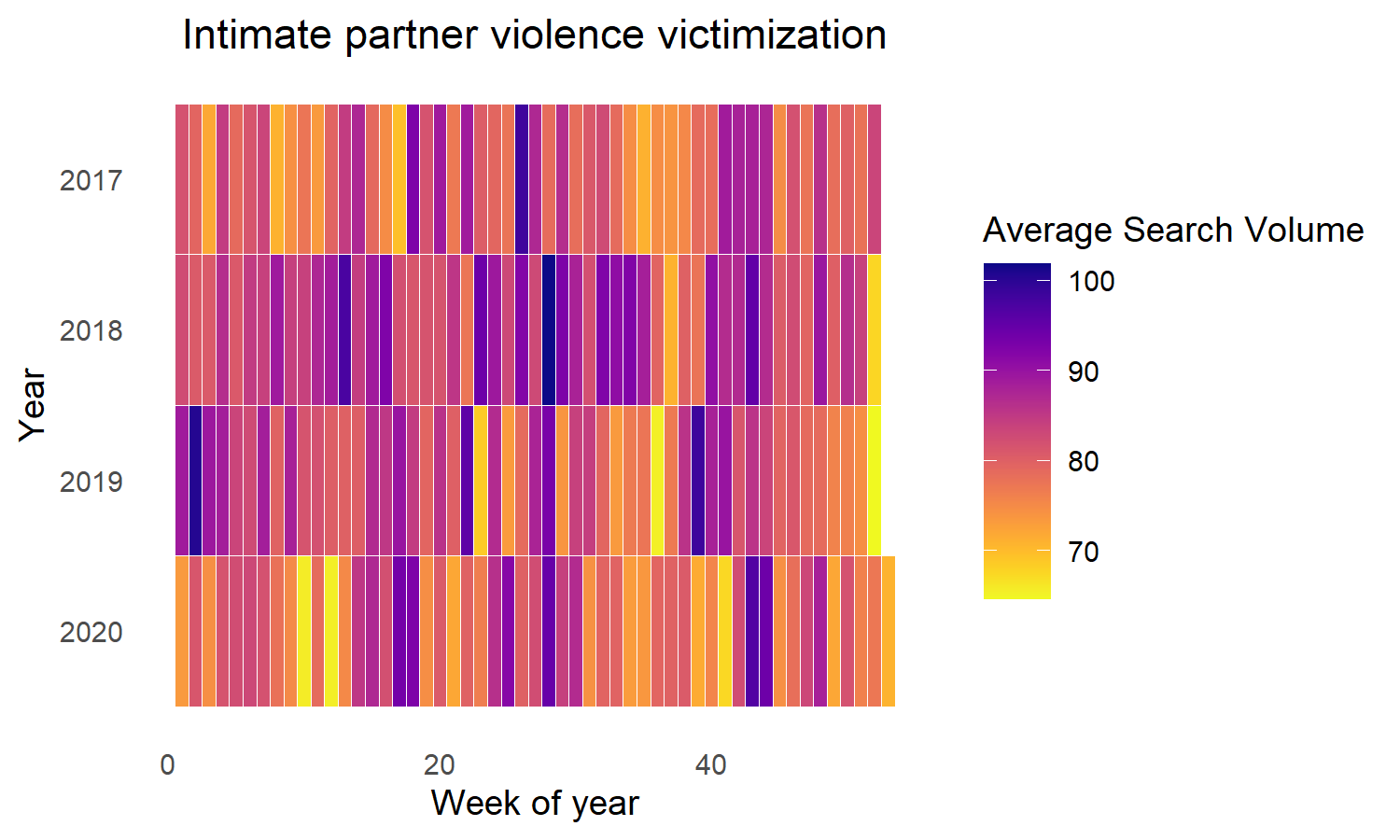
**

**Regression model assumptions**

**Figure S4: Assessment of Normality (quantile quantile plots)**


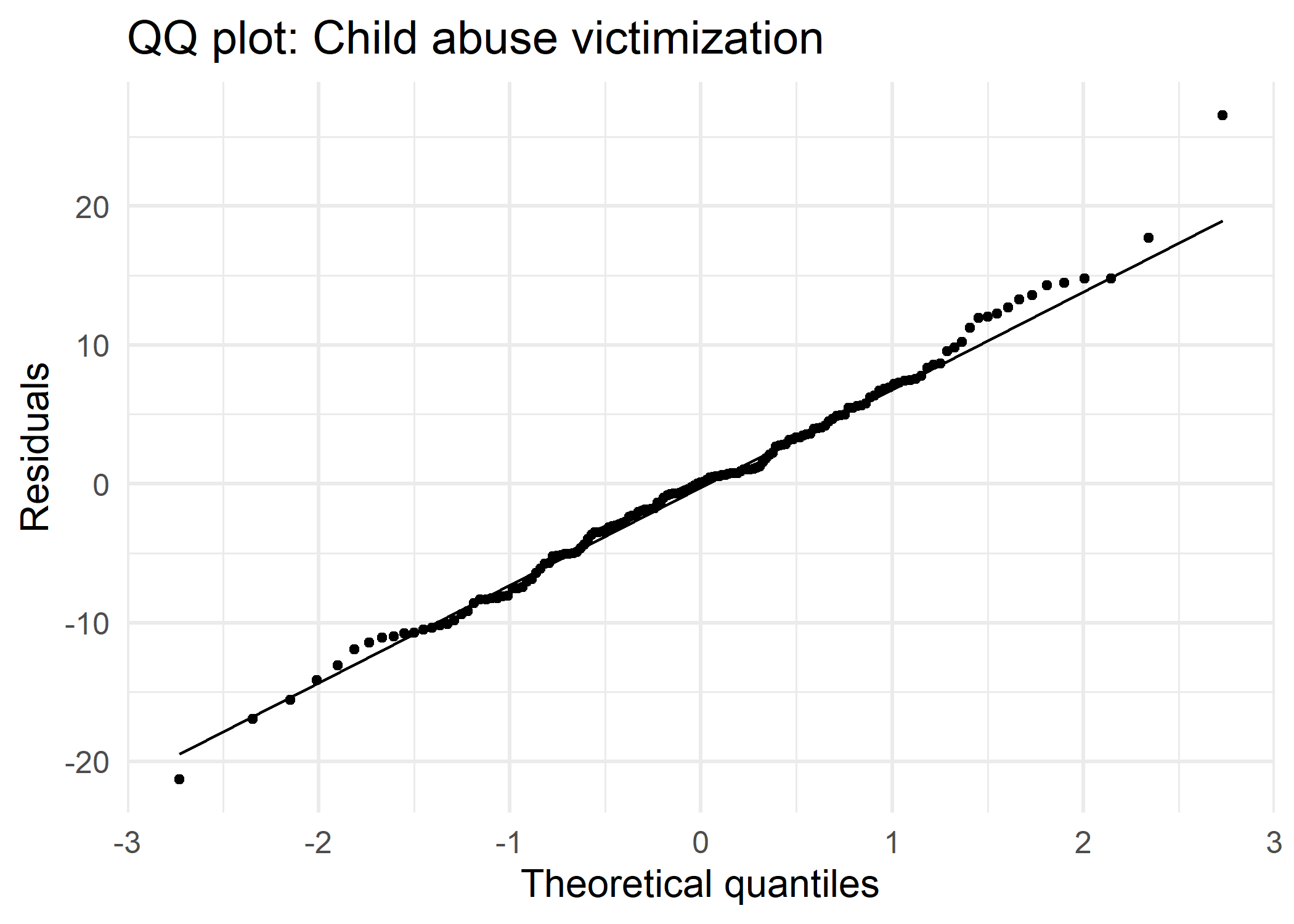

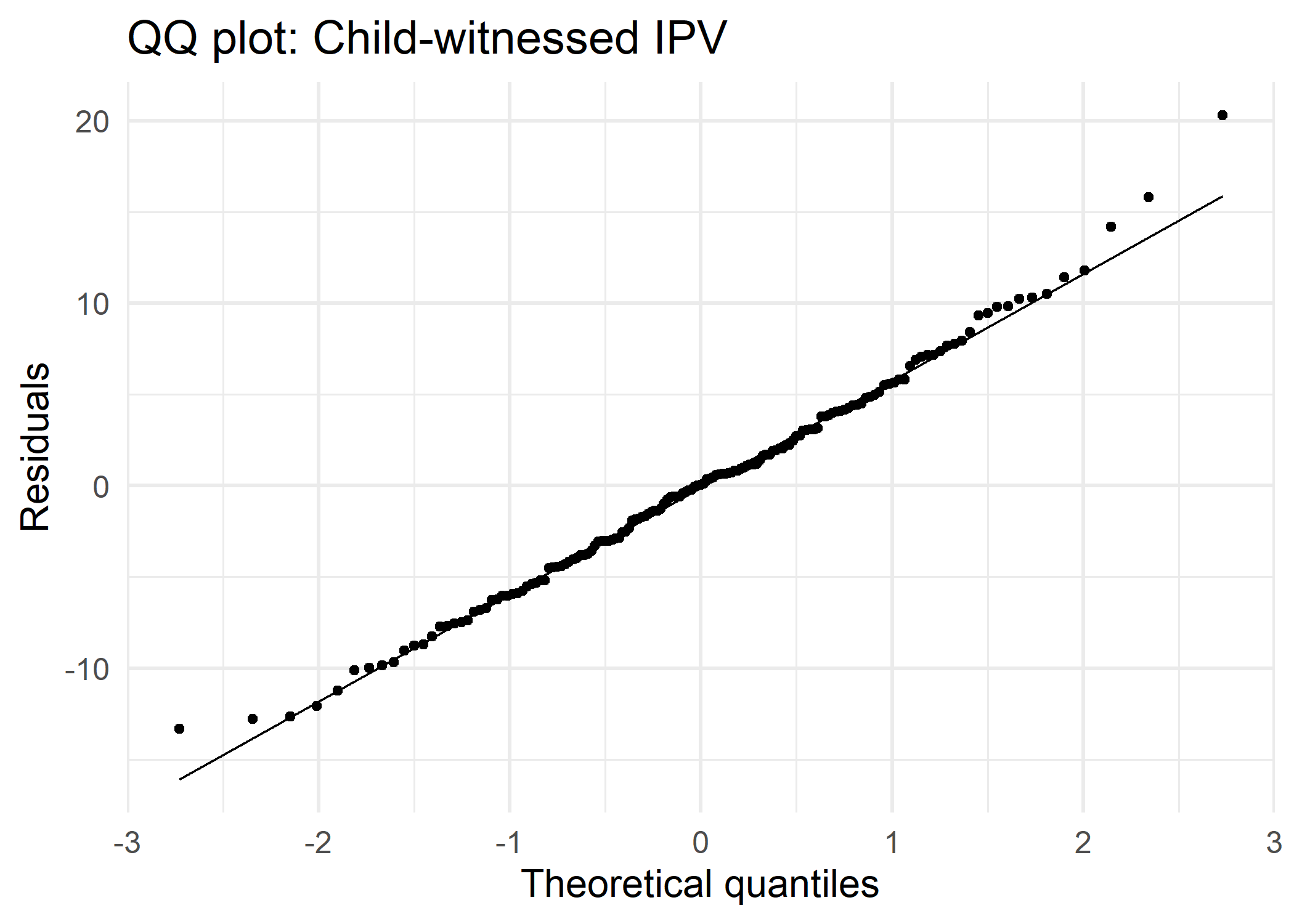


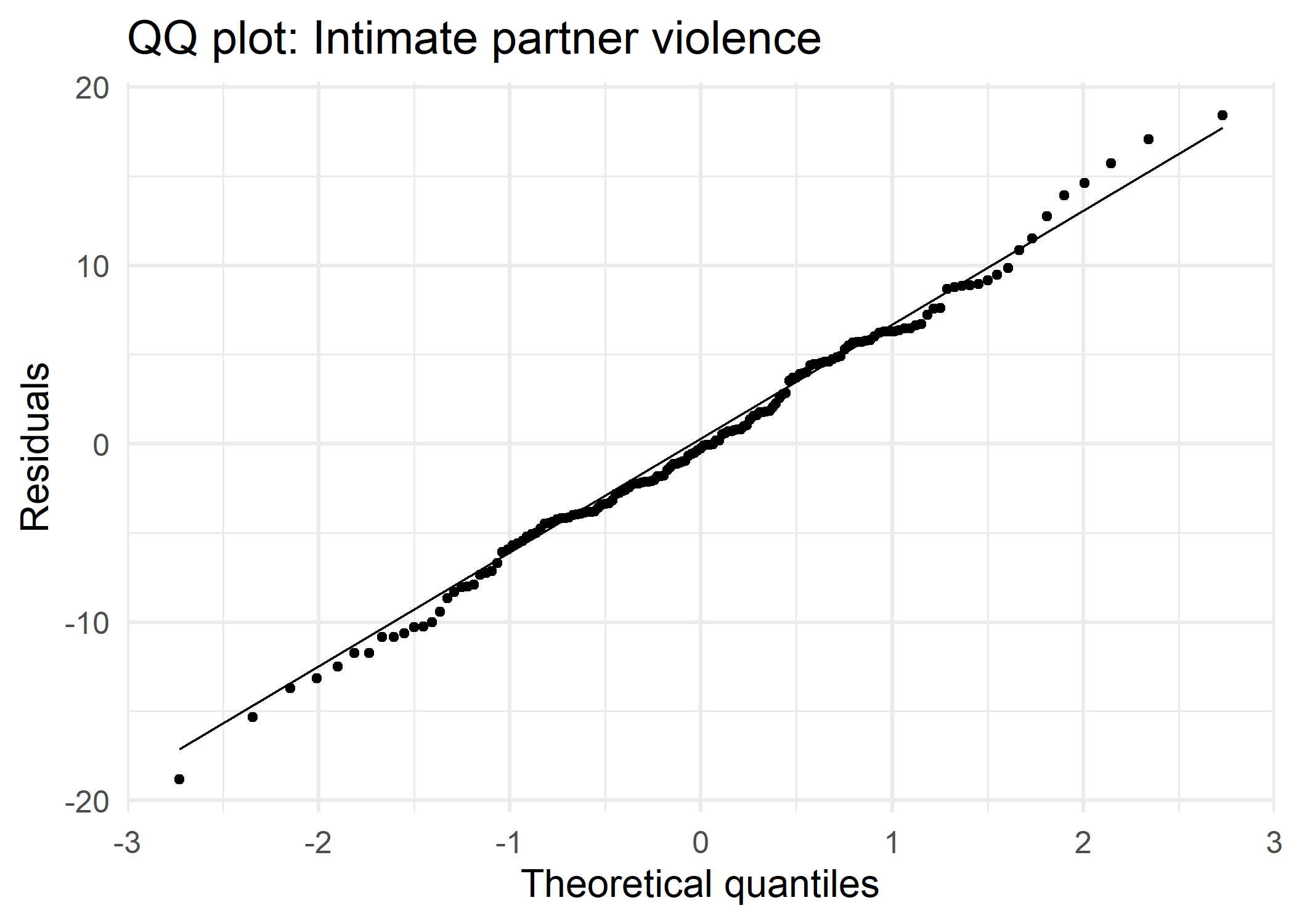


These plots support normality of these data (all lie close to the line)

**Figure S5: Homoskedasticity** (**fitted vs. residual plots)**


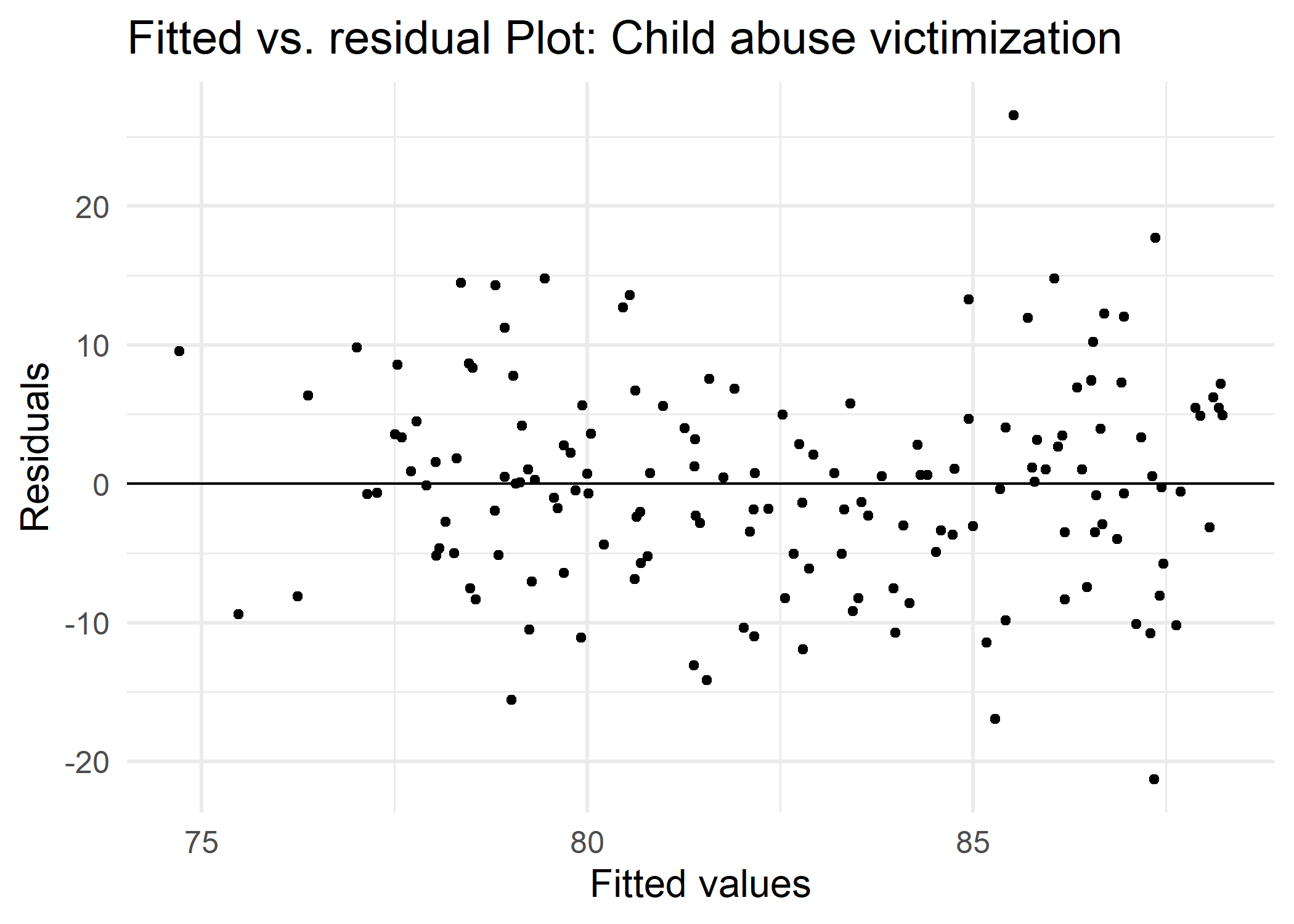

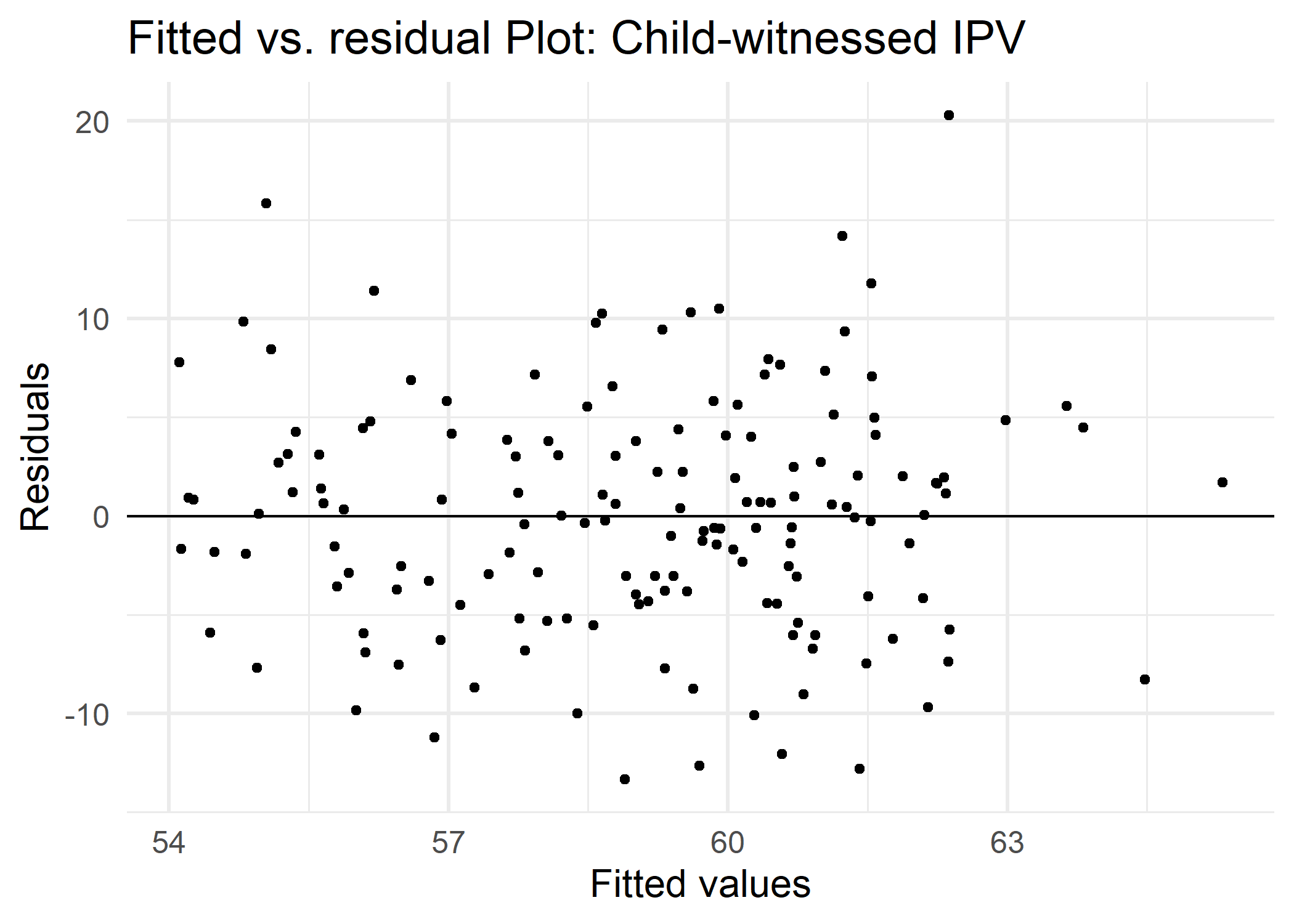


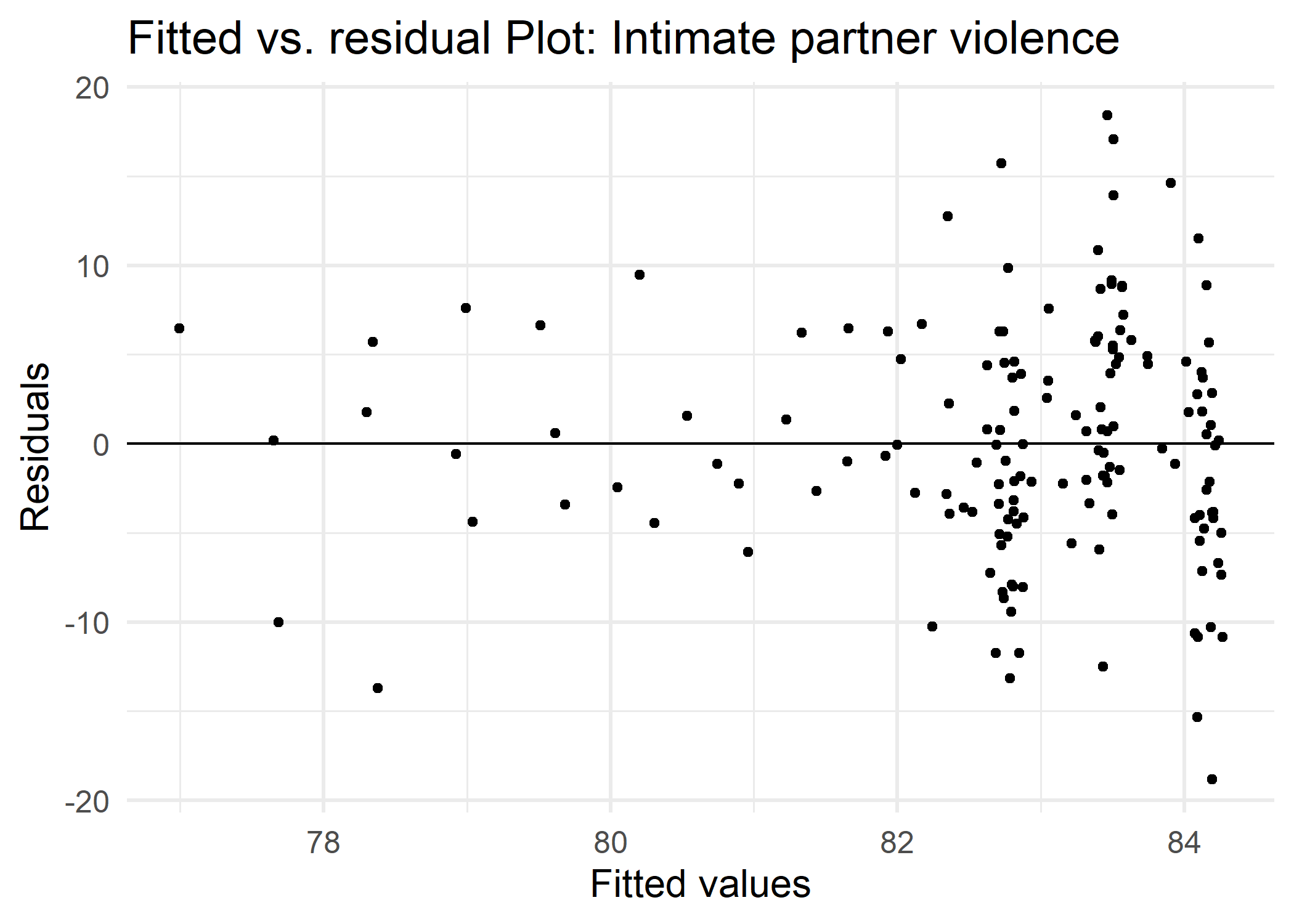


To assess homoskedasticity, we examine plots of the fitted vs. residual values. Homoskedasticity is supported when there is no clear pattern. There is no clear pattern in the plots for child abuse victimization and child-witnessed IPV. For IPV, there are fewer data points with smaller fitted values (i.e., to the left of x=82) and this reflects that this model’s predictions were basically flat within each of the three years of data, with fewer lower fitted values in Nov-Dec of each year. This first gives the appearance of increasing variance as a function of increasing fitted values, although this pattern is informed by few points in the region with less data so we did not reject this assumption of using linear regression.

**Figure S6: Investigation of outliers**


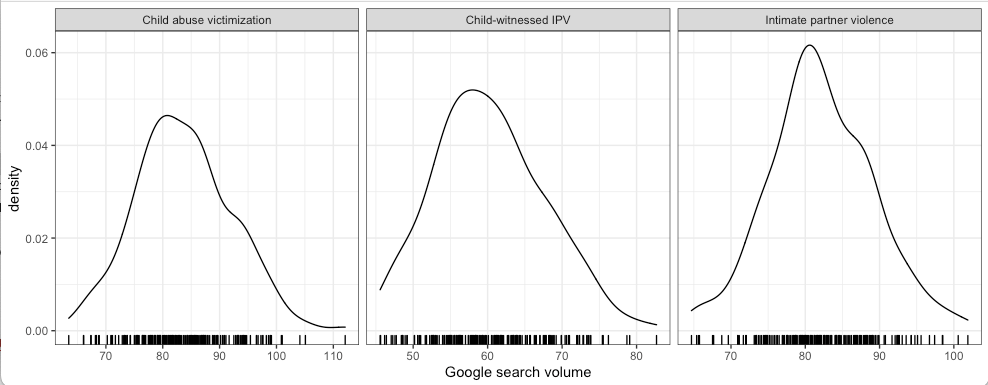


To check this assumption, we examined the distribution of the outcome variables using density plots. The tick marks along the x axis shows the location of each data point across all weeks included in the sample. No outlier data points were detected for the outcome. We did not look at the independent variables since these did not contain outliers by definition.

**Multicollinearity**

We didn’t assess multicollinearity because the independent variables included were year and week which are not collinear by definition.
